# Bioengineered perfused human brain microvascular networks enhance neural progenitor cell survival, neurogenesis, and maturation

**DOI:** 10.1126/sciadv.aaz9499

**Published:** 2023-05-10

**Authors:** Max A. Winkelman, Guohao Dai

**Affiliations:** Department of Bioengineering, Northeastern University, Boston, MA, USA.

## Abstract

Neural progenitor cells (NPCs) have the capability to self-renew and differentiate into neurons and glial cells. In the adult brain, NPCs are found near brain microvascular networks (BMVNs) in specialized microenvironments called the neurovascular niche (NVN). Although several in vitro NVN models have been previously reported, most do not properly recapitulate the intimate cellular interactions between NPCs and perfused brain microvessels. Here, we developed perfused BMVNs composed of primary human brain endothelial cells, pericytes, and astrocytes within microfluidic devices. When induced pluripotent stem cell–derived NPCs were introduced into BMVNs, we found that NPC survival, neurogenesis, and maturation were enhanced. The application of flow during BMVN coculture was also beneficial for neuron differentiation. Collectively, our work highlighted the important role of BMVNs and flow in NPC self-renewal and neurogenesis, as well as demonstrated our model’s potential to study the biological and physical interactions of human NVN in vitro.

## Teaser

Neural stem cells proliferate and differentiate into mature neurons around perfused blood vessels.

## INTRODUCTION

Neurodegenerative diseases result in the decline of human cognition, usually due to the death or dysfunction of neurons. Examples of pervasive neurodegenerative diseases include Alzheimer’s disease, Parkinson’s disease, Huntington’s disease, amyotrophic lateral sclerosis, stroke, and brain tumors. In the United States, neurological disorders afflict millions of patients each year and contributed to a total economic burden of approximately $789 billion in 2014 (*1*). Over the past 20 years, neural stem cells and neural progenitor cells (NSCs/NPCs) have demonstrated the ability to self-renew and differentiate into neuronal and glial cells (*2*, *3*). Recently, NSC/NPC-based therapies have been proposed as curative treatments for neurodegenerative diseases due to the potential of these cells to improve patient brain function through neuron repair and replacement (*4*). For these reasons, the biological and physical mechanisms that influence NSC/NPC fate and behavior must be better understood.

In the adult mammalian brain, self-renewing NSCs/NPCs are found in specialized microenvironments called the neurovascular niche (NVN), located in the subventricular zone (SVZ) of the lateral ventricle and the subgranular zone (SGZ) of the dentate gyrus (*3*). In the NVN, NSCs/NPCs reside close to brain microvascular networks (BMVNs) composed of brain endothelial cells (BECs), pericytes (PCs), and astrocytes (ACs). These observations suggest that microvessels regulate NSC/NPC fate in the NVN (*5*). It is well-documented that endothelial cells act as orchestrators of NSC/NPC self-renewal and neurogenesis (*6*). In the SVZ, NPCs (type C cells) undergo multiple rounds of cell division near blood vessels before committing to neurogenesis or gliogenesis (*5*). Type C cells committed to neuron differentiation give rise to a type of neuronal precursor cell (NPreC) called neuroblasts (type A cells) (*5*). Eventually, neuroblasts leave the SVZ and migrate along blood vessels to the olfactory bulb where they further differentiate into mature neurons (*7*). Collectively, these data highlight the regulatory role of blood vessels throughout mammalian neurogenesis.

Over the past decade, in vitro models of the human NVN have been developed to elucidate the cellular interactions between NSCs/NPCs and BMVNs and expedite the discovery of new therapeutics for neurodegenerative diseases (*8*). However, cultures that use two-dimensional (2D) substrates do not properly recapitulate the cytoarchitecture of the SVZ and SGZ (*9*–*11*). Models using 3D hydrogels (*12*–*14*) or spheroids (*15*–*17*) are more effective at replicating the complex cellular interactions of NSCs/NPCs and endothelial cells. However, these models lacked certain physical stimuli, such as luminal flow in blood vessels and interstitial flow (IF) through the extracellular matrix (ECM). In the brain, IF facilitates vascular and neural cell cross-talk, metabolite and growth factor transport over long distances, and the removal of macroscopic wastes through the glymphatic system (*18*). In addition, mechanical stimuli from IF have been shown to modulate cell gene and protein expression (*19*), particularly during the formation of microvessels through angiogenesis (*20*) and vasculogenesis (*21*). Once blood vessel anastomosis is achieved, intravascular flow plays a major role in vascular characteristics. In addition to the delivery of oxygen and nutrients, blood flow provides shear stress to the vascular wall. This modifies endothelial cell gene expression and blood vessel function (*22*), including the regulation of stem cell behavior in vascular niches. Moreover, the low permeability of endothelial cells is an important feature of the blood-brain barrier (BBB), which separates the blood component from the brain tissue compartment (*23*). Reproducing these physical stimuli and cytoarchitecture in vitro will be paramount for developing physiologically accurate models of the human NVN.

Because of the ability of spatiotemporal control of both biochemical and physical variables, microfluidic devices (MFDs) have become popular systems to model the interactions between endothelial cells and NSCs/NPCs. Shin and colleagues (*24*) used their microfluidic model to show that mouse NSCs cultured with BEC-coated fluidic channels exhibited increased proliferation and self-renewal marker expression, as well as reduced neurogenesis and enhanced astrogenesis. In contrast, Sances and colleagues (*25*) showed that BEC-lined fluidic channels increased the expression of neuron makers in spinal NPCs. However, both of these studies implemented predesigned vascular channels with rectangular geometries that did not properly mimic the natural morphology of brain capillaries in vivo. In a MFD model developed by Uwamori and colleagues (*26*), neuronal and vascular networks were formed by NSC neurogenesis and brain endothelial cell angiogenesis, respectively, within a 3D fibrin-Matrigel hydrogel. Similarly, Kaushik and colleagues (*27*) created a model of the human embryonic SVZ that contained a vascular plexus region overlaid by NPCs and differentiated neuronal cells. Both studies generated self-assembled, 3D vascular networks through natural vasculogenic processes. However, these microvessels were not perfused and therefore did not experience luminal flow. Recently, both Osaki and Shin groups (*28*, *29*) cultured NSC neurospheres with perfused vascular networks of human umbilical vein endothelial cells (HUVECs) within MFDs. Both groups reported that coculturing neurospheres with HUVEC microvessels enhanced neurogenesis. However, neither study generated BMVNs akin to those observed in the NVN, which are composed of BECs, PCs, and ACs (*6*). Furthermore, no study to date has investigated the influence of the bulk flow of interstitial fluid on NSC/NPC fate. Hence, there is currently no microfluidic model that completely recapitulates the cytoarchitecture and physical stimuli of the human NVN.

Recently, our group demonstrated the benefit of the application of IF when generating self-assembled, perfused BMVNs within MFDs (*30*). Here, we have built off our previous work by introducing human induced pluripotent stem cell (iPSC)-derived NPCs and NPreCs (both as dispersed cells and neurospheres) to our BMVN model to study the influences of self-renewal and neurogenesis in vitro. Our overarching hypothesis was that both NPC self-renewal and NPreC neurogenesis would be enhanced in the presence of BMVNs, when compared to NPCs and NPreCs cultured alone. We further investigated if the application of IF during BMVN coculture contributed to the degree of self-renewal and neurogenesis. NPC survival and proliferation were measured by the number of dispersed NPCs and the physical expansion of NPC neurospheres. Neurogenesis was evaluated by the outgrowth of neurites and spontaneous calcium oscillations in NPreC neurospheres, as well as the expression of self-renewal and neuron markers in individual NPreCs. Our work highlights the regulatory role of BMVNs in the behavior of NPCs and NPreCs and demonstrates the potential of this microfluidic model to elucidate the physical and biological mechanisms of the human NVN.

## RESULTS

### NPCs were capable of self-renewal, neurogenesis, and astrogenesis in 2D cell culture

**Fig. 1.**
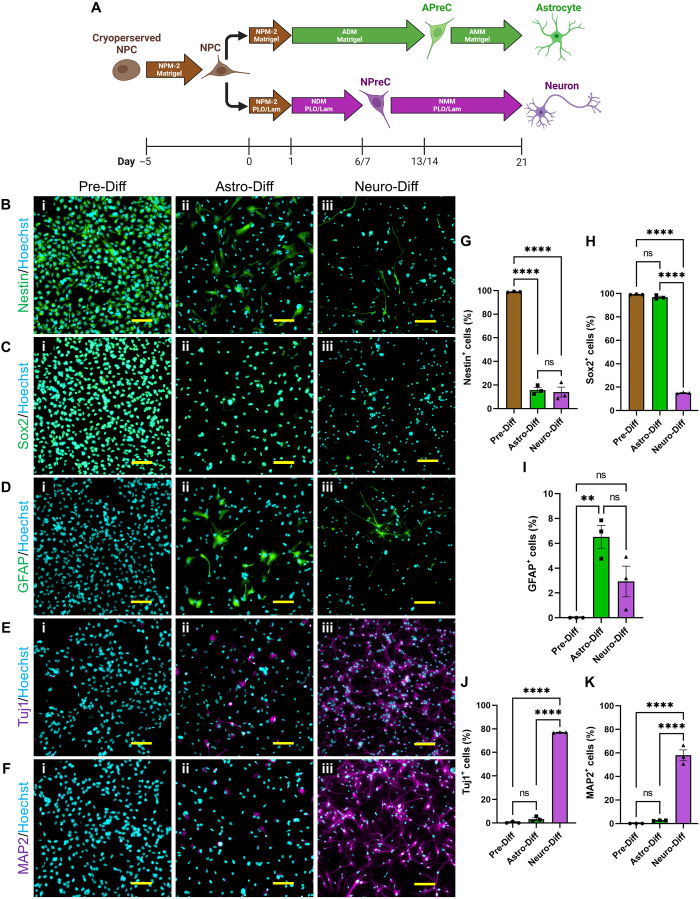
NPC differentiation and characterization. (**A**) Diagram of NPC differentiation in 2D cell culture. In the astrocyte differentiation protocol, astrocyte differentiation medium (ADM) was used to convert NPCs to astrocyte precursor cells (APreCs). Astrocyte maturation medium (AMM) was then used to convert APreCs to NPC-derived astrocytes (NPC-ACs). In the neuron differentiation protocol, neuron differentiation medium (NDM) was used to convert NPCs to NPreCs. Neuron maturation medium (NMM) was then used to convert NPreCs to NPC-derived neurons (NPC-neurons). Both differentiation cultures were terminated on day 21. Culture dishes were coated with Matrigel or poly-l-ornithine and laminin (PLO/Lam) where indicated. (**B** to **F**) Immunocytochemical analysis of NPC differentiation. Cells were immunofluorescently labeled for the neural stem cell markers, nestin (B, green) and Sox2 (C, green), the AC marker, GFAP (D, green), and the neuron markers, Tuj1 (E, purple) and MAP2 (F, purple) before any differentiation (Pre-Diff, i), as well as after astrocyte (Astro-Diff, ii) and neuron (Neuro-Diff, iii) differentiation protocols. Nuclei were labeled with Hoechst (blue). Scale bars, 100 μm. (**G** to **K**) Graphs showing the percentage of total cells that expressed nestin (G), Sox2 (H), GFAP (I), Tuj1 (J), and MAP2 (K) in Pre-Diff, Astro-Diff, and Neuro-Diff cultures. The data show mean value, error bars ± SEM, *n* = 3, one-way analysis of variance (ANOVA) with Tukey’s test; not significant (ns), *P* > 0.05; **P < 0.01; ****P < 0.0001.

Human pluripotent stem cell (XCL-1 line)–derived NPCs from Stemcell Technologies were selected for this study for several reasons. Their stem cell–like characteristics enable them to maintain a population of self-renewing cells for many generations in 2D culture. In addition, NPCs were capable of differentiating into both NPC-ACs and NPC-neurons through well-defined protocols provided by the manufacturer (Fig. 1A). To confirm the capabilities of NPCs, cells were labeled using immunocytochemical techniques for stem cell [nestin and SRY-box 2 (Sox2)], astrocyte [glial fibrillary acidic protein (GFAP)], and neuron [class III β-tubulin (Tuj1) and microtubule-associated protein 2 (MAP2)] markers before and after undergoing either astrocyte or neuron differentiation protocols. Before any differentiation, most of the NPCs were observed to express nestin (98.88 ± 0.24%; Fig. 1, Bi and G) and Sox2 (99.17 ± 0.16%; Fig. 1, Ci and H), but not GFAP (0.00 ± 0.00%; Fig. 1, Di and I), Tuj1 (0.49 ± 0.49%; Fig. 1, Ei and J), and MAP2 (0.00 ± 0.00%; Fig. 1, Fi and K). These data confirmed that NPCs cultured in Neural Progenitor Medium-2 (NPM-2) maintained a NSC-like phenotype and had not yet undergone notable neurogenesis or astrogenesis. After astrocyte differentiation, most NPC-ACs still expressed Sox2 (96.89 ± 0.93%; Fig. 1, Cii and H) but not nestin (15.77 ± 2.16%; Fig. 1, Bii and G). Compared to NPCs, NPC-ACs had increased expression of GFAP (6.52 ± 0.92%; Fig. 1, Dii and I), but no significant change in Tuj1 (3.33 ± 1.24%; Fig. 1, Eii and J) and MAP2 (2.60 ± 0.14%; Fig. 1, Fii and K) expression. Together, these results indicate that NPCs are capable of astrogenesis. However, 21 days of astrocyte differentiation culture was not sufficient time to produce a substantial population of GFAP^+^ NPC-ACs. With that said, the protocol from Stemcell Technologies recommended culturing astrocyte precursor cells (APreCs) in astrocyte maturation medium (AMM) for at least two additional passages to promote ubiquitous GFAP expression. However, we did not attempt further astrocyte differentiation due to the time requirement. After neuron differentiation, most NPC-neurons had no visible expression of nestin (14.07 ± 4.22%; Fig. 1, Biii and G) and Sox2 (15.09 ± 0.29%; Fig. 1, Ciii and H). A small population of NPC-neurons expressed GFAP (2.93 ± 1.23%; Fig. 1, Diii and I), but not enough to be statistically significant from NPCs. After 14 days in neuron maturation medium (NMM), most NPC-neurons expressed Tuj1 (77.03 ± 0.21%; Fig. 1, Eiii and J) and MAP2 (57.93 ± 4.61%; Fig. 1, Fiii and K). These data showcased the ability of NPCs to undergo neurogenesis and produce neurons with mature markers. Collectively, these results demonstrated that XCL-1–derived NPCs had self-renewing characteristics as well as the capability to differentiate into both NPC-ACs and NPC-neurons in 2D cell culture under defined medium conditions.

### NPCs did not survive solo-culture in MFDs

**Fig. 2.**
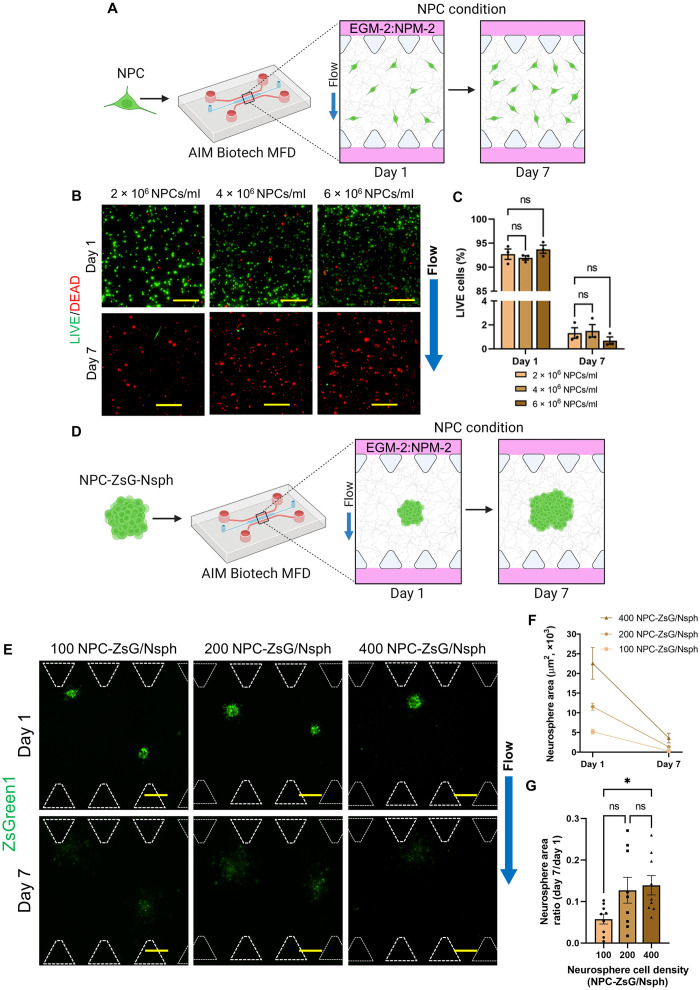
Dispersed NPCs and NPC-ZsG neurospheres (NPC-ZsG-Nsphs) in MFD culture. (**A**) Illustration of dispersed NPC solo-culture in MFDs. NPCs were resuspended in fibrin gels (NPC condition) at three different cell densities within MFDs. Samples were cultured for 1 week in EGM-2:NPM-2 under flow conditions. (**B**) LIVE (green)/DEAD (red) MIPs of NPCs on day 1 and day 7. NPCs were resuspended in fibrin at 2 × 10^6^, 4 × 10^6^, or 6 × 10^6^ NPCs/ml. (**C**) Graph of the percentage of LIVE NPCs cultured at different cell densities. The data show mean value, error bars ± SEM, *n* = 3. (**D**) Illustration of NPC-ZsG-Nsph solo-culture in MFDs. NPCs-ZsG were aggregated into neurospheres of three different cell densities and resuspended in fibrin gels (NPC condition) within MFDs. Cells were cultured for 1 week in EGM-2:NPM-2 under flow conditions. (**E**) Fluorescence MIPs of NPC-ZsG-Nsphs (ZsGreen1, green) with cell densities of 100, 200, and 400 NPCs-ZsG per neurosphere (NPC-ZsG per Nsph) cultured in MFDs on day 1 and day 7. White dotted lines outline microposts. (**F** and **G**) Graphs showing the change in neurosphere area from day 1 to day 7 (F) and the day 7/day 1 neurosphere area ratio (G) for all neurosphere densities. *N* = 9 neurospheres were measured from n = 3 MFDs. Scale bars, 200 μm (B) and (E). Blue arrows indicate the direction of IF. (C), (F), and (G) The data show mean value, error bars ± SEM, two-way ANOVA (C) and Welch’s ANOVA (G) with Dunnett’s test; ns, *P* > 0.05; *P < 0.05.

Once it was confirmed that 2D NPCs were capable of self-renewal and neuron differentiation, we wanted to determine whether NPCs exhibited these traits in a 3D microenvironment. MFDs from AIM Biotech were selected as a platform for this work due their implementation in a previous publication in which our group developed BMVNs in vitro (*30*). Before NPCs were cocultured with other cell types in MFDs, they were cultured alone (Fig. 2A) to characterize their behavior. First, NPCs were resuspended in fibrin gels at different cell densities (2 × 10^6^, 4 × 10^6^, and 6 × 10^6^ cells/ml) to determine which NPC population promoted the most cell survival. Using LIVE/DEAD staining, we confirmed that a majority of NPCs in all conditions were viable on day 1. However, nearly all NPCs died after 1 week in culture, regardless of initial cell density (Fig. 2B). LIVE/DEAD staining quantification showed that NPCs cultured at 2 × 10^6^, 4 × 10^6^, and 6 × 10^6^ cells/ml had LIVE cell percentages of 92.71 ± 1.07, 91.95 ± 0.45, and 93.73 ± 0.84%, respectively, on day 1, and 1.32 ± 0.44, 1.50 ± 0.52, and 0.69 ± 0.30%, respectively, on day 7 (Fig. 2C). These data confirmed that NPC viability was not improved by increasing the cell density, indicating that NPCs did not commit apoptosis due to a deficiency of homotypic cellular interactions. Moving forward, we cultured NPCs (2 × 10^6^ cells/ml) in fibrin gels containing Matrigel to determine whether the contents of the ECM (laminin, collagen IV, entactin, and perlecan) would improve NPC viability (fig. S1A). However, the mean percentage of LIVE NPCs cultured in fibrin and fibrin-Matrigel decreased from 91.70 ± 3.85 and 94.92 ± 2.80%, respectively, on day 1 to 3.87 ± 1.28% and 3.44 ± 1.04%, respectively, on day 7 (fig. S1C). These results indicated that the addition of Matrigel to the fibrin gel did not improve NPC viability in MFD culture. To determine whether the default MFD culture conditions for NPC expansion [Endothelial Cell Growth Medium 2 (EGM-2):NPM-2 with the application of IF] were the primary causes of cell death, we also cultured NPCs in just NPM-2 under flow conditions and in EGM-2:NPM-2 under static conditions. However, similar to the results of the previous experiments, a majority of NPCs died after 5 days in all cultures (fig. S1B). The percentage of LIVE NPCs cultured in EGM-2:NPM-2 under flow conditions decreased from 91.45 ± 2.64% on day 1 to 1.97 ± 0.50% on day 5 (fig. S1D). Similarly, the percentage of LIVE NPCs cultured in NPM-2 under flow conditions decreased from 92.90 ± 1.47 on day 1 to 3.88 ± 0.83% on day 5 (fig. S1D). Last, NPCs cultured in EGM-2:NPM-2 under static conditions had 89.38 ± 1.03% LIVE cells on day 1 and 3.80 ± 1.17% on day 5 (fig. S1D). These data signified that neither the presence nor absence of EGM-2 or IF improved NPC viability within MFDs. Collectively, the results concluded that the solo-culture conditions within MFDs were not conducive for NPC survival and proliferation.

After it was confirmed that dispersed NPCs did not survive for extended culture periods in MFDs, we postulated that cell survival would be improved if NPCs were aggregated into neurospheres (NPC-Nsphs). NPC-Nsphs with densities of 100, 200, and 400 cells per Nsph were formed in microwell plates (fig. S2B) and produced neurosphere populations with average diameters of 65.86 ± 1.00, 75.55 ± 1.46, and 100.70 ± 1.22 μm, respectively (fig. S2C). To determine whether increasing neurosphere cell density would improve neurosphere expansion, NPC-ZsG-Nsphs composed of 100, 200, and 400 NPCs-ZsG per Nsph were cultured alone in MFDs (Fig. 2D). On day 1, all neurospheres had strong ZsGreen1 signals and were observed extending cellular processes into fibrin gels. However, the ZsGreen1 signal for all neurospheres became barely visible after 1 week in MFDs (Fig. 2E). Dim ZsGreen1 signals observed outside of the initial neurosphere border indicated that NPCs-ZsG had migrated into the fibrin gel before committing apoptosis. The ZsGreen1 proteins of apoptotic cells likely degraded and removed from the fibrin ECM by the bulk flow of interstitial fluid. For these reasons, the change in neurosphere area from day 1 to day 7 was negative in all cultures (Fig. 2F). The neurosphere area ratio was found to be 0.058 ± 0.012, 0.127 ± 0.031, and 0.139 ± 0.023 for neurospheres composed of 100, 200, and 400 NPC-ZsG per Nsph, respectively (Fig. 2G). The mean neurosphere area ratio was significantly larger for neurospheres composed of 400 NPC-ZsG per Nsph when compared to those composed of 100 NPC-ZsG per Nsph. However, this enhancement was immaterial since neurospheres in both conditions had been significantly reduced from their original sizes. These data proved that increasing neurosphere cell density did not improve NPC-ZsG-Nsph expansion in solo-culture and confirmed our conclusion that culture conditions within MFDs were simply not conducive for the survival and proliferation of NPCs.

### NPC survival was enhanced by coculture with BMVNs in MFDs

After it was confirmed that NPCs could not be maintained in MFD solo-culture, both as dispersed cells and neurospheres, we hypothesized that cell survival would be enhanced when NPCs were cocultured with BMVNs. Previously, we developed a protocol (fig. S3A) to consistently generate BMVNs within MFDs from AIM Biotech (*30*). BMVNs were composed of primary BECs-tdT, PCs, and ACs which expressed CD31 (fig. S3C), NG-2, and GFAP, respectively (fig. S3B). We previously demonstrated that MFDs were capable of generating a maximum IF velocity of 5.73 μm/s across the fibrin gel under flow conditions (hydrostatic pressure difference of 1.5 mmH_2_O) (*30*). The application of IF enhanced BMVN interconnectivity and perfusion, as confirmed by flowing microspheres through the open lumen of microvessels (fig. S3D). In our previous publication, we calculated that the average shear stress experienced by the vessel wall of microvessels under flow conditions was 0.394 dyn/cm^2^ (*30*). After 1 week, fully developed BMVNs retained 70-kDa dextran within their lumen (fig. S3E), demonstrating the low permeability of brain microvessels.

**Fig. 3.**
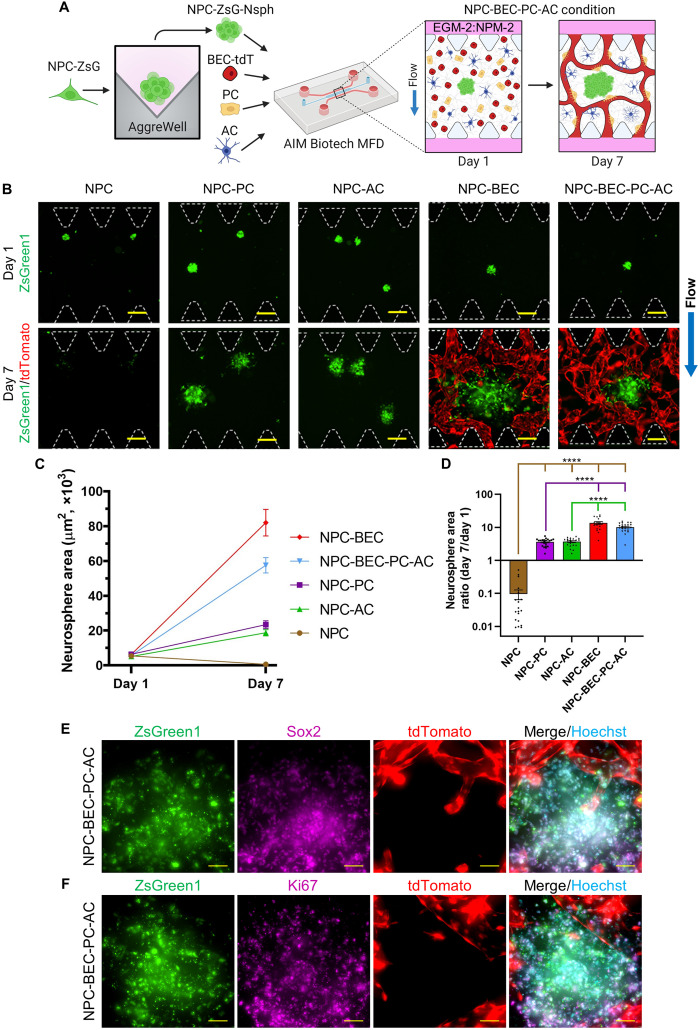
NPC neurosphere (NPC-Nsph) expansion in MFDs. (**A**) Illustration of the culture protocol used to investigate NPC-Nsph expansion. Briefly, NPCs expressing ZsGreen1 (NPCs-ZsG) were aggregated into neurospheres (NPC-ZsG-Nsphs, 200 NPC-ZsG per Nsph) and cultured alone (NPC condition), with PCs (NPC-PC condition), with ACs (NPC-AC condition), with BECs-tdT (NPC-BEC condition), and with all three cell types (NPC-BEC-PC-AC condition) in fibrin gels within MFDs. Samples were cultured for 1 week in EGM-2:NPM-2 under flow conditions. Illustration only shows NPC-BEC-PC-AC condition. (**B**) Fluorescence MIPs of NPC-ZsG-Nsphs (ZsGreen1, green) cultured in NPC, NPC-PC, NPC-AC, NPC-BEC, and NPC-BEC-PC-AC conditions on day 1 and day 7. BECs-tdT microvessels (tdTomato, red) were only imaged on day 7. PCs and ACs not shown. White dotted lines outline microposts. Blue arrow indicates the direction of IF for all conditions. (**C** and **D**) Graphs showing the change in neurosphere area from day 1 to day 7 (C) and the day 7/day 1 neurosphere area ratio (D) for all conditions. *N* ≥ 18 neurospheres were measured from *n* = 3 MFDs, the data show mean value, error bars ± SEM, Welch’s ANOVA with Dunnett’s test, the absence of significance line indicates *P* > 0.05, ****P < 0.0001. Brown, purple, and green significance lines show statistical comparisons made for NPC, NPC-PC, and NPC-AC conditions, respectively. (**E** and **F**) Fluorescence MIPs of NPC-ZsG-Nsphs (ZsGreen1, green) expressing Sox2 (E, purple) and Ki67 (F, purple) with microvessels (tdTomato, red) in NPC-BEC-PC-AC conditions on day 7. Nuclei were labeled with Hoechst (blue). Scale bars, indicate 200 μm (B) and 50 μm (E) and (F).

In this work, we modified our protocol to develop BMVNs capable of supporting NPC survival and proliferation. NPCs-ZsG were aggregated into neurospheres and resuspended in fibrin gels within BECs-tdT, PCs, and ACs within MFDs (Fig. 3A). A cell density of 200 cells per Nsph was chosen to produce NPC-ZsG-Nsphs that would expand in the presence of BMVNs without occupying the entire width of the hydrogel channel. Anticipating that NPC-ZsG-Nsphs would expand in NPC-BEC-PC-AC conditions, we also cultured neurospheres in NPC, NPC-PC, NPC-AC, and NPC-BEC conditions to identify which brain cell type contributed the most to NPC-ZsG-Nsph growth. After 1 day in MFD culture, NPC-ZsG-Nsphs were observed extending small cellular processes into fibrin gels in all conditions (Fig. 3B). After 1 week, neurospheres with strong ZsGreen1 signals were observed in all cultures except in NPC conditions (Fig. 3B). NPC-ZsG-Nsphs cultured alone experienced regression similar to the observations shown in Fig. 2E. In contrast, NPC-ZsG-Nsphs grown in all the other coculture conditions increased in size over the course of 1 week (Fig. 3B). With that said, the final size of NPC-ZsG-Nsphs cultured in NPC-BEC and NPC-BEC-PC-AC conditions appeared larger than that of neurospheres cultured in NPC-PC and NPC-AC conditions. Because of the presence of endothelial cells, microvessels were only observed in NPC-BEC, NPC-BEC-PC-AC, and NPC-HUVEC-PC-AC samples (Fig. 3B and fig. S9A). These microvessels formed networks between the two fluidic channels and were observed circumventing NPC-ZsG-Nsphs in the hydrogel channel.

After measuring the neurosphere area for NPC-ZsG-Nsphs in all conditions, we found that only the NPC condition produced a negative trend from day 1 to day 7 (Fig. 3C). To compare neurosphere growth between conditions, we calculated the mean day 7/day 1 neurosphere area ratio for NPC-ZsG-Nsphs in NPC (0.10 ± 0.03), NPC-PC (3.61 ± 0.20), NPC-AC (3.69 ± 0.22), NPC-BEC (13.53 ± 1.30), and NPC-BEC-PC-AC (10.15 ± 0.62) conditions (Fig. 3D). Compared to neurospheres cultured alone, NPC-ZsG-Nsphs cultured with PCs, ACs, BECs-tdT, and all three cell types exhibited significantly enhanced neurosphere expansion. While no statistical difference was found between the mean neurosphere area ratio for NPC-ZsG-Nsphs cultured with PCs and ACs, the area ratio means in NPC-BEC and NPC-BEC-PC-AC conditions were found to be significantly larger than those in both NPC-PC and NPC-AC conditions. No statistical difference was found between the neurosphere area ratio means for NPC-BEC and NPC-BEC-PC-AC conditions (Fig. 3D), as well as for NPC-BEC-PC-AC and NPC-HUVEC-PC-AC conditions (fig. S9B). These data suggested that neurosphere expansion was primarily due to the presence of BECs-tdT or HUVECs-tdT in the fibrin gel. This was likely due to soluble neurotrophic factors that have previously been reported to be secreted by endothelial cells (*6*, *8*). However, it should be noted that BECs-tdT were seeded at a higher cell density (6 × 10^6^ cells/ml) than PCs and ACs (2 × 10^6^ cells/ml). Therefore, the enhanced neurosphere expansion could be attributed to the larger population of supporting cells. Nonetheless, these data demonstrated that the vascular component of BMVNs is important for NPC-ZsG-Nsph survival and growth in quad-cultures with all cell types present.

To confirm that NPCs-ZsG in NPC-BEC-PC-AC conditions were self-renewing and proliferating after 1 week in culture, we stained NPC-ZsG-Nsphs with Sox2 (Fig. 3E) and Ki67 (Fig. 3F), respectively. The presence of Sox2^+^ NPCs-ZsG confirmed that a substantial population of cells retained their stem cell identity in coculture with BMVNs. In addition, the presence of Ki67^+^ NPCs-ZsG confirmed that neurosphere expansion was a product of NPC-ZsG proliferation and not simply NPC-ZsG migration into the fibrin gel. Together, these results justify the culture of NPCs with BMVNs to produce dividing, self-renewing neural cells in an in vitro microenvironment that resembles the human NVN.

**Fig. 4.**
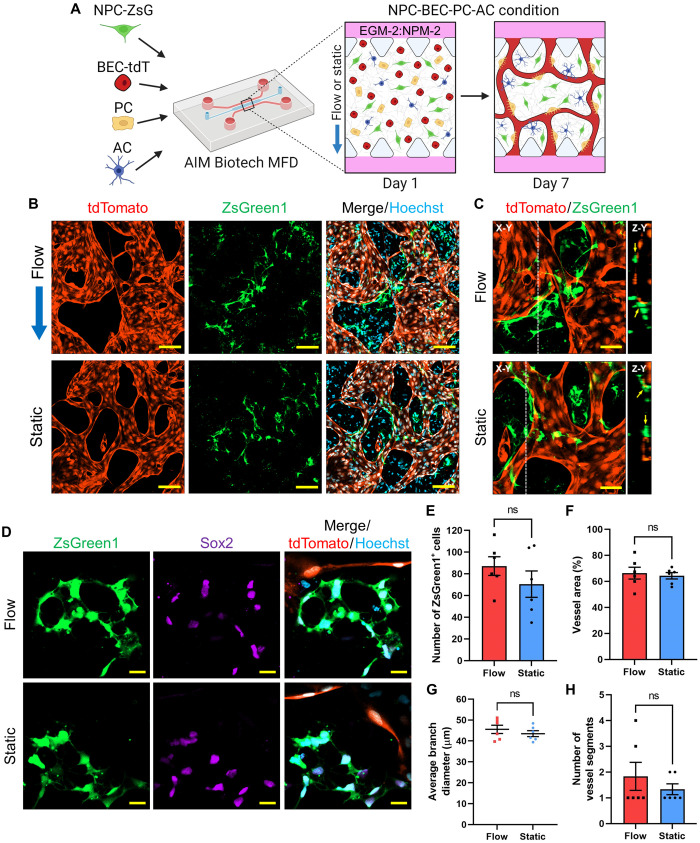
Expansion of dispersed NPCs with BMVNs in MFDs. (**A**) Illustration of the culture protocol used to investigate the effect of IF on expansion of dispersed NPCs with BMVNs in MFDs. Briefly, NPCs expressing ZsGreen1 (NPCs-ZsG) were cultured with BECs-tdT, PCs, and ACs (NPC-BEC-PC-AC condition) in fibrin gels within MFDs. Samples were cultured for 1 week in EGM-2:NPM-2 under flow or static conditions. (**B**) Fluorescence confocal MIPs of NPC-BEC-PC-AC cultures under flow and static conditions on day 7. NPCs-ZsG and BECs-tdT were identified with ZsGreen1 (green) and tdTomato (red), respectively. Nuclei were labeled with Hoechst (blue). Blue arrow indicates the direction of IF in flow condition. (**C**) Fluorescence confocal MIPs (X-Y) of NPCs-ZsG (ZsGreen1, green) making direct contact with hollow microvessels (tdTomato, red) under flow and static conditions. White dotted lines indicate Z-Y cross sections. (**D**) Fluorescence confocal images of NPCs-ZsG (ZsGreen1, green) expressing Sox2 (purple) near microvessels (tdTomato, red) under flow and static conditions. Nuclei were labeled with Hoechst (blue). Scale bars, indicate 100 μm (B), 50 μm (C), and 20 μm (D). (**E** to **H**) Graphs showing the number of ZsGreen1^+^ cells within a given area (E), as well as the vessel area (F), average branch diameter (G), and the number of vessel segments (H) for samples in flow and static conditions on day 7. The data show mean value, error bars ± SEM, *n* = 6, Welch’s *t* test; ns, *P* > 0.05.

After we confirmed that NPC-ZsG-Nsph expansion was enhanced in the presence of microvessels, we postulated that the survival of dispersed NPCs-ZsG would also be increased when cocultured with BMVNs. However, knowing that dispersed NPCs did not survive extended culture periods in MFDs (Fig. 2, B and C, and fig. S1, A to D), we did not culture NPCs-ZsG alone as a control. Instead, we cocultured dispersed NPCs-ZsG with BECs-tdT, PC, and ACs within MFDs. Given the importance of IF for BMVN formation and function, as demonstrated in our previous study (*30*), we hypothesized that the application of IF would enhance NPC-ZsG proliferation in quad-culture. To test this, samples were prepared in NPC-BEC-PC-AC conditions and then cultured under either flow or static conditions (Fig. 4A). After 1 week, dispersed NPCs-ZsG were observed in the extracellular spaces between BMVNs formed under both conditions (Fig. 4B). In both flow and static samples, NPCs-ZsG were observed directly contacting brain microvessels with open lumen (Fig. 4C). On day 7, the mean number of ZsGreen1^+^ cells (per area) was 87.17 ± 8.57 and 70.50 ± 12.15 in flow and static conditions, respectively (Fig. 4E). Despite the slight increase in the mean number of NPCs-ZsG present in flow samples, no significant difference was found from that of static samples. This indicated that IF had no notable effect on the number of NPCs-ZsG when cocultured with BMVNs. Moreover, immunostaining revealed that NPCs-ZsG found in both culture conditions expressed Sox2 (Fig. 4D), confirming that NPCs-ZsG had maintained their stem cell identify after a week MFD culture. We did not find evidence of NPCs-ZsG lacking Sox2 expression in any flow or static samples at this time point. The presence of NPM-2 in the culture medium was likely sufficient to maintain Sox2 expression in NPCs-ZsG. For this reason, no statistical analysis was performed to compare the percentage of Sox2^+^ NPCs-ZsG between flow and static cultures. With regard to microvessel morphology, no statistical difference was found between the mean vessel area (Fig. 4F), average branch diameter (Fig. 4G), and the number of vessel segments (Fig. 4H) for microvessels in both conditions. Together, these data showed that IF had no effect on microvessel morphology. We found these results to be unexpected, given that our previous study demonstrated the benefit of interstitial fluid flow for BMVN formation in MFDs (*30*). However, samples from that study were cultured in only EGM-2, while all experiments in this section used EGM-2:NPM-2. It is possible that the growth factors present in NPM-2 were beneficial for vascular network formation despite the lack of IF in static samples.

Although our primary objective was to determine the effect of BMVNs on NPC-ZsG survival and proliferation, we were also interested in the reciprocal influence of NPCs on vascular morphogenesis. To test this in MFDs, we stimulated BECs-tdT to produce angiogenic sprouts into fibrin matrices without cells, with PCs, with ACs, and with NPCs to compare the angiogenic effect of each support cell (fig. S4A). After 1 week, the number of sprouts observed in BEC, BEC-PC, BEC-AC, and BEC-NPC conditions was 10.00 ± 2.52, 23.00 ± 2.65, 6.00 ± 0.58, 4.00 ± 1.00, respectively (fig. S4B), and the average sprout length was 241.07 ± 11.00, 634.79 ± 15.03, 316.98 ± 25.71, and 160.15 ± 18.58 μm, respectively (fig. S4C). Both sprout count and average sprout length were significantly enhanced in the BEC-PC condition compared to the BEC condition, highlighting the supportive role of PCs during angiogenesis. Both the mean sprout count and average sprout length were significantly reduced for BECs-tdT cultured with NPCs when compared to BECs-tdT cultured alone. These data suggest an antagonistic effect of NPCs on angiogenesis. However, given the substantial amount of cell death experienced by dispersed NPCs in MFD culture (Fig. 2, B and C, and fig. S1, A to D), it is more likely that this was the cause of the reduction in angiogenic sprout count and length. Despite the negative effect of NPCs on BEC angiogenesis, we wanted to confirm whether dispersed NPCs had any pro-vasculogenic effects. Since NPCs-ZsG proliferated when cocultured with BMVNs (Fig. 4B), we postulated that the survival of dispersed NPCs would be improved when BECs-tdT were cocultured with them in the fibrin gels rather than in the fluidic channels. Therefore, to determine the influence of NPCs on vasculogenesis, we stimulated dispersed BECs-tdT to form vascular networks in BEC, BEC-PC, BEC-AC, and BEC-NPC conditions. After 1 week, BECs-tdT cultured with PCs or ACs formed large microvessels with interconnected networks (fig. S4D). In contrast, microvessels in BEC and BEC-NPC conditions were visibility smaller and segmented. No significant difference was found for the average branch diameter (fig. S4F) and the number of vessel segments (fig. S4G) between BECs-tdT cultured alone and BECs-tdT cocultured with NPCs. The mean vessel area was found be to significantly larger in BEC-NPC conditions than in BEC conditions but only slightly (fig. S4E). Coculture with either PCs or ACs significantly enhanced vessel area (fig. S4E) and average branch diameter (fig. S4F), as well as reduced the number of vessel segments (fig. S4G) when compared to BECs-tdT cultured alone. In summary, these data demonstrated that NPCs did not have any notable pro-angiogenic or pro-vasculogenic effects. However, the development of fully formed BMVNs in NPC-BEC-PC-AC conditions (Fig. 4B) demonstrated that the supportive role of PCs and ACs was substantial enough to ensure proper vascular network formation in quad-culture with NPCs-ZsG.

### NPreC neurogenesis was enhanced by coculture with BMVNs in MFDs

We previously showed that NPCs experienced apoptosis when cultured alone in fibrin matrices within MFDs (Fig. 2B). We speculated that the initial differentiation of NPCs to NPreCs in 2D culture (Fig. 1A) would improve the viability of cells in MFDs. To test this, we cultured dispersed NPreCs with EGM-2:NMM in MFDs for 14 days (fig. S5A). Although the majority of NPreCs were alive at the start of MFD culture, most NPreCs were dead after 2 weeks (fig. S5B). The percentage of LIVE NPCs was 96.51 ± 1.19, 46.93 ± 1.53, and 17.68 ± 3.59% on day 1, day 7, and day 14, respectively (fig. S5C), showing that the viability of dispersed NPreCs gradually decreased over time. These results were similar to our observations of dispersed NPCs in MFDs (Fig. 2, B and C), although small populations of living NPreCs still remained after 2 weeks. Surviving NPreCs were usually found in cell clusters on day 14 (fig. S5, D and E). Immunohistochemistry identified Sox2^+^/MAP2^−^, Sox2^+^/MAP2^+^, and Sox2^−^/MAP2^+^ cells (fig. S5D), showcasing the differentiation progression from self-renewing NPreCs to mature NPC-neurons in vitro. No GFAP^+^ NPreCs were identified in any samples (fig. S5E), confirming that no astrogenesis occurred during these specific culture conditions. Together, these results demonstrated the potential of NPreCs to undergo neurogenesis in MFD culture, despite the abundance of cell death.

**Fig. 5.**
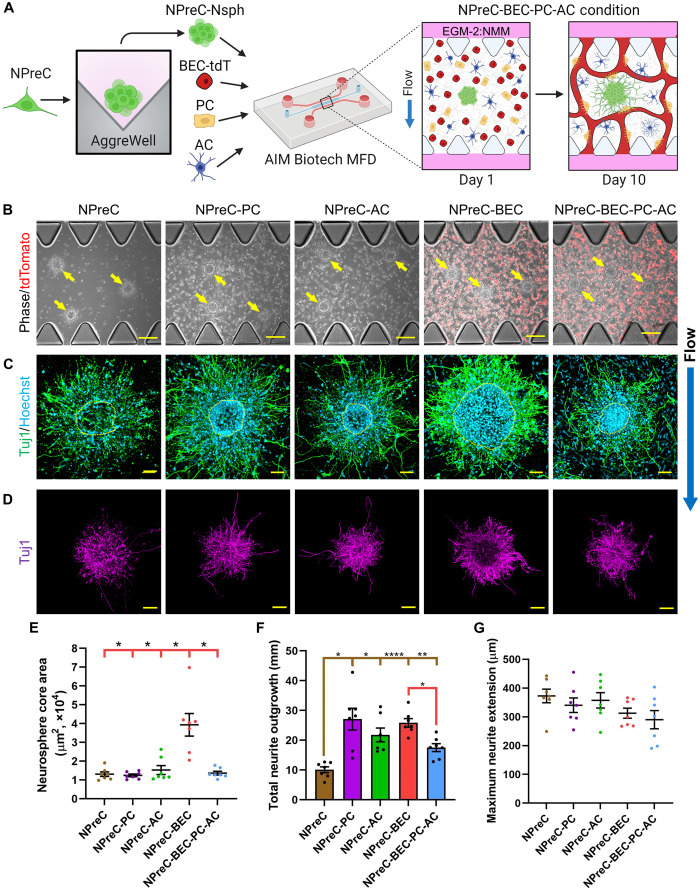
NPreC neurosphere (NPreC-Nsph) neurogenesis in MFDs. (**A**) Illustration of the culture protocol used to investigate NPreC-Nsph neurogenesis. Briefly, NPreC-Nsphs were cultured alone (NPreC condition), with PCs (NPreC-PC condition), with ACs (NPreC-AC condition), with BECs-tdT (NPreC-BEC condition), and with all three cell types (NPreC-BEC-PC-AC condition) in fibrin gels within MFDs. Samples were cultured for 10 days in EGM-2:NMM under flow conditions. Illustration only shows NPreC-BEC-PC-AC condition. (**B**) Phase/fluorescence images of NPreC-Nsphs in all conditions on day 1. Yellow arrows identify NPreC-Nsphs. BECs-tdT expressed tdTomato (red). (**C**) Fluorescence confocal MIPs of NPreC-Nsphs in all conditions on day 10. NPC-neurons were labeled with Tuj1 (green) and Hoechst (blue). BECs-tdT, PCs, and ACs are not shown. Yellow dotted lines outline neurosphere cores. (**D**) Large fluorescence confocal MIPs of NPreC-Nsphs (Tuj1, purple) in all conditions. Background signals were removed to highlight neurites. Scale bars, 200 μm (B), 50 μm (C), and 100 μm (D). Blue arrow indicates direction of IF. (**E** to **G**) Graphs showing the neurosphere core area (E), total neurite outgrowth (F), and maximum neurite extension (G) measured for NPreC-Nsphs from all conditions on day 10. The data show mean value, error bars ± SEM, data from *N* = 7 neurospheres from *n* = 3 MFDs, Welch’s ANOVA with Dunnett’s test, the absence of significance line indicates *P* > 0.05, *P < 0.05, **P < 0.01, ****P < 0.0001. Brown and red significance lines show statistical comparisons made for NPreC and NPreC-BEC conditions, respectively.

After it was confirmed that most dispersed NPreCs died after extended periods of solo-culture in MFDs, we choose to aggregate NPreCs into neurospheres to further study NPreC survival and neurogenesis. NPreC-Nsphs with densities of 100, 200, and 400 cells per Nsph were formed in microwell plates (fig. S2B) and produced neurosphere populations with average diameters of 65.58 ± 0.35, 76.72 ± 0.52, and 103.94 ± 0.76 μm, respectively (fig. S2C). Because of our previous observations with NPC-ZsG-Nsphs (Fig. 2, E to G), we choose not to culture NPreC-Nsphs alone in MFDs to determine the effect of neurosphere cell density on cell survival. Instead, NPreC-Nsphs were resuspended in fibrin gels within BECs-tdT, PCs, and ACs within MFDs (Fig. 5A). Because of the use of EGM-2:NMM in neurogenesis experiments, we did not anticipate a substantial increase in neurosphere area. Therefore, a cell density of 400 cells per Nsph was selected to produce NPreC-Nsphs which maximized the chance of observable neuritogenesis. Predicting that NPreC-Nsphs would undergo neurogenesis in NPreC-BEC-PC-AC conditions, we also cultured neurospheres in NPreC, NPreC-PC, NPreC-AC, and NPreC-BEC conditions to identify which cell type contributed the most to neuron differentiation. On day 1 of MFD culture, NPreC-Nsphs were observed extending cellular processes within fibrin gels in all conditions, indicating the presence of healthy NPreCs (Fig. 5B). After allowing neurospheres to undergo neurogenesis for 10 days, Hoechst and Tuj1 staining revealed that most NPreC-Nsphs had a dense core of NPC-neurons surrounded by satellite NPC-neurons that had migrated into the surrounding ECM (Fig. 5C). For all NPreC-Nsphs, we manually outlined the perimeter of the dense core and considered the region within this perimeter to be the neurosphere core area. The mean neurosphere core area of NPreC-Nsphs cultured under NPreC, NPreC-PC, NPreC-AC, NPreC-BEC, and NPreC-BEC-PC-AC conditions was found to be 13,090 ± 1199, 12,444 ± 715, 15,284 ± 2373, 39,382 ± 6000, and 13,620 ± 997 μm^2^, respectively (Fig. 5E). The mean neurosphere core area was found to be significantly larger in NPreC-BEC conditions when compared to all other culture conditions. This implied that coculture with BECs-tdT promoted NPreCs to proliferate considerably and extend the border of the neurosphere core. No statistical significance was found between the mean core areas of neurospheres in any other conditions. This was because we defined the neurosphere core area to be the region within the visible border of the main neurosphere body. However, this parameter did not describe the composition of the core of each NPreC-Nsph. Although the borders of NPreC-Nsphs in solo-culture were easy to identify and outline, the densities of Tuj1^+^ nuclei in neurosphere cores were visibly less than in all other coculture conditions (Fig. 5C). These observations implied that NPC-neurons in solo-culture migrated into the fibrin gel and did not proliferate enough to replenish the cell population in the neurosphere core. In contrast, neurosphere cores were densely packed with NPC-neurons in NPreC-BEC and NPreC-BEC-PC-AC conditions on day 10 (Fig. 5C). In NPreC-PC and NPreC-AC conditions, neurosphere cores typically had well defined borders with dense peripheries but relatively sparse centers (Fig. 5C). Together, these results indicated that BECs-tdT were likely the primary reason for NPreC proliferation and neurogenesis in neurospheres. The notably smaller neurosphere core area observed in NPreC-BEC-PC-AC conditions when compared to NPreC-BEC conditions (Fig. 5E) was probably due to the higher total cell number in quad-culture conditions. We believe that the coculture of BECs-tdT, PCs, ACs, and NPreCs resulted in higher cellular competition for nutrients and growth factors in the hydrogel channel (*31*). However, these speculations were based on visual observations alone.

As previously stated, neurospheres in all culture conditions extended Tuj1^+^ neurites into the fibrin hydrogel after 10 days in MFDs (Fig. 5, C and D). However, closer inspection of neurites revealed speckled Tuj1 signals near neurospheres, particularly for NPreC-Nsphs in NPreC conditions (Fig. 5C). Presumably, these fragmented signals indicated regions where neurites had extended and regressed either due to normal neurogenic processes or cell death. To prevent these fragmented Tuj1 signals from compromising the measurement of neurite characteristics, they were removed during image analysis. Afterward, we found the mean total neurite outgrowth to be 10.10 ± 0.98, 27.09 ± 3.65, 21.80 ± 2.31, 25.84 ± 1.44, and 17.52 ± 1.32 mm for neurospheres in NPreC, NPreC-PC, NPreC-AC, NPreC-BEC, and NPreC-BEC-PC-AC conditions, respectively (Fig. 5F). When compared to NPreC-Nsphs cultured alone, total neurite outgrowth was enhanced in all other coculture conditions. These data demonstrated the neurotrophic effect of BECs-tdT, PCs, and ACs, as the addition of any brain cell type, enhanced the total neurite length of NPreC-Nsphs. The total neurite outgrowth was found to be significantly higher in NPreC-BEC conditions when compared to NPreC-BEC-PC-AC conditions (Fig. 5F). However, as previously stated, this was probably due to the larger total cell number present in quad-cultures, which occupy more space in the ECM and compete with NPreC-Nsphs for nutrition. Nonetheless, neurospheres in all culture conditions had NPC-neurons with several neurites that extended further into the fibrin gel than the majority of other neuronal processes (Fig. 5D). We found the mean maximum neurite extension for NPreC-Nsphs in NPreC, NPreC-PC, NPreC-AC, NPreC-BEC, and NPreC-BEC-PC-AC conditions to be 372.68 ± 23.83, 340.41 ± 25.18, 357.37 ± 26.69, 312.79 ± 17.38, and 290.30 ± 31.77 μm, respectively (Fig. 5G). No significant difference was found between the maximum neurite extension means of any two culture conditions. This indicated that NPreC-Nsphs in solo-culture were able to produce neurites with maximum lengths comparable to those of NPreC-Nsphs in all coculture conditions, despite their sparse neurosphere core and diminished total neurite outgrowth. We previously observed that dispersed NPC-neurons were able to survive in small clusters for up to 2 weeks in MFDs (fig. S5, D and E). Therefore, the aggregation of NPreCs into neurospheres probably facilitated significant neurite extension for the surviving NPC-neurons in solo-culture. However, it is likely that these long neurites would eventually regress if the culture period was extended due to lack of any supporting cells in NPreC conditions.

**Fig. 6.**
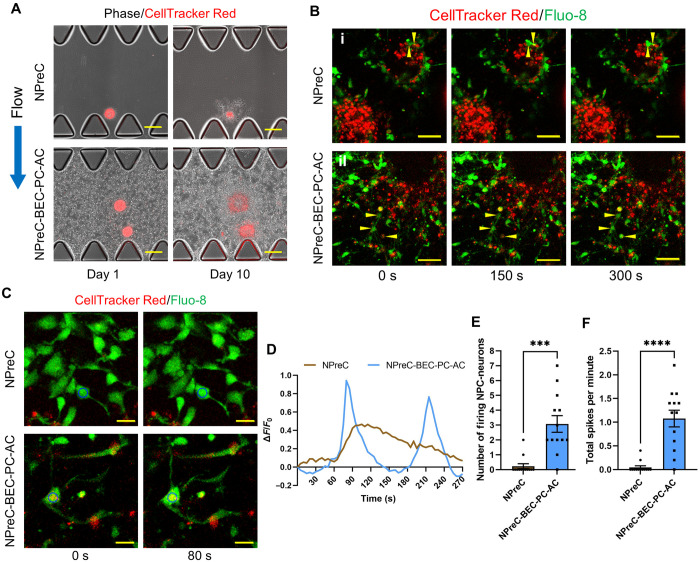
Calcium oscillations of NPreC-Nsphs in MFDs. (**A**) Phase/fluorescence images of NPreC-Nsphs (400 NPreCs per Nsph) with CellTracker Red (red) cultured alone (NPreC condition) and with BECs, PCs, and ACs (NPreC-BEC-PC-AC condition) in MFDs on day 1 and day 10. Blue arrow indicates the direction of IF in both conditions. (**B**) Fluorescence time-lapse confocal MIPs of NPreC-Nsph (CellTracker Red, red) slices in NPreC (i) and NPreC-BEC-PC-AC (ii) conditions on day 10. Samples were stained with Fluo-8 (green) to observe intracellular calcium fluctuations. Yellow triangles identify NPC-neurons experiencing calcium oscillations during imaging. (**C**) Fluorescence time-lapse confocal images of individual NPC-neurons (CellTracker Red, red) stained with Fluo-8 (green) in both culture conditions. Blue circles identify the cell body of NPC-neurons used for Fluo-8 intensity measurement shown in the next graph (D). Scale bars, 200 μm (A), 50 μm (B), and 20 μm (C). (**D**) Graph of the change in Fluo-8 fluorescence intensity relative to the initial value (∆*F*/*F*_0_) of the NPC-neurons identified in the previous images (C). (**E** and **F**) Graphs of the number of firing NPC-neurons (E) and total spike count per minute (F) measured per neurosphere slice in NPreC and NPreC-BEC-PC-AC conditions. The data show mean value, error bars ± SEM, data collected from *N* = 13 neurosphere slices from *n* = 3 MFDs, Welch’s *t* test, ***P < 0.001, ****P < 0.0001.

After we confirmed that NPreC-Nsphs cocultured with BMVNs exhibited enhanced neurite outgrowth compared to neurospheres in solo-culture, we sought to demonstrate that the Tuj1^+^ cells we observed were functional neurons. To test this, we cultured NPreC-Nsphs in MFDs with EGM-2:NMM under NPreC and NPreC-BEC-PC-AC conditions with IF. Before MFD culture, neurospheres were stained with CellTracker Red to monitor their position throughout culture and identify individual NPreCs and NPC-neurons. On day 1, NPreC-Nsphs were observed extending cellular processes into fibrin gels in both NPreC and NPreC-BEC-PC-AC conditions (Fig. 6A). By day 10, the area of the CellTracker Red signal had decreased for neurospheres cultured alone (Fig. 6A), indicating neurosphere regression and conforming with previous results (Fig. 5C). In contrast, the area of the CellTracker Red signal for NPreC-Nsphs cultured with BMVNs appeared larger on day 10 compared to day 1, confirming that neurospheres did not regress in quad-culture (Fig. 6A). On day 10, neurospheres in both NPreC and NPreC-BEC-PC-AC conditions were incubated with Fluo-8 to visualize Ca^2+^ oscillations within the cell bodies of differentiated NPreCs containing CellTracker Red (Fig. 6B). We found that the intracellular Ca^2+^ fluctuations of NPreC-Nsphs cultured alone were drastically different from those cultured with BMVNs. Of the 13 NPreC-Nsphs in NPreC conditions that were analyzed, we only observed spontaneously firing cells in two of them. Figure 6Bi shows representative time-lapse images of a 10-μm cross section of two adjacent NPreC-Nsphs cultured in NPreC conditions. Two distinct cells (highlighted by the yellow triangles) were observed exhibiting Ca^2+^ oscillations near the periphery of the neurosphere slice at the top-right of the images, as demonstrated by the change in Fluo-8 intensity over time (Fig. 6Bi). In contrast, no spontaneously firing cells were observed in the neurosphere slice located at the bottom-left of the same images (Fig. 6Bi). The latter neurosphere slice was more representative of those observed in NPreC conditions. In contrast, only 1 of the 13 analyzed NPreC-Nsph slices that were cultured with BMVNs did not contain any spontaneously firing NPC-neurons. Figure 6Bii shows representative images of one neurosphere slice in NPreC-BEC-PC-AC conditions in which three peripheral NPC-neurons (highlighted by the yellow triangles) exhibited intracellular Ca^2+^ fluctuations. We quantified neuron function for each neurosphere slice by measuring the number of NPC-neurons exhibiting Ca^2+^ oscillations and the frequency of Fluo-8 intensity spikes per minute. The mean number of firing NPC-neurons identified for neurosphere slices cultured under NPreC and NPreC-BEC-PC-AC conditions was 0.231 ± 0.166 and 3.077 ± 0.560, respectively (Fig. 6E). This demonstrated that significantly more NPC-neurons exhibited spontaneous neuronal activity in neurospheres cocultured with BMVNs compared to those in solo-culture. In addition, the pattern of Ca^2+^ oscillations was markedly distinct for firing NPC-neurons in NPreC and NPreC-BEC-PC-AC conditions. The Fluo-8 signal intensity measured for the few firing NPC-neurons in solo-culture gradually increased and decreased over the course of several minutes (Fig. 6, C and D). In contrast, firing NPC-neurons cultured with BMVNs frequently produced more distinguished spikes at much shorter intervals (Fig. 6, C and D). For these reasons, the mean total firing rate of neurosphere slices cultured under NPreC and NPreC-BEC-PC-AC conditions was 0.046 ± 0.033 and 1.077 ± 0.176 spikes/min, respectively (Fig. 6F). Together, these data showed that NPreC-Nsphs cultured with BMVNs were able to produce significantly more firing NPC-neurons with higher firing rates than NPreC-Nsphs cultured alone. This conformed with our previous data which concluded that the presence of BECs-tdT, PCs, and ACs enhanced NPreC neurogenesis in MFDs (Fig. 5F). Moreover, we confirmed that NPreCs differentiated in coculture with BMVNs produced functional neurons with the capacity to undergo spontaneous Ca^2+^ oscillations. This further validated the use of our human NVN model to investigate neuronal activity of developing neurons.

### IF enhanced neurogenesis of NPreCs cocultured with BMVNs in MFDs

**Fig. 7.**
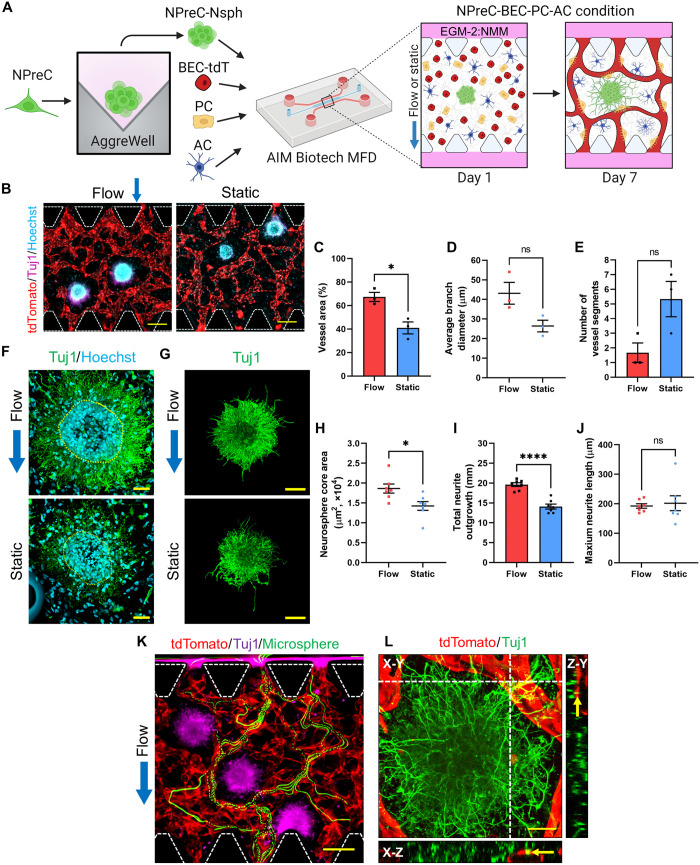
NPreC-Nsph neurogenesis in MFDs with and without IF. (**A**) Illustration of the culture protocol used. (**B**) Fluorescence MIPs of NPreC-Nsphs (Tuj1, purple) supported by BMVNs composed of BECs-tdT (tdTomato, red) under flow or static conditions on day 7. PCs and ACs not shown. Nuclei labeled with Hoechst (blue). (**C** to **E**) Graphs showing the vessel area (C), average branch diameter (D), and the number of vessel segments (E) measured for microvessels. Data from *n* = 3 MFDs. Welch’s *t* test; ns, *P* > 0.05; *P < 0.05. (**F**) Fluorescence confocal MIPs of NPreC-Nsphs cultured with BMVNs on day 7. NPC-neurons were labeled with Tuj1 (green) and Hoechst (blue). Yellow dotted lines outline neurosphere cores. (**G**) Large fluorescence confocal MIPs of NPreC-Nsphs (Tuj1, green). Background signals removed to highlight neurites. (**H** to **J**) Graphs showing the neurosphere core area (H), total neurite outgrowth (I), and maximum neurite extension (J) measured for NPreC-Nsphs. The data show mean value, error bars ± SEM, data from *N* = 7 neurospheres from n = 3 MFDs, Welch’s *t* test; ns, *P* > 0.05; *P < 0.05; ****P < 0.0001. (**K**) Fluorescence time-lapse MIP of microspheres (green) flowing through microvessels (tdTomato, red) supporting NPreC-Nsphs (Tuj1, purple) under flow conditions on day 7. (B) and (K) White dotted lines outline microposts. (**L**) Fluorescence confocal MIP of NPC-neurons (Tuj1, green) with microvessels (tdTomato, red) under flow conditions. Yellow arrows identify microvessel-neurite contact. Scale bars, 200 μm (B) and (K), 50 μm (F) and (L), and 100 μm (G). Blue arrows indicate direction of IF.

In this work, we demonstrated the benefit of coculturing NPreC-Nsphs with BMVNs composed of BECs-tdT, PCs, and ACs. Because of our previous publication detailing the critical role of IF in developing BMVNs in vitro (*30*), all previously discussed results involving NPreC-Nsphs were performed in MFDs with IF applied across the hydrogel channel. To emphasize the beneficial effect of IF on neurogenesis during quad-culture, we reestablished NPreC-BEC-PC-AC cultures with NPreC-Nsphs and maintained samples under either flow or static conditions (Fig. 7A). After 1 week, extensive BMVNs were observed around neurospheres stained for Tuj1 in both flow and static samples (Fig. 7B). BMVNs in flow conditions had significantly enhanced vessel area (67.30 ± 3.87%) when compared to static conditions (40.93 ± 5.10%; Fig. 7C). Although the mean value of the average microvessel branch diameter was lower in static culture (26.41 ± 2.99 μm) than in flow culture (43.14 ± 5.57 μm), no significant difference of means was found (Fig. 7D). Similarly, the mean number of vessel segments was larger in static conditions (5.00 ± 1.20) than in flow conditions (1.00 ± 0.67), but no significant difference was found (Fig. 7E). Although we found these results unexpected given the results from our previous publication (*30*), we attributed the lack of statistical significance to the low number of samples analyzed. Acquiring multiple regions of interest (ROIs) per sample to generate averages was considered; however, it was difficult to find regions of BMVNs without NPreC-Nsphs which would compromise the microvessel analysis.

Despite the results from microvessel analysis, we proceeded with analyzing the characteristics of NPreC-Nsphs to determine whether the application of IF influenced neurogenesis in quad-culture. After 1 week, NPreC-Nsphs cultured under flow and static conditions were observed extending Tuj1^+^ neurites into the fibrin gel (Fig. 7, F and G). However, neurospheres in static culture were visibly smaller and with less extensive neurite networks than those cultured in flow conditions. To quantify the influence of IF on NPreC-Nsph neurogenesis, we measured neurosphere core area, total neurite outgrowth, and maximum neurite extension for neurospheres cultured under flow and static conditions. The mean neurosphere core area was found to be enhanced for neurospheres in flow conditions (18,629 ± 2997 μm^2^) compared to those in static conditions (14,238 ± 2902 μm^2^; Fig. 7H). Similarly, the mean total neurite outgrowth for neurospheres was larger in flow conditions (19.61 ± 0.48 mm) than in static conditions (14.08 ± 0.62 mm; Fig. 7I). Last, no statistical difference was found between the mean maximum neurite extension for neurospheres culture under flow and static conditions, which were 192.55 ± 7.66 and 201.90 ± 25.24 μm, respectively (Fig. 7J). However, this was expected as the longest neurites observed for NPreC-Nsphs in both cultures were typically comparable in length. Together, these data indicated that the application of IF during quad-culture was beneficial for NPreC-Nsph neurogenesis. We surmised that the bulk fluid movement through the fibrin gel supplied neurospheres with sufficient soluble growth factors to undergo neuron differentiation and extend healthy neurites.

Fluorescent microspheres were observed flowing through BMVNs formed under flow conditions (Fig. 7K and movie S1). No microsphere perfusion assay was performed for static samples since we previously reported that BMVNs did not achieve anastomosis without the application of IF (*30*). Furthermore, microvessels cultured under static conditions would not experience any luminal flow during normal culture due to the absence of a hydrostatic pressure gradient across the hydrogel channel. In samples cultured under flow conditions, Tuj1^+^ NPreC-Nsphs were observed in close proximity to perfused microvessels, indicating that they were supported by microvessels experiencing luminal flow during culture (Fig. 7K and movie S1). Moreover, we observed direct contact between Tuj1^+^ neurites and tdTomato^+^ microvessels (Fig. 7L). These results were encouraging as they showcased the simultaneous processes of neurogenesis and vasculogenesis in an in vitro model. In conjunction with the enhanced neurosphere core area and total neurite outgrowth observed under flow conditions, these data justified the implementation of IF in quad-cultures with NPreC-Nsphs.

**Fig. 8.**
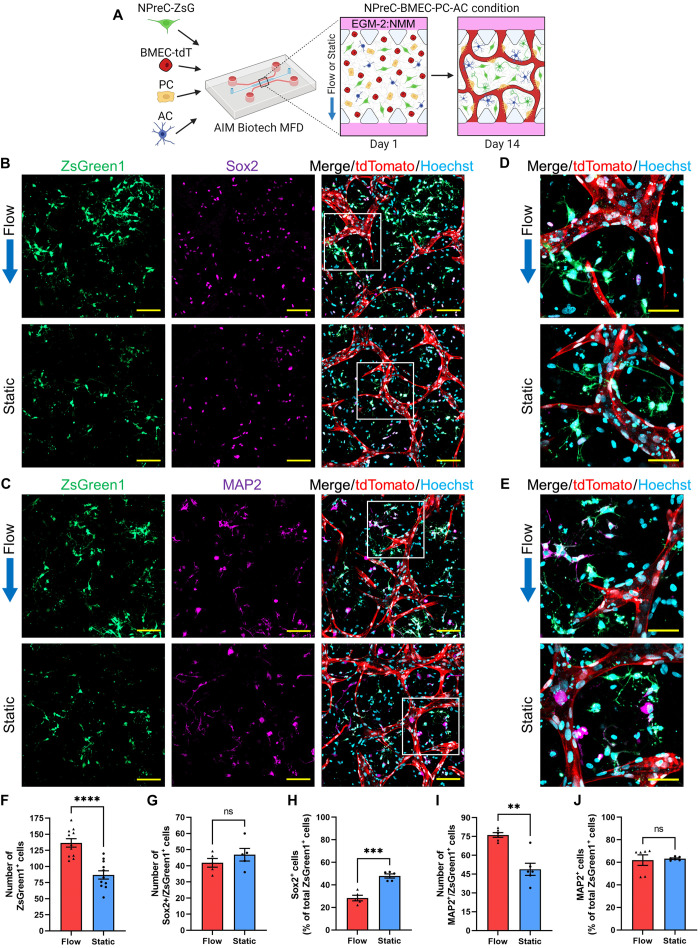
Self-renewal and neurogenesis of dispersed NPreCs with BMVNs in MFDs under flow and static conditions. (**A**) Illustration of the culture protocol used to investigate the effect of IF on dispersed NPreC self-renewal and neurogenesis. Briefly, NPreCs expressing ZsGreen1 (NPreCs-ZsG) were cultured with BMECs-tdT, PCs, and ACs in fibrin gels within MFDs. Samples were cultured in EGM-2:NMM under flow or static conditions for 2 weeks. (**B** and **C**) Fluorescence confocal MIPs of NPreCs-ZsG (ZsGreen1, green) expressing Sox2 (B, purple) and MAP2 (C, purple) with BMVNs in flow and static conditions on day 14. White square in merged image expanded in next sub-figure. (**D** and **E**) Enhanced MIPs of NPreCs-ZsG (ZsGreen1, green) expressing Sox2 (D, purple) and MAP2 (E, purple) in flow and static conditions on day 14. (B) to (E) BMECs-tdT were identified with tdTomato (red). Scale bars, 100 μm (B) and (C) and 50 μm (D) and (E). Blue arrows indicate the direction of IF in flow conditions. (**F** to **J**) Graphs of the number of ZsGreen1^+^ cells (F, *n* = 11), the number (G), and percentage (H) of Sox2^+^/ZsGreen1^+^ cells (*n* = 5), as well as the number (I) and percentage (J) of MAP2^+^/ZsGreen1^+^ cells (*n* = 6) measured in both flow and static samples on day 14. The data show mean value, error bars ± SEM, Welch’s *t* test; ns, *P* > 0.05; **P < 0.01, ***P < 0.001, ****P < 0.0001.

After we confirmed the importance of IF for maintaining NPreC-Nsphs cultured with BMVNs in MFDs, we wanted to determine whether neurogenesis would be improved when NPreCs were cultured as dispersed cells. To test this, NPreCs-ZsG were cultured with brain microvascular endothelial cells expressing tdTomato (BMECs-tdT), PCs, and ACs under flow or static conditions (Fig. 8A) under the differentiation medium EGM-2:NMM. NPreCs were labeled with ZsGreen (NPreCs-ZsG) so that the total cell population and their respective fate can be tracked and quantified. After 2 weeks, BMVNs were observed in both flow and static samples (Fig. 8, B and C). NPreCs-ZsG were observed dispersed throughout the interstitial space between microvessels in both conditions (Fig. 8, D and E). Sox2 expression was observed in both ZsGreen1^+^ and ZsGreen1^−^ cells, which identified self-renewing NPreCs-ZsG and those small amount Sox2^+^ cells among ACs population, respectively (Fig. 8, B and D). Similarly, MAP2 expression was observed in both ZsGreen1^+^ and ZsGreen1^−^ cells, which identified differentiated NPC-neurons and those MAP2^+^ cells among the ACs population, respectively (Fig. 8, C and E). Previously, we observed that human fetal ACs would randomly express certain amounts of MAP2 (fig. S6A) and Sox2 (fig. S6B) in some cells in 2D cell culture, this makes it difficult to analyze the Sox^+^ data. However, thanks to the labeling of ZsGreen1 in NPreCs-ZsG, we were able to distinguish the Sox2 and MAP2 signals belonging to NPreCs-ZsG from those belonging to ACs. From this, we were able to characterize NPreC-ZsG stemness and neurogenesis after 2 weeks in quad-culture under flow and static conditions. The mean total number of ZsGreen1^+^ cells observed in flow conditions (136.55 ± 6.53) was observed to be significantly higher than in static conditions (86.91 ± 6.42), indicating that more NPreCs-ZsG were present after 2 weeks with the application of IF than without flow (Fig. 8F). No significant difference was found between the mean number of Sox2^+^/ZsGreen1^+^ cells in flow (41.80 ± 2.20) and static (46.80 ± 3.20) conditions (Fig. 8G). Given the higher number of total ZsGreen1^+^ cells found in flow samples, this resulted in the percentage of Sox2^+^/ZsGreen1^+^ cells to be lower under flow conditions (28.32 ± 1.99%) compared to static conditions (47.89 ± 1.48%; Fig. 8H). These data indicated that the application of IF caused higher percentage of Sox2 down-regulation in NPreCs-ZsG cultured with BMVNs but still maintained the same total number of Sox2^+^ cells in the end due to the effect of higher total cell numbers. Together, these data together suggest that flow leads to differential regulation of NPreCs in terms of proliferation and self-renewal. We continued our investigation by measuring the effect of IF on the neuronal differentiation of NPreCs-ZsG. We found that the mean number of MAP2^+^/ZsGreen1^+^ cells was significantly higher under flow conditions (76.17 ± 1.90) than static conditions (48.83 ± 4.84; Fig. 8I). Using the total number of ZsGreen1^+^ cells per sample, these values resulted in the mean percentages of MAP2^+^/ZsGreen1^+^ cells being 61.97 ± 4.68 and 63.23 ± 0.65% under flow and static conditions, respectively (Fig. 8J). No significant difference was found for the mean percentage of MAP2^+^/ZsGreen1^+^ cells between flow and static conditions. Together, these results indicated that IF enhanced the total number of MAP2^+^/ZsGreen1^+^ cells in quad-culture but did not enhance the percentage of NPreCs-ZsG expressing MAP2, suggesting that IF promotes neurogenesis mainly through increasing total cell proliferation and then differentiated toward neuron lineage resulting in more neurons instead of favoring the percentage of differentiation. Last, samples cultured under flow conditions were also immunofluorescently labeled for GFAP. While we observed GFAP^+^ ACs contacting microvessels, no GFAP^+^/ZsGreen1^+^ cells were identified, indicating that no NPreC-ZsG astrogenesis occurred after 2 weeks of quad-culture in MFDs (fig. S7). Therefore, we concluded that culture in EGM-2:NMM condition mainly induced NPreC-ZsG neurogenesis instead of astrogenesis.

Similar to our investigation with NPCs, we were interested in the influence of NPreCs on vascular morphogenesis. To determine whether NPreCs produced an angiogenic effect, we stimulated BECs–enhanced green fluorescent protein (EGFP) to produce angiogenic sprouts into a fibrin matrix under BEC, BEC-PC, BEC-AC, and BEC-NPreC conditions. After 1 week in culture, BECs-EGFP were observed sprouting into hydrogel channels in all conditions (fig. S8A). However, sprouts in BEC and BEC-NPreC conditions barely extended to the tip of the microposts. Although larger sprouts were observed extending into the fibrin matrix in BEC-AC conditions, the largest and most numerous sprouts were seen in BEC-PC conditions (fig. S8A). In BEC-NPreC samples, NPreCs were labeled with Tuj1 to identify differentiated NPC-neurons. However, much of the Tuj1 signal appeared fragmented (fig. S8A). This likely indicated the presence of apoptotic NPreCs, corroborating with our previous results which showed that NPreCs did not survive extended periods of solo-culture in MFDs (fig. S5B). Angiogenic networks in all samples were characterized by sprout count (fig. S8B) and average sprout length (fig. S8C) on day 1, day 3, and day 7. Similar to the angiogenesis experiment with NPCs (fig. S4, A to C), NPreCs did not enhance either vascular parameter compared to BECs-EGFP cultured alone. This concluded that NPreCs had no pro-angiogenic effect on BECs-EGFP. Despite these results, we sought to determine whether NPreCs had any effect on vascular network formation using vasculogenesis assays in MFDs. After 1 week in culture, distinct vascular structures were formed in BEC, BEC-PC, BEC-AC, and BEC-NPreC conditions (fig. S8D). In BEC conditions, BECs-EGFP formed large microvessels with smaller, detached vessel segments. Microvessels grown with PCs formed thinner microvessels that were interconnected between the two fluidic channels. Microvessels in BEC-AC conditions occupied the least amount of area and appeared fragmented. We found this unexpected, since we previously observed vascular network formation of BECs-tdT in the presence of ACs (fig. S4D). However, this coculture was performed using EGM-2:NPM-2, not EGM-2:NMM. Last, microvessels were able to form in the presence of NPreCs but had visibly segmented vessels (fig. S8D). To quantify these observations, we measured vessel area, average branch diameter, and the number of vessel segments for all culture conditions. Similar to previous results with NPCs, no significant difference was observed in either the average branch diameter (fig. S8F) or the number of vessel segments (fig. S8G) between BEC and BEC-NPreC conditions. Only a slight decrease in vessel area was reported for microvessels cultured with NPreCs when compared to BECs cultured alone (fig. S8E). In conjunction with the results from the angiogenesis experiment, it can be said with reasonable certainty that NPreCs did not have any positive effect on vasculogenesis. While these results indicated that NPreCs did not influence endothelial cell morphogenesis, all previously reported cocultures with BECs and dispersed NPreCs were also accompanied by PCs and ACs to support proper microvessel network formation (Fig. 8, B and C).

## DISCUSSION

For all of our experiments, we used pluripotent stem cells XCL-1–derived NPCs on the basis that they generated homogeneous populations of nestin^+^/Sox2^+^ cells with the ability to be expanded for up to 10 passages and retain the potential to differentiate. Immunocytochemical analysis confirmed the presence of nestin and Sox2 in nearly all NPCs before any differentiation protocols. A majority of NPCs did not express either stem cell marker after the neuron differentiation protocol, confirming with the trend that NSCs/NPCs significantly down-regulate nestin and Sox2 expression as they commit to neuronal differentiation (*32*). Most NPC-ACs still expressed Sox2 and less than 10% expressed GFAP after 21 days of the astrocyte differentiation protocol, indicating that they still had stem cell–like characteristics. However, this is likely due to the relatively short length of the astrocyte differentiation protocol. Stemcell Technologies recommended culturing and passaging NPC-ACs in AMM for up to 100 days to maximize the expression of glial markers. However, we did not attempt this to conserve time and to focus on generating neurons from NPCs. After the neuron differentiation protocol, approximately 80% of NPC-neurons expressed Tuj1 and slightly over half expressed MAP2. These expression profiles are characteristic of NPCs as undergo neurogenesis and neuron maturation (*32*). We speculated that all NPC-neurons would continue to gain neuron marker expression if the maturation culture was extended to 5 weeks, as recommended by Stemcell Technologies. However, we considered 2 weeks to be sufficient time to detect and quantify NPC neuron differentiation in vitro. Although Stemcell Technologies provided specific protocols for neuron and astrocyte differentiation, XCL-1–derived NPCs were not advertised as being capable of generating oligodendrocytes. In the adult mammalian NVN, oligodendrocyte differentiation is rare compared to astrogenesis and neurogenesis. Only a small population of neuroblasts would eventually become oligodendrocyte progenitor cells in the rodent SVZ (*33*). Similarly, clonal lineage tracing of radial glia-like cells in the rodent SGZ revealed no oligodendrocyte differentiation from NSCs (*34*). For these reasons, we did not test if our NPCs were capable of oligodendrogenesis. Nonetheless, the capacity of NPCs for oligodendrogenesis was immaterial as we only intended to use our human NVN model to study NPC self-renewal and neurogenesis.

When we began our investigation, we originally planned to study the self-renewal and neurogenesis of individual NPCs within a 3D fibrin matrix. However, we consistently observed that most dispersed NPCs died after approximately 1 week when cultured alone in MFDs. LIVE/DEAD analysis confirmed that increasing the NPC seeding density, adding Matrigel to the ECM, removing EGM-2 from the culture medium, and terminating IF did not improve NPC viability. We concluded that the default microenvironmental conditions within MFDs were not conducive for dispersed NPC survival. Previous studies found that aggregation of NSCs/NPCs into neurospheres promoted cell proliferation and improved cell viability and metabolic activity (*35*, *36*). For these reasons, we expected NPC-ZsG-Nsphs to survive longer in MFDs than dispersed NPCs. However, culturing NPCs-ZsG as neurospheres also did not improve NPC survival in MFDs, regardless of NPC-ZsG density. As a whole, we found these results unexpected considering that fibrin-base hydrogels are widely used and have consistently been reported to support NSC/NPC maintenance and differentiation (*37*–*39*). The discrepancy between our results and those of previous studies could be explained by the source of our progenitor cells, as well as the specific transport phenomena observed within MFDs. Currently, the cause of XCL-1–derived NPC death in MFDs is unclear as we did not measure the expression of caspase proteins in NPCs to confirm the induction of apoptosis. At the very least, our results highlighted the inherent difficulty of maintaining viable NPCs in vitro in the MFDs. This served as the motivation for the rest of our investigation to generate a model of the human NVN capable of supporting NPCs for extended culture.

Initially, we hypothesized that NPC survival would be greatly improved when NPCs were cocultured with BMVNs within MFDs. We then discovered that neurosphere expansion was also enhanced when NPC-ZsG-Nsphs were cocultured individually with BECs-tdT, PCs, or ACs, as well as with all three cell types together, when compared to NPC-ZsG-Nsphs cultured alone. However, the largest increase in neurosphere area was observed in NPC-BEC and NPC-BEC-PC-AC conditions, emphasizing the vascular contribution of BECs-tdT to NPC-ZsG survival and expansion. Neurospheres in quad-culture expressed both Sox2 and Ki67, confirming that NPCs-ZsG were actively undergoing self-renewal and mitosis, respectively. Collectively, this indicated that neurosphere expansion was a product of cellular proliferation and not just the migration of NPCs-ZsG into the fibrin gel. These results were not unexpected, given the predominant role of endothelial cells in NSC/NPC proliferation and stemness (*6*). Shen and colleagues (*11*) famously demonstrated that soluble factors released from endothelial cells stimulated NSC self-renewal in vitro. In the brains of mice, vascular-derived NT-3 promoted NSC quiescence, which is paramount for NVN maintenance (*40*). NSC/NPC proliferation has also been shown to increase in response to endothelial cell–derived soluble amyloid precursor protein (sAPP) (*41*), laminin (*42*), vascular endothelial growth factor (VEGF) (*43*), and betacellulin (BTC) (*44*). In addition, both vascular brain-derived neurotrophic factor (BDNF) (*45*) and stromal cell-derived factor 1 (SDF1) (*46*) influenced NSC/NPC migration through chemotaxis. Last, MMPs secreted from endothelial cells have been shown to enhance NPC migration through the degradation of brain ECM (*47*). We found no difference in neurosphere expansion for NPC-ZsG-Nsphs in NPC-BEC-PC-AC and NPC-HUVEC-PC-AC conditions. This indicated that both endothelial cell lines had similar neurotrophic characteristics, despite the difference in cell source. However, this was not unexpected considering Shen and colleagues (*11*) found that both mouse BECs and bovine pulmonary artery endothelial cells enhanced NSC self-renewal and proliferation. Together with our results, these findings demonstrate that endothelial cells in general are beneficial to NSCs/NPCs through several cellular mechanisms.

It was unclear what specific cellular mechanisms enhanced neurosphere expansion when NPC-ZsG-Nsphs were cocultured with PCs. Although PCs are critical for vascular function in the NVN, there are few studies that have investigated the direct influence of PC-derived soluble factors on NSCs/NPCs (*48*). Stromal cells have previously been used as feeder cells to stimulate stem cell growth in vitro (*49*). Therefore, it is not improbable to assume that soluble factors secreted from PCs, such as VEGF (*50*), promoted NPC-ZsG proliferation during coculture. With regard to ACs, several sources have demonstrated that numerous glial-derived factors [glial cell-derived neurotrophic factor (GDNF), cerebral dopamine neurotrophic factor (CDNF), and BDNF] stimulate NSC proliferation (*51*) and neurogenesis (*52*). For these reasons, the increased neurosphere area that occurred in NPC-AC conditions was less unexpected than in NPC-PC conditions. Since no statistical difference was found between the mean neurosphere area ratio for NPC-ZsG-Nsphs cultured in NPC-BEC and NPC-BEC-PC-AC conditions, it was suggested that BECs-tdT were the predominant contributors to neurosphere expansion in quad-culture. In summary, the results from our neurosphere expansion experiment demonstrated the neurotrophic role of endothelial cells and justified the selection of primary BECs to recapitulate the human NVN in vitro.

Once we confirmed the benefit of coculturing NPC-ZsG-Nsphs with BECs-tdT, PCs, and ACs, we cocultured dispersed NPCs-ZsG with BMVNs under flow and static conditions using EGM-2:NPM-2 to determine the effect of IF on NPCs-ZsG proliferation and self-renewal. Initially, we were surprised to find that IF had no effect on microvessel morphology in quad-culture samples, considering our previous publication highlighted the benefit of IF on BMVN formation (*30*). However, the samples used in that study were cultured in EGM-2, not EGM-2:NPM-2. It is possible that the additional basic fibroblast growth factor (bFGF) and EGF present in NPM-2 influenced the morphology of microvessels in this study to such a degree that the effect of IF was overshadowed. Moreover, we observed no significant difference between the mean number of ZsGreen1^+^ cells in flow and static conditions, indicating that IF had no effect on NPC-ZsG survival in quad-cultures cultured in EGM-2:NPM-2. Originally, we had speculated that IF would be more efficient than simple diffusion at transporting soluble growth factors and wastes within 3D fibrin gels (*53*). This would mitigate the negative effects of cellular competition for nutrition and oxygen within the relatively crowded microenvironment of MFDs (*31*). However, previous microfluidic models have successfully relied on diffusion for nutrient delivery and waste removal (*26*, *54*). Since no statistical difference was found between the mean number of ZsGreen1^+^ cells in static and flow conditions, this indicated that both culture conditions were sufficient for NPC-ZsG survival and proliferation in this specific experimental setup. Moreover, NPCs-ZsG expressing Sox2 were observed directly contacting blood vessels in all samples. This behavior mimics that of native NSCs/NPCs located in the mammalian SVZ (*5*) and SGZ (*55*). Ottone and colleagues (*9*) demonstrated that direct cell-cell contact between NSCs and BECs maintained a population of quiescent NSCs in the NVN, likely through the binding of endothelial ephrin-B2 and Jagged to neural Eph and Notch receptors, respectively. In addition, direct contact between NSCs and BECs has been shown to promote the secretion of vascular cytokines (*56*). Although we did not label NPCs-ZsG with a cell proliferation marker, future studies will investigate the influence of microvessel contact on NPC quiescence using this quad-culture model. Because of the homogeneous expression of Sox2 in all NPCs-ZsG, we did not pursue an investigation of other stem cell markers using this experimental design. Instead, we explored that line of inquiry in sequential experiments that investigated NPreC neurogenesis.

When cultured alone in fibrin gels within MFDs, we observed that a majority of dispersed NPreCs died after 2 weeks. Although NPreCs survived longer in solo-culture than NPCs, these results confirmed that differentiating NPC to NPreCs did not prevent substantial cell death in MFD culture. Once again, this highlighted the inherent difficulty in successfully culturing healthy neural cells in vitro to study the cellular interactions of the human NVN. Despite the propensity for NPreCs apoptosis, small clusters of NPreCs were still observed in MFDs after 2 weeks. These observations indicated that homotypic cellular interactions may be necessary for extended NPreC survival and proliferation in the absence of supporting cells. Suspended keratinocyte sheets with intact intercellular connection were shown to have enhanced viability compared to suspended, single-cell keratinocytes (*57*). It has also been demonstrated that the viability of transplanted NSCs was significantly improved when cells were aggregated into neurospheres (*58*). It was for these reasons, in conjunction with our results, that we performed a majority of NPreC neurogenesis experiments using NPreC-Nsphs. Furthermore, in the surviving population of dispersed NPreCs in MFDs, we observed Sox2^+^/MAP2^−^, Sox2^+^/MAP2^+^, and Sox2^−^/MAP2^+^ cells in close proximity to each other. These cell types showcased the distinct stages of NPreC differentiation toward mature neurons. Initially, NPreCs still expressed Sox2 and therefore retained some stem cell–like characteristics of NPCs. As NPreCs were exposed to neurotrophic growth factors in EGM-2:NMM, they began to down-regulate Sox2 and up-regulate MAP2 expression as they became mature neurons. This pattern matched the representative expression profile of NSCs as they differentiate and mature into interneurons in vivo (*59*). Moreover, we did not observe any instances of NPreC astrogenesis in MFD culture when using EGM-2:NMM as the culture medium. Although this was not unexpected, as only a small percentage of NPC-neurons expressed GFAP when cultured in unadulterated NMM in 2D cell culture. Although bona fide NSCs have the potential to differentiate into ACs, a majority of differentiation in the mammalian SVZ is directed toward a neuronal fate (*5*). This was the justification for focusing our investigation on NPreC neurogenesis in this work.

We found that NPreC-Nsph neurogenesis, measured by total neurite extension, was enhanced when neurospheres were cocultured individually with BECs, PCs, or ACs, as well as with all three cell types together, when compared to NPreC-Nsphs cultured alone. Given the results of the LIVE/DEAD assay performed using dispersed NPreCs in MFDs, these observations were not unexpected. The sparse cores of NPreC-Nsphs in solo-culture indicated that a notable percentage of NPreCs migrated into the fibrin gel and committed apoptosis, reaffirming our conclusion that both NPCs and NPreCs do not survive extended periods in MFDs without supporting cells. We expected coculture with BECs-tdT to enhance neuritogenesis, due to the well-documented neurotrophic effect of endothelial cells (*6*). However, we did not expect PCs and ACs to improve neurite outgrowth to the same degree. Ehret and colleagues (*60*) showed that BECs, PCs, and ACs all enhanced the survival of NPCs isolated from the rodent SGZ, although only BECs increased neuronal differentiation. PCs have a multiple roles, including the regulation of the BBB, microvascular remodeling, control of cerebral blood flow, and neuroinflammation maintenance (*61*). However, the majority of these roles involve complex coordination with BECs. It was unclear how the coculture of NPreC-Nsphs with PCs significantly improved neurogenesis without the presence of BECs. Future experiments will be needed to identify the specific PC-derived soluble factors, such as VEGF (*50*), which influenced neurite outgrowth. In the NVN, ACs also interact with BECs through perivascular endfeet to regulate BBB function, as well as bridge the intercellular communication between BECs and neurons (*62*). However, ACs have also been shown to directly influence neurogenesis, as well as synapse formation and function, through paracrine and juxtacrine signaling (*63*). Therefore, the increased total neurite outgrowth observed for NPreC-Nsphs in NPreC-AC conditions was not as unexpected as in NPreC-PC conditions. We expected NPreC survival to be improved in NPreC-BEC conditions since vascular-derived EGF and bFGF have been shown to increase NSC/NPC proliferation in vivo (*6*). Moreover, Shen and colleagues (*11*) demonstrated that initially coculturing NSCs with endothelial cells stimulated preferential differentiation to neurons, likely due to the release of vascular-derived soluble factors. On the basis of these findings, we inferred that microvessels developed in NPreC-BEC and NPreC-BEC-PC-AC conditions provided paracrine signals that improved NPreC viability and facilitated neuritogenesis. After 10 days in MFDs, NPreC-Nsphs cultured in NPreC-BEC conditions had the largest neurosphere core area compared to all other conditions, suggesting that vascular-derived factors also promoted NPreC proliferation. In quad-culture samples, we observed neurites from NPreC-Nsphs directly contacting microvessels. Although angiogenesis and neurogenesis have coordinated interactions (*64*), few studies have remarked on the direct juxtacrine signaling between blood vessels and neurons. In the brain, the communication between endothelial and neuronal cells is normally mediated by ACs (*62*). However, Osaki and colleagues (*28*) reported that juxtacrine signaling (via Dll4-Notch1 and Jag1-Notch1) between HUVECs and motor neuron spheroids up-regulated neuron differentiation and activity in vitro. Considering that neurospheres in our model were composed of NPreCs, we speculated that direct contact with microvessels enhanced NPreC neurogenesis and maturation. The total neurite outgrowth was significantly lower in NPreC-BEC-PC-AC conditions when compared to NPreC-BEC conditions. Superficially, these results implied that the addition of PCs and ACs was not beneficial for neurite outgrowth of NPreC-Nsphs. However, this was unconvincing, as healthy PCs and ACs normally have a neuroprotective role in the brain (*62*, *65*). Rather, the diminished neurite outgrowth of NPreC-Nsphs in NPreC-BEC-PC-AC conditions was most likely the result of increased cellular competition within the fibrin gel (*31*). On day 0, the initial dispersed cell densities in NPreC-BEC and NPreC-BEC-PC-AC conditions were 6 × 10^6^ and 10 × 10^6^ cells/ml, respectively. It is doubtful that the addition of PCs and ACs did not affect the competition for growth factors from the culture medium as well as the generation of metabolic wastes. Also, the additional cells in quad-culture likely acted as physical barriers to neurites as they extended into the fibrin gel. Therefore, we concluded that the decreased neurite outgrowth in quad-culture samples was likely the result of the total number of cells in the hydrogel channel and not any antagonistic effects of PCs or ACs. With that said, an in vitro model of the human NVN will need to include BECs, PCs, and ACs to generate physiologically accurate BMVNs. For this reason, we elected to use our quad-culture model to further investigate the influences of NPreC neurogenesis.

With regard to cell fate, the results from our model emphasized how the use of different cell types (NPCs versus NPreCs) and different culture media (EGM-2:NPM-2 versus EGM-2:NMM) resulted in different cell behaviors (self-renewal versus neurogenesis) when cocultured with BMVNs. These data not only highlighted the versatility of our experimental system but also illustrated how biological stimuli can have different cellular effects depending on the developmental stage of the target cell. BDNF is a prominent vascular-derived neurotrophin in the NVN and has been reported to regulate neuron maturation, neuroblast migration, as well as NPC recruitment and proliferation (*6*). Therefore, it was not unexpected that coculture with BMVNs enhanced both NPC proliferation and NPreC neurogenesis in our model. These observations replicated the delicate balance of NSC/NPC self-renewal and neuron differentiation that occurs in the adult NVN in vivo (*3*, *5*). In this work, we describe multiple protocols needed to successfully model and analyze these stem cell behaviors in vitro.

When NPreC-Nsphs were cocultured with BECs-tdT, PCs, and ACs under flow conditions, we observed the formation of perfused BMVNs (movie S1). This demonstrated that the hydrostatic pressure gradient that induced IF across the fibrin gel also promoted intravascular flow after BMVN anastomosis was achieved. We hypothesized that IF, as well as luminal flow in microvessels, would be beneficial for NPreC-Nsph neurogenesis. Perfused microvessels likely facilitated the delivery of nutrients and differentiation growth factors to NPreC-Nsph located in the interior of the fibrin gels. Vascular delivery likely mitigated the negative effects of cellular competition due to the relatively high total cell density in NPreC-BEC-PC-AC conditions (*31*). The benefit of the application of IF on neurogenesis was made evident when NPreC-Nsphs were cultured with BMVNs under flow and static conditions. After 1 week, the blood vessels formed under flow conditions covered significantly more area than the microvessels grown under static conditions, likely due microvessel perfusion which was only present in flow samples. In addition, both the mean neurosphere core area and total neurite outgrowth were found to be enhanced in flow conditions. We speculated that the increased neurosphere size and neurite extensions were the result of IF in the hydrogel channels. This is not to say that the bulk movement of interstitial fluid provided mechanical stimulation that expedited neuron differentiation. Rather, IF was likely more efficient at providing nutrients and removing wastes than simple diffusion (*53*). For this reason, the bioavailability of growth factors from the differentiation medium was likely higher in flow conditions due to conductive transport. It has been demonstrated that the movement of brain interstitial fluid is paramount for normal brain function in vivo (*18*). Recent studies revealed that the disruption of the glymphatic pathway, which clears interstitial fluid from the brain extracellular space, was correlated with an increased accumulation of amyloid β and neurological dysfunction in Alzheimer’s disease (*66*). We previously showed that the maximum IF velocity obtained in our BMVN model (5.73 μm/s) (*30*) was within the physiological range found in most soft tissues, including the brain (0.1 to 10 μm/s) (*67*, *68*). This indicates that our in vitro model has the capability to properly model the effect of IF disruption on human neurodegenerative disease progression. Furthermore, it is possible that the application of luminal flow through microvessels improved neurogenesis through the up-regulation of neurotrophic factors in BECs. Dumont and colleagues (*69*) demonstrated that endothelial cells exposed to fluidic shear stress promoted the differentiation of NPCs through the secretion of soluble growth factors. In addition, Osaki and colleagues (*28*) showed that the concentration of vascular-derived BDNF was enhanced when motor neuron spheroids were cocultured with perfused HUVEC microvessels. With regard to microvessel perfusion, we previously published that the mean shear stress experienced by microvessels cultured under flow conditions (0.394 dyn/cm^2^) (*30*) was within the physiological range reported in vasculature throughout the human body (0.1 to 60 dyn/cm^2^) (*70*, *71*). For these reasons, our NVN model will be valuable in future studies to determine if the application of shear stress from intravascular flow enhances neurogenesis through the production of BEC-derived neurotrophins.

Analysis of Ca^2+^ oscillations of NPreC-Nsphs in solo-culture and quad-culture revealed insight on the neuronal function of NPC-neurons in vitro. We rarely observed spontaneous neuronal activity in NPC-neurons within neurospheres that were cultured alone. In contrast, we typically identified several firing NPC-neurons in neurospheres that were cocultured with BMVNs. All firing NPC-neurons were located near the periphery of NPreC-Nsphs. However, this was not unexpected considering that the border of neurospheres likely provided NPC-neurons easier access to soluble neurotrophins and more physical room to extend neurites, when compared to the neurosphere center. The time scale of the Ca^2+^ concentration dynamics we observed in NPreC-Nsphs was similar to those reported from neurons cultured in vitro (*72*). The number of firing cells and the total spikes per minute was significantly higher in neurospheres cultured in NPreC-BEC-PC-AC conditions when compared to NPreC conditions. We did not find these results unexpected, given the relatively sparse neurite outgrowth of NPreC-Nsphs in solo-culture. These data highlighted the beneficial role of BMVNs in the development of functional neurons in vitro, which has been demonstrated in several previous studies. Wu and colleagues (*73*) showed that the neurovascular interaction of primary BECs and cortical neurons improved neurite outgrowth and accelerated electrophysiological development in 2D cell culture. In addition, Sances and colleagues (*25*) developed a MFD that cultured spinal NPCs and endothelial cells in two fluidic channels separated by a porous membrane. Although this study reported enhanced neuronal function of differentiated spinal NPCs during coculture with endothelial cells, both cell types were cultured on 2D surfaces. Last, Osaki and colleagues (**28**) recently developed 3D vascular networks of HUVECs which enhanced the neurogenesis and Ca^2+^ oscillations of motor neuron spheroids. We present the first microfluidic model of spontaneously firing NPreC-Nsphs cocultured with perfused BMVNs composed of primary BECs, PCs, and ACs. Compared to the previously mentioned studies, our BMVNs better recapitulated the brain capillaries found in the human NVN (*8*). We postulated that longer culture periods with BMVNs would continue the maturation of NPreCs to neurons with Ca^2+^ oscillations akin to those found in the human brain. Nonetheless, this experiment demonstrated the efficacy of our quad-culture model to measure the influence of BMVNs on neuronal function in a microenvironment similar to the NVN. This ability will be useful for modeling the electrophysiological development of immature neurons in the developing brain, as well as understanding how the pathogenesis of specific neurodegenerative diseases affects neuron activity.

Coculturing dispersed NPreCs-ZsG with BMVNs under flow and static conditions confirmed that neurogenesis, but not the percentage of neuron differentiation, was enhanced due to the application of IF. More ZsGreen1^+^ cells were present in flow conditions than static conditions on day 14. On the basis of the number of ZsGreen1^+^ cells alone, we cannot conclusively say that the final number of NPreCs-ZsG was due to NPreC maintenance or proliferation. Endothelial cell–derived sAPP (*41*), laminin (*42*), VEGF (*43*), and BTC (*44*) have all been shown to increase the proliferation of NSCs/NPCs. These data, in conjunction with the presence of Ki67^+^/ZsGreen1^+^ cells in NPC-Nsphs from our prior experiment, led us to surmise that the final number of NPreCs-ZsG was attributed to cell division. Moreover, although the mean number of Sox2^+^/ZsGreen1^+^ cells was not significantly different between flow and static conditions, a higher percentage of Sox2^+^/ZsGreen1^+^ cells was observed in static conditions. Collectively, these data indicated that IF enhanced the number of NPreCs-ZsG cocultured with BMVNs; however, a lower percentage of these NPreCs-ZsG expressed Sox2. This led us to speculate that the application of IF facilitated NPreC-ZsG differentiation. More MAP2^+^/ZsGreen1^+^ cells were observed in flow samples than static samples on day 14. However, we observed no difference in the mean percentage of MAP2^+^/ZsGreen1^+^ cells between flow and static cultures. Therefore, the higher number of MAP2^+^/ZsGreen1^+^ cells in flow conditions was likely the result of the larger total population of NPreCs-ZsG. We concluded that IF enhanced NPreC-ZsG neurogenesis by promoting the differentiation of a higher number, but not percentage, of MAP2^+^ neurons. These results demonstrated that our model recapitulates the cellular interactions and neuronal differentiation observed in the mammalian NVN. To continuously repopulate the olfactory bulb with interneurons throughout rodent adulthood, a steady supply of neuroblasts is generated in the SVZ from a smaller population of self-renewing NSCs (*74*, *75*). Observations in our quad-culture model mimicked this dynamic interaction of self-renewal and neurogenesis. A small population of NPreCs-ZsG expressed Sox2 on day 14, confirming their potential for self-renewal. Since mature neurons typically do not undergo cell division (*76*), these Sox2^+^ cells will be beneficial for the generation of new NPreCs-ZsG for further differentiation into NPC-neurons. We predict that if quad-cultures were extended for longer time periods, then there would still be a small population of Sox2^+^/ZsGreen1^+^ cells present in the MFDs. In summary, these data demonstrated that our model would be appropriate to investigate how the bulk flow of interstitial fluid influences the development of mature neurons cocultured with BMVNs. Furthermore, the potential for our NVN model to be cultured beyond 2 weeks is of particular importance when modeling neurodegenerative disorders that decrease neuronal function gradually, such as Alzheimer’s disease and brain tumors.

Since we did not report any pro-angiogenic and pro-vasculogenic effects from either NPCs or NPreCs, the results from these experiments were moved to the Supplementary Materials (fig. S4 or S8, respectively). However, we determined that the conclusions from the angiogenesis and vasculogenesis assays would be valuable to researchers exploring this line of inquiry. As expected, when BECs were cocultured with either PCs or ACs, we observed enhanced microvessels formation compared to BECs cultured alone. Endothelial cells and PCs communicate via paracrine and juxtacrine signals to regulate blood vessel sprouting, branching, and permeability (*77*). Similarly, pro-angiogenic factors released from reactive ACs have been shown to promote revascularization of brain regions afflicted by stroke (*78*). However, previous studies have also reported improved microvessel network formation and stabilization when endothelial cells were cocultured with NSCs/NPCs (*12*–*14*). On the basis of these studies, we expected marked improvement in vascular characteristics when BECs were cocultured with either NPCs or NPreCs. However, the discrepancy with our observations could be the result of the specific cell types and culture media used during experimentation. The soluble factors secreted by iPSC-derived NPCs in vitro likely differ from those produced by bona fide human NSCs in the brain. It is also possible that the combination of BECs with NPCs or NPreCs in EGM-2:NPM-2 or EGM-2:NMM, respectively, was simply not conducive to vascular network formation in 3D fibrin gels. Furthermore, NPC and NPreC apoptosis likely resulted in the disruption of their cell membranes and the release of intracellular components into the fibrin gel. Cells undergoing apoptosis typically release “danger signals” that can have nefarious effects on neighboring cells (*79*). This could explain the diminished vascular sprouting and network formation experienced when BECs were cocultured with either NPCs or NPreCs, when compared to BECs cultured alone. Future experiments will be conducted to test for the presence of inflammatory cytokines in the fibrin gel after neural cell death. Regardless, the purpose of the angiogenesis and vasculogenesis assays was simply to determine whether NPCs or NPreCs individually influenced endothelial cell morphogenesis. In our quad-culture models, NPCs or NPreCs were cocultured with PCs and ACs, which predominantly governed BEC microvessel formation. Therefore, the lack of any angiogenic or vasculogenic characteristics of NPCs and NPreCs was ultimately immaterial for our objective to create a physiologically accurate in vitro model of the human NVN.

Recent studies have shown that the preservation of a small stem cell pool is critical for continuous neuronal remodeling and repair in vivo (*5*, *80*). Premature depletion of this stem cell population can be detrimental as this would result in the cessation of neurogenesis, which occurs during aging and neurodegenerative diseases. Our current data suggest that BMVNs and flow dynamically regulate the delicate balance of NPC proliferation, self-renewal, and differentiation. Therefore, our model will be valuable to study how blood vessels influence stem cell fate with respect to biochemical factors (cell type, growth factors, and ECM composition), oxygen levels (normoxia and hypoxia), as well as IF and intravascular flow. Future experiments using this design will be conducted with improved methods of biochemical analysis. A major constraint of our current model is the inability to extract sufficient quantities of cells to perform reverse transcription polymerase chain reaction and Western blotting. However, Campisi and colleagues (*54*) were able to circumvent this limitation by removing the bottom layer of their larger MFD and pooling samples to generate sizable cell populations for group analysis. In our future experiments, individual populations of NPCs-ZsG, NPreCs-ZsG, and BECs-tdT will be isolated using fluorescence-activated cell sorting to provide insight on the cell-specific genes and proteins that regulate self-renewal and neurogenesis. Last, we previously implemented enzyme-linked immunosorbent assay to measure the influence of IF on the secretion of BDNF from BMVNs (*30*). We will further modify this protocol to detect soluble factors released in our system that regulate NPC fate and behavior. This will be of particular importance when determining how flow-induced microvessel shear stress modulates the secretion of endothelial cell–derived growth factors in our human NVN model.

In conclusion, this study demonstrated the capability of our microfluidic model of the human NVN to identify and evaluate the influences of NPC fate and behavior. In the first half of this work, we cocultured NPCs with perfused BMVNs composed of BECs, PCs, and ACs to investigate NPC proliferation and self-renewal. We identified the beneficial role of BECs, PCs, and ACs in NPC-ZsG-Nsph survival and expansion. In addition, self-renewing NPCs-ZsG were observed directly contacting brain microvessels, confirming the ability of our model to recapitulate the intricate cellular interactions of the human NVN in vitro. In the second half of this work, we cocultured NPreCs with BMVNs to determine the influences of neuron differentiation in our model. Coculture of NPreC-Nsphs with BECs, PCs, and ACs enhanced neurogenesis, as confirmed by increased neurite outgrowth. In addition, NPreC-Nsphs cocultured with BMVNs produced differentiated NPC-neurons which exhibited spontaneous calcium oscillations. These results demonstrated the potential of our model to evaluate neuron function in vitro. The significance of interstitial fluid flow was highlighted when BMVNs were cocultured with either NPreC-Nsphs or dispersed NPreCs-ZsG. The application of IF enhanced neurogenesis, as measured by an increase in the neurite outgrowth from neurospheres and the total number of differentiated, mature NPC-neurons. The analysis of Sox2 and MAP2 expression in dispersed NPreCs-ZsG cocultured with BMVNs further validated the efficacy of our NVN model to study the dynamic fate of individual stem cells in vitro. Last, the development of perfused, functional BMVNs will be valuable for preclinical studies to determine the efficacy of brain therapeutics that rely on vascular delivery. Modifications to our current experimental design can be implemented to further study the neurovascular interactions that occur during human embryogenesis as well as neurodegenerative diseases.

## MATERIALS AND METHODS

### Cell culture

In this work, all cells were incubated at 37°C and 5% CO_2_. Human pluripotent stem cell (XCL-1 line)–derived NPCs (Stemcell Technologies, catalog no. 70901, male) were expanded on tissue culture dishes coated with Matrigel hESC-Qualified Matrix (Corning, catalog no. 354277) in NPM-2 (Stemcell Technologies, catalog no. 08560). NPCs were harvested between passages 3 and 5 using Accutase (Thermo Fisher Scientific, catalog no. A1110501).

Primary human brain microvascular endothelial cells were ordered from ScienCell (BECs, catalog no. 1000, male) and Cell Systems (BMECs, catalog no. ACBRI 376, male). HUVECs were ordered from Lonza (catalog no. C2519AS, male/female pooled). All endothelial cells were expanded on tissue culture flasks coated with 0.2% porcine gelatin (Sigma-Aldrich, catalog no. G1890) in EGM-2 (PromoCell, catalog no. C-22010) with 1% penicillin/streptomycin (Thermo Fisher Scientific, catalog no. SV30010). All endothelial cells were harvested at passages 4 and 5 using 0.25% trypsin-EDTA (Thermo Fisher Scientific, catalog no. 25-200-056).

Primary human brain vascular pericytes (PCs, ScienCell, catalog no. 1200, male) and astrocytes (ACs, ScienCell, catalog no. 1800, male) were expanded on tissue culture flasks coated with poly-l-lysine (5 μg/ml; ScienCell, catalog no. 0413) in pericyte medium (ScienCell, catalog no. 1201) and astrocyte medium (ScienCell, catalog no. 1801), respectively. PCs and ACs were harvested between passages 2 and 4 using trypsin-EDTA.

To visualize specific cell types, select cell lines were made to express fluorophores using a lentiviral transduction protocol. BECs, BMECs, and HUVECs were made to express EGFP (Vector Builder, catalog no. VB150915-10026) or tandem dimer Tomato fluorescent protein (tdTomato, Vector Builder, catalog no. VB181014-1005thm). NPCs were made to express ZsGreen1 fluorescent protein (ZsGreen1, Vector Builder, catalog no. VB181014-1006ufx). Briefly, all cells were seeded and cultured according to their specific culture protocols. After 18 hours, cells were introduced to cell culture medium containing polybrene (5 μg/ml; Thermo Fisher Scientific, catalog no. TR1003G) and the manufacturer’s recommended functional titer concentration of lentiviruses. After 24 hours of exposure to the transduction medium, cells were allowed to recover in unadulterated cell culture medium for an additional 24 to 48 hours. Next, cell selection was performed by adding culture medium containing either blasticidin (10 μg/ml; Sigma-Aldrich, catalog no. 15205) or puromycin (2 to 4 μg/ml; Sigma-Aldrich, catalog no. P8833), depending on the antibiotic resistance engendered from transduction. BECs expressing tdTomato (BECs-tdT), BECs expressing EGFP (BECs-EGFP), BMECs expressing tdTomato (BMEC-tdT), and HUVECs expressing tdTomato (HUVECs-tdT) were expanded in EGM-2 and harvested at passages 5 to 6 using trypsin-EDTA. NPCs expressing ZsGreen1 (NPCs-ZsG) were expanded in NPM-2 and harvested between passages 4 and 6 using Accutase.

### NPC differentiation

NPCs were differentiated to NPC-derived astrocytes (NPC-ACs) or NPC-derived neurons (NPC-neurons) through astrocyte or neuron differentiation-maturation kit protocols, respectively, according to the instructions from Stemcell Technologies (Fig. 1A). To begin, NPCs were cultured on Matrigel-coated culture dishes in NPM-2 until they reached 90% confluency and then harvested for further differentiation.

In the astrocyte differentiation protocol, NPCs were initially cultured on Matrigel-coated culture dishes in NPM-2 for 24 hours. The culture medium was then replaced with astrocyte differentiation medium (ADM, Stemcell Technologies, catalog no. 08540). When cells reached 90% confluency, NPCs were passaged and cultured on new Matrigel-coated dishes in ADM. By day 14 of the differentiation protocol, cells were considered to be APreCs. APreCs were harvested using Accutase and seeded on new Matrigel-coated dishes and cultured in AMM (Stemcell Technologies, catalog no. 08550). When cells reached 90% confluency, APreCs were passaged again and cultured on new Matrigel-coated dishes in AMM. By day 21 of the astrocyte differentiation protocol, cells were considered to be NPC-ACs.

In the neuron differentiation protocol, NPCs were initially cultured on tissue culture dishes coated with poly-l-ornithine (PLO; Thermo Fisher Scientific, catalog no. A004C) and mouse laminin (Thermo Fisher Scientific, catalog no. CB-40232) in NPM-2 for 24 hours. The culture medium was then replaced with neuron differentiation medium (NDM; Stemcell Technologies, catalog no. 08500). By day 7 of the differentiation protocol, NPCs were considered to be NPreCs. NPreCs were harvested using Accutase and seeded on new PLO/laminin-coated culture dishes in NMM (Stemcell Technologies, catalog no. 08510). No additional passaging was necessary. By day 21 of the neuron differentiation protocol, cells were considered to be NPC-neurons. The same methods were used to differentiate NPCs-ZsG to NPreCs expressing ZsGreen1 (NPreCs-ZsG).

### BMVN generation in MFDs

In a previous manuscript, we developed perfused, 3D BMVNs within MFDs purchased from AIM Biotech (catalog no. DAX-1) (*30*). Briefly, bovine fibrinogen (8 mg/ml; Sigma-Aldrich, catalog no. NC1042134) and thrombin (4 U/ml; Sigma-Aldrich, catalog no. 50-165-7274) were dissolved in EGM-2 and phosphate-buffered saline (PBS; Thermo Fisher Scientific, 14-040-182), respectively. Next, BECs-tdT, PCs, and ACs were harvested and resuspended together in fibrinogen solutions. Fibrinogen-cell solutions were combined at a 1:1 ratio with thrombin solutions and immediately injected into the hydrogel channel of MFDs. Fibrin hydrogels (final concentration: 4 mg/ml) were allowed to polymerize at room temperature (RT) for 10 min. The final cell density of BECs-tdT, PCs, and ACs was 6 × 10^6^, 2 × 10^6^, and 2 × 10^6^ cells/ml, respectively. All samples were cultured in EGM-2 supplemented with aprotinin (5 μg/ml; Sigma-Aldrich, catalog no. A3428) for the entire culture duration and with VEGF (50 ng/ml; Thermo Fisher Scientific, catalog no. 50-398-745) until day 3. MFDs were cultured under either flow or static conditions. In flow conditions, 80 μl of culture medium was added to both MFD reservoirs of one fluidic channel, and 40 μl was added to both reservoirs of the opposite channel. This generated a hydrostatic pressure difference (1.5 mmH_2_O) between fluidic channels and induced IF across the fibrin gel within the hydrogel channel. In static conditions, 60 μl of culture medium was added to all reservoirs, which induced no IF across the fibrin gel. All MFD samples were incubated (37°C and 5% CO_2_) for at least 1 week with daily culture medium replenishment. In this work, modifications that were made to this general MFD culture protocol in several different experiments will be highlighted where necessary. In this work, all culture media used in MFD cultures were supplemented with aprotinin and VEGF as described in this section.

### Immunocytochemistry protocol

Samples were labeled using immunocytochemistry techniques to identify specific proteins. For cells in 2D culture, samples were washed in PBS three times for 5 min each. Next, samples were fixed in 4% paraformaldehyde (PFA; Thermo Fisher Scientific, catalog no. AAJ61899AP) for 15 min at RT and washed three times in PBS. Samples were then simultaneously permeabilized and blocked using a PBS solution composed of 10% normal goat serum (Thermo Fisher Scientific, catalog no. ICN19135680), 0.2% Triton X-100 (Thermo Fisher Scientific, catalog no. BP151), and 0.1 M glycine (Thermo Fisher Scientific, catalog no. BP381) for 1 hour at RT. Afterward, samples were exposed to the same permeabilizing/blocking (P/B) solution with primary antibodies for 1 hour at RT. BECs and PCs were labeled for platelet endothelial cell adhesion molecule (CD31) and neuron-glial antigen 2 (NG-2), respectively. Self-renewing NPCs and NPreCs were labeled for either nestin or Sox2. Primary ACs and NPC-ACs were both labeled with GFAP. NPC-neurons were labeled with either class III β-tubulin (Tuj1) or MAP2. Last, proliferating cells were labeled with antigen KI-67 (Ki67). After primary antibody incubation, samples were washed three times with PBS. Last, samples were exposed to P/B solution with secondary antibodies and Hoechst 33342 (1:1000; Thermo Fisher Scientific, catalog no. H3570) for 30 min at RT followed by three final washes in PBS. For cells in MFDs, the following modifications were made to the immunocytochemistry protocol: (i) All washes with PBS were performed for 1 hour; (ii) samples were fixed with 4% PFA for 1 hour; and (iii) all steps involving P/B solution were performed overnight at 4°C. After the final wash steps, all 2D and MFD samples were stored at 4°C until needed. Specific information about the antibodies used in this work can be found in tables S1 and S2.

### Dispersed NPCs or NPreCs in MFDs

NPCs and NPreCs were cultured alone in MFDs to characterize their solo behavior under different culture conditions. Both cell types were seeded and cultured in MFDs using methods similar to those described in the “BMVN generation in MFDs” section. Briefly, NPCs or NPreCs were resuspended in fibrin gels within the hydrogel channels of MFDs. The final cell density of both cell types was 2 × 10^6^ cells/ml. Samples containing NPCs or NPreCs were cultured in a 1:1 mixture of EGM-2 and NPM-2 (EGM-2:NPM-2) or NMM (EGM-2:NMM), respectively, under flow conditions for up to 2 weeks.

After we observed that NPCs typically died in MFDs after approximately 1 week, we made the following protocol modifications to improve NPC survival. In one experiment, NPCs were introduced to MFDs at 2 × 10^6^, 4 × 10^6^, and 6 × 10^6^ cells/ml to determine the effect of cell density on NPC viability. In a separate experiment, Matrigel was added to the fibrin hydrogel to determine if modifying ECM composition influenced NPC survival. Briefly, Matrigel (10.98 mg/ml stock concentration) and fibrinogen were added to the same solution of EGM-2 at 4°C such that the concentrations were 5 and 8 mg/ml, respectively. NPCs were resuspended at 4 × 10^6^ cells/ml in a PBS solution with thrombin (4 U/ml). Thrombin-cell solutions were combined at a 1:1 ratio with fibrinogen or fibrinogen-Matrigel solutions and quickly introduced to MFDs and allowed to polymerize. The final concentrations of Matrigel, fibrin, and NPCs in the hydrogel channels were 2.5 mg/ml, 4 mg/ml, and 2 × 10^6^ cells/ml, respectively. In one final experiment, we sought to determine whether the default culture conditions caused NPCs to die inside MFDs. To test this, NPCs (2 × 10^6^ cells/ml) were resuspended in fibrin and cultured in EGM-2:NPM-2 under flow conditions as the control group. Next, NPCs were cultured in NPM-2 under flow conditions to determine if the presence of EGM-2 was detrimental to NPC viability. Last, NPCs were cultured in EGM-2:NPM-2 under static conditions to determine if the application of IF induced NPC death.

### LIVE/DEAD assay

The LIVE/DEAD Viability/Cytotoxicity Kit for mammalian cells (Thermo Fisher Scientific, catalog no. L3224) was used to assess the viability of dispersed NPCs or NPreCs in MFD cultures (the “Dispersed NPCs or NPreCs in MFDs” section). For samples with NPCs, LIVE/DEAD staining was performed on the first and last days of cell culture, depending on the visible state of cells. For samples with NPreCs, staining was performed on day 1, day 7, and day 14. Briefly, all samples were washed three times with PBS. Next, NPCs and NPreCs were incubated in EGM-2:NPM-2 and EGM-2:NMM, respectively, with calcein (2 μM) and ethidium homodimer-1 (4 μM) for 30 min. Afterward, samples were rinsed three times with PBS and cultured in their respective culture medium. Fluorescence z-stack (150-μm range with 10-μm intervals) images of cells were acquired with an Eclipse Ti2 microscope with a 10× objective. To determine the percentage of living cells in each ROI, z-stacks were compressed to maximum intensity projections (MIPs) and analyzed in ImageJ (National Institutes of Health).

### Neurosphere generation and characterization

NPCs, NPCs-ZsG, and NPreCs were aggregated into neurospheres (NPC-Nsphs, NPC-ZsG-Nsphs, and NPreC-Nsphs, respectively) to facilitate the analysis of NPC expansion and NPreC neurogenesis in MFD culture (fig. S2A) According to the manufacturer’s instructions, cells were added to wells of AggreWell 400 Microwell Plates (Stemcell Technologies, catalog no. 34415) and centrifuged to promote neurosphere formation. Neurosphere cell density was varied to generate different sized neurospheres (100, 200, and 400 cells per Nsph) depending on experimental requirements. Neurospheres composed of NPCs or NPreCs were incubated in microwells with NPM-2 or NDM, respectively, for 24 hours. In a preliminary experiment, newly generated NPC-Nsphs and NPreC-Nsphs of all cell densities were characterized by measuring their diameters. Briefly, before removal from microwells, NPC-Nsphs and NPreC-Nsphs were incubated in NPM-2 and NDM, respectively, with 5 μM CellTracker Red CMTPX dye (Thermo Fisher Scientific, catalog no. C34552) for 1 hour. Afterward, the CellTracker Red solution was replaced with normal culture medium. All neurospheres were imaged in microwells with an Eclipse Ti2 inverted microscope (Nikon) with a 10× objective. In ImageJ, the CellTracker Red signal was used to calculate the area of each individual neurosphere. Neurosphere diameter was derived from this measurement by assuming that all neurospheres were circular.

### NPC neurosphere expansion in MFDs

Once NPC-ZsG-Nsphs were generated using the same protocol detailed in the “Neurosphere generation and characterization” section, we sought to determine how different culture factors would influence their growth. To test this, NPC-ZsG-Nsphs were seeded and cultured in MFDs using methods similar to those described in the “BMVN generation in MFDs” section. In a preliminary experiment, we investigated the effect of neurosphere density on neurosphere expansion. Briefly, NPC-ZsG-Nsphs with densities of either 100, 200, or 400 NPCs-ZsG per neurosphere (NPC-ZsG per Nsph) were resuspended alone in fibrin gels within the hydrogel channel of MFDs. In a follow-up experiment, we cultured NPC-ZsG-Nsphs with different combinations of supporting cells to improve neurosphere survival and expansion in MFDs. Briefly, in the hydrogel channel of MFDs, NPC-ZsG-Nsphs (200 NPC-ZsG per Nsph) were resuspended in fibrin hydrogels alone (NPC condition), with PCs (NPC-PC condition), with ACs (NPC-AC condition), with BECs-tdT (NPC-BEC condition), with BECs-tdT, PCs, and ACs (NPC-BEC-PC-AC condition), and with HUVECs-tdT, PCs, and ACs (NPC-HUVEC-PC-AC condition). The final concentrations of endothelial cells, PCs, and ACs in all cocultures were 6 × 10^6^, 2 × 10^6^, and 2 × 10^6^ cells/ml, respectively. Approximately 30 NPC-ZsG-Nsphs were present in each sample described in this section. In all experiments, samples were cultured in EGM-2:NPM-2 to promote the growth of all cell types. To generate IF across the hydrogel channel, all samples were cultured under flow conditions for 1 week.

### NPC neurosphere expansion analysis

The expansion of NPC-ZsG-Nsphs in MFDs was defined as the migration of cells out of neurospheres and into the surrounding fibrin ECM. To measure this, fluorescence z-stack (150-μm range with 5-μm intervals) images of individual NPC-ZsG-Nsphs were acquired on day 1 and day 7 of culture using an Eclipse Ti2 microscope with a 20× objective. In ImageJ, all z-stacks were compressed to MIPs. Neurosphere size was quantified by measuring the cumulative area of ZsGreen1 signal from NPC-ZsG-Nsphs. An increase in NPC-ZsG-Nsph area over time was referred to as neurosphere expansion and assumed to be indicative of NPC-ZsG survival and migration. A decrease in NPC-ZsG-Nsph area over time was referred to as neurosphere regression and assumed to be indicative of NPC-ZsG apoptosis. Neurosphere area ratio was calculated by dividing the final neurosphere area on day 7 by the original area on day 1. Using this method, the relative expansion of NPC-ZsG-Nsphs could be statistically compared between sample groups.

### Dispersed NPCs and BMVNs in MFDs

After it was confirmed that the presence of BMVNs was beneficial for NPC-ZsG-Nsph expansion in MFDs, we sought to determine the effect of IF on the survival of individual NPCs cocultured with BMVNs. To test this, BECs-tdT, PCs, ACs, and NPCs-ZsG were harvested and resuspended together in fibrin gels within MFD hydrogel channels using methods similar to those described in the “BMVN generation in MFDs” section. In the MFDs, the final concentration of BECs-tdT and all other cell types was 6 × 10^6^ and 2 × 10^6^ cells/ml, respectively. Samples were cultured under either flow or static conditions in EGM-2:NPM-2 for 1 week. Fluorescence z-stack (50-μm range with 5-μm intervals) images were acquired of BMVNs on day 7 using a LSM 800 confocal laser scanning microscope (Zeiss) with a 20× objective. Using ImageJ, the number of ZsGreen1^+^ nuclei per ROI was counted to determine the number of surviving NPCs-ZsG in flow and static samples. To compare the characteristics (vessel area, average vessel diameter, and the number of vessel segments) of BMVNs developed under flow and static conditions, microvessel analysis (section S1.5) was performed, as described in greater detail in our previous publication (*30*).

### NPreC neurosphere neurogenesis in MFDs

After NPreC-Nsphs were generated using the protocol detailed in the “Neurosphere generation and characterization” section, we sought to determine how different culture factors would influence NPreC neurogenesis. To test this, NPreC-Nsphs were seeded and cultured in MFDs using methods similar to those described in the “BMVN generation in MFDs” section. In one experiment, NPreC-Nsphs were cultured with different combinations of supporting cells to determine the neurotrophic effects of each cell type in BMVNs. Briefly, in the hydrogel channel of MFDs, NPreC-Nsphs (400 NPreCs per Nsph) were resuspended in fibrin hydrogels alone (NPreC condition), with PCs (NPreC-PC condition), with ACs (NPreC-AC condition), with BECs-tdT (NPreC-BEC condition), or with BECs-tdT, PCs, and ACs (NPreC-BEC-PC-AC condition). The final concentrations of BECs-tdT, PCs, and ACs in all cocultures were 6 × 10^6^, 2 × 10^6^, and 2 × 10^6^ cells/ml, respectively. Approximately 30 NPreC-Nsphs were present in each sample. All samples were cultured in EGM-2:NMM to promote the growth of all cell types. All cultures were grown under flow conditions for 10 days. In a separate experiment, we sought to determine the effect of IF on neurogenesis for NPreC-Nsphs cocultured with BMVNs. To test this, NPreC-BEC-PC-AC cultures were recreated in MFDs. Samples were then cultured under either flow or static conditions for 1 week. To compare the characteristics of BMVNs developed under flow and static cultures, microvessel analysis (section S1.5) was performed to quantify vessel area, average vessel diameter, and the number of vessel segments. Luminal flow through microvessels was visualized in samples cultured under flow conditions using a microsphere perfusion assay (section S1.1).

### NPreC neurosphere neurite analysis

After NPreC-Nsphs were cultured in MFDs, we measured neurosphere core area, total neurite outgrowth, and maximum neurite extension to determine the effect of culture conditions on neurogenesis. Briefly, all samples were fixed and labeled with Tuj1 and Hoechst. Next, fluorescence z-stack (100-μm range with 5-μm intervals) images of NPreC-Nsphs were acquired using a LSM 800 confocal microscope with a 20× objective. Neurospheres in close proximity to other neurospheres or microposts were not considered for analysis. In ImageJ, z-stacks were compressed to MIPs, and neurosphere perimeters were manually outlined by tracing the border of dense Hoechst and Tuj1 signals found near the center of neurospheres. Neurosphere core area was defined as the area within this perimeter. Total neurite outgrowth was defined as the summation of the length of all neurites that had extended from the neurosphere perimeter. To measure this, a binary image of the Tuj1 signal outside the neurosphere core area was generated. Next, particles with areas smaller than 9.61 μm^2^ or circularities larger than 0.5 were removed from binary images to eliminate any Tuj1 signals that were not associated with healthy, continuous neurites. Last, the plugins Skeletonize and Analyze Skeleton were used to determine the total length of all neurite branches for each neurosphere. Maximum neurite extension was measured as the Euclidean distance from the most distant neurite tip to the neurosphere perimeter.

### NPreC neurosphere calcium oscillation assay and analysis

After observing neuritogenesis from NPreC-Nsphs in MFDs, we sought to investigate the functionality of these NPC-neurons by measuring the oscillations of calcium ions (Ca^2+^) in cells. This was done by modifying the protocol detailed in the “NPreC neurosphere neurogenesis in MFDs” section. Briefly, before NPreC-Nsphs were removed from AggreWells, they were incubated in NDM with 10 μM CellTracker Red for 30 min. Next, NPreC-Nsphs (400 NPreCs per Nsph) were resuspended in fibrin hydrogels alone (NPreC condition) or with BECs, PCs and ACs (NPreC-BEC-PC-AC condition) in the hydrogel channel of MFDs. All samples were cultured in EGM-2:NMM under flow conditions for 10 days. To observe Ca^2+^ oscillations, all samples were first washed three times with PBS. Next, samples were incubated in BrainPhys Imaging Optimized Medium (BIOM; Stemcell Technologies, catalog no. 05796) with 2% NeuroCult SM1 Neuronal Supplement (Stemcell Technologies, catalog no. 05711) and 5 μM green fluorescent calcium binding dye, Fluo-8 AM (Abcam, catalog no. ab142773), for 2 hours. Afterward, all samples were rinsed with BIOM without Fluo-8 three times. Then, fluorescence time-lapse (5 min with 10-s intervals) images were acquired of individual neurospheres using a LSM 880 confocal laser scanning microscope (Zeiss) with a 20× objective at 37°C and 5% CO_2_. At each time point, a z-stack image was acquired to create a 10 μm “slice” of each neurosphere starting from the bottom of the hydrogel channel (2.5-μm interval). Calcium oscillation analysis was performed in ImageJ. For each neurosphere slice, individual NPC-neurons were identified by the overlap of CellTracker Red and Fluo-8 signal. Circular ROIs were then created to measure the change in Fluo-8 signal intensity over time in the cell bodies of NPC-neurons. Ca^2+^ oscillations were represented as the change in fluorescence intensity relative to the initial recorded value ($\frac{\text{Δ}F}{F_{0}}$). We defined a “spike” as a fluorescence intensity increase of 50% from *F*_0_ over any time frame. NPC-neurons that produced a spike during the 5-min recording period were considered “firing” NPC-neurons. To quantify Ca^2+^ oscillations in each neurosphere slice, we counted the number of firing NPC-neurons and measured the total number of spikes per minute.

### Dispersed NPreCs and BMVNs in MFDs

After we confirmed that BMVNs enhanced NPreC-Nsph neurogenesis in MFDs, we sought to determine the influence of IF on the self-renewal and differentiation on individual NPreCs. To test this, BMECs-tdT, PCs, ACs, and NPreCs-ZsG were harvested and resuspended together in fibrin gels within MFD hydrogel channels using methods similar to those described in the “BMVN generation in MFDs” section. BMECs-tdT were used instead of BECs-tdT due to a lack of availability of BECs-tdT at the time of experiment. The final concentrations of BMECs-tdT and all other cell types were 6 × 10^6^ and 2 × 10^6^ cells/ml, respectively. Samples were cultured under either flow or static conditions in EGM-2:NMM for 2 weeks. On day 14, samples were fixed and labeled for Sox2, MAP2, GFAP, and Hoechst. Fluorescence z-stack (50-μm range with 5-μm intervals) images were acquired of samples using a LSM 800 confocal microscope with a 20× objective. In ImageJ, each z-stack was converted to a MIP and the number of ZsGreen1^+^ NPreCs was counted. Next, the number of Sox2^+^/ZsGreen1^+^ and MAP2^+^/ZsGreen1^+^ cells were counted in samples stained for each protein. The number of Sox2^+^/ZsGreen1^+^ and MAP2^+^/ZsGreen1^+^ was represented as a percentage of the total number of ZsGreen1^+^ cells in each ROI. A list of all experimental conditions presented in this work can be found in table S3.

### Statistical analysis

Prism 9 (GraphPad Software) was used for all statistical analysis and graph generation. Two-tailed Welch’s *t* test was performed for all statistical comparisons between two experimental groups. One-way or Welch’s analysis of variance (ANOVA) was performed for comparisons between more than two experimental groups. Two-way ANOVA was performed for comparisons with two experimental variables. Post hoc analysis was performed using either Tukey’s, Dunnett’s, or Šidák’s multiple comparisons test, based on the method recommended by Prism 9. Statistical significance was defined as *P* < 0.05. The number of biological replicates used in each experiment is described in the figure captions. All data are represented as the means ± SEM unless otherwise specified.

## Notes

View/request a protocol for this paper from *Bio-protocol*.

## References

[1] C. L. Gooch, E. Pracht, A. R. Borenstein, The burden of neurological disease in the United States: A summary report and call to action. Ann. Neurol. 81, 479–484 (2017). 28198092 10.1002/ana.24897

[2] G.-L. Ming, H. Song, Adult neurogenesis in the mammalian brain: Significant answers and significant questions. Neuron 70, 687–702 (2011). 21609825 10.1016/j.neuron.2011.05.001 PMC3106107

[3] F. H. Gage, Mammalian neural stem cells. Science 287, 1433–1438 (2000). 10688783 10.1126/science.287.5457.1433

[4] S. U. Kim, H. J. Lee, Y. B. Kim, Neural stem cell-based treatment for neurodegenerative diseases. Neuropathology 33, 491–504 (2013). 23384285 10.1111/neup.12020

[5] M. Tavazoie, L. van der Veken, V. Silva-Vargas, M. Louissaint, L. Colonna, B. Zaidi, J. M. Garcia-Verdugo, F. Doetsch, A specialized vascular niche for adult neural stem cells. Cell Stem Cell 3, 279–288 (2008). 18786415 10.1016/j.stem.2008.07.025 PMC6864413

[6] J. S. Goldberg, K. K. Hirschi, "A Vascular Perspective on Neurogenesis" in *Neural Stem Cells - New Perspectives* (InTech, 2013; www.intechopen.com/books/neural-stem-cells-new-perspectives/a-vascular-perspective-on-neurogenesis), pp. 201–239.

[7] C. Lois, J. M. García-Verdugo, A. Alvarez-Buylla, Chain migration of neuronal precursors. Science 271, 978–981 (1996). 8584933 10.1126/science.271.5251.978

[8] M. A. Winkelman, A. N. Koppes, R. A. Koppes, G. Dai, Bioengineering the neurovascular niche to study the interaction of neural stem cells and endothelial cells. APL Bioeng. 5, 011507 (2021). 33688617 10.1063/5.0027211 PMC7932757

[9] C. Ottone, B. Krusche, A. Whitby, M. Clements, G. Quadrato, M. E. Pitulescu, R. H. Adams, S. Parrinello, Direct cell-cell contact with the vascular niche maintains quiescent neural stem cells. Nat. Cell Biol. 16, 1045–1056 (2014). 25283993 10.1038/ncb3045 PMC4298702

[10] C.-H. Chou, J. D. Sinden, P.-O. Couraud, M. Modo, J. Schneider, In vitro modeling of the neurovascular environment by coculturing adult human brain endothelial cells with human neural stem cells. PLOS ONE 9, e106346 (2014). 25187991 10.1371/journal.pone.0106346 PMC4154686

[11] Q. Shen, S. K. Goderie, L. Jin, N. Karanth, Y. Sun, N. Abramova, P. Vincent, K. Pumiglia, S. Temple, Endothelial cells stimulate self-renewal and expand neurogenesis of neural stem cells. Science 304, 1338–1340 (2004). 15060285 10.1126/science.1095505

[12] M. C. Ford, J. P. Bertram, S. Royce Hynes, M. Michaud, Q. Li, M. Young, S. S. Segal, J. A. Madri, E. B. Lavik, A macroporous hydrogel for the coculture of neural progenitor and endothelial cells to form functional vascular networks in vivo. Proc. Natl. Acad. Sci. U.S.A. 103, 2512–2517 (2006). 16473951 10.1073/pnas.0506020102 PMC1413771

[13] J. Arulmoli, H. J. Wright, D. T. T. Phan, U. Sheth, R. A. Que, G. A. Botten, M. Keating, E. L. Botvinick, M. M. Pathak, T. I. Zarembinski, D. S. Yanni, O. V. Razorenova, C. C. W. Hughes, L. A. Flanagan, Combination scaffolds of salmon fibrin, hyaluronic acid, and laminin for human neural stem cell and vascular tissue engineering. Acta Biomater. 43, 122–138 (2016). 27475528 10.1016/j.actbio.2016.07.043 PMC5386322

[14] H. Wang, H. Yang, Y. Shi, Y. Xiao, Y. Yin, B. Jiang, H. Ren, W. Chen, Q. Xue, X. Xu, Reconstituting neurovascular unit with primary neural stem cells and brain microvascular endothelial cells in three-dimensional matrix. Brain Pathol. 31, e12940 (2021). 33576166 10.1111/bpa.12940 PMC8412118

[15] K. Yang, J. S. Lee, S. Han, Y. Jin, A.-N. Cho, G.-E. Chang, E. Cheong, J. H. Yang, S. Chung, S.-W. Cho, Endothelial-neurosphere crosstalk in microwell arrays regulates self-renewal and differentiation of human neural stem cells. J. Ind. Eng. Chem. 74, 148–157 (2019).

[16] H.-W. W. Han, Y.-T. Te Hou, S.-H. Hsu, Angiogenic potential of co-spheroids of neural stem cells and endothelial cells in injectable gelatin-based hydrogel. Mater. Sci. Eng. C 99, 140–149 (2019). 10.1016/j.msec.2019.01.089 30889675

[17] L. Song, X. Yuan, Z. Jones, K. Griffin, Y. Zhou, T. Ma, Y. Li, Assembly of human stem cell-derived cortical spheroids and vascular spheroids to model 3-d brain-like tissues. Sci. Rep. 9, 5977 (2019). 30979929 10.1038/s41598-019-42439-9 PMC6461701

[18] A. K. Shetty, G. Zanirati, The interstitial system of the brain in health and disease. Aging Dis. 11, 200–211 (2020). 32010493 10.14336/AD.2020.0103 PMC6961771

[19] Z. D. Shi, X. Y. Ji, D. E. Berardi, H. Qazi, J. M. Tarbell, Interstitial flow induces MMP-1 expression and vascular SMC migration in collagen I gels via an ERK1/2-dependent and c-Jun-mediated mechanism. Am. J. Physiol. Heart Circ. Physiol. 298, H127–H135 (2010). 19880665 10.1152/ajpheart.00732.2009 PMC2806139

[20] Y. Abe, M. Watanabe, S. Chung, R. D. Kamm, K. Tanishita, R. Sudo, Balance of interstitial flow magnitude and vascular endothelial growth factor concentration modulates three-dimensional microvascular network formation. APL Bioeng. 3, 036102 (2019). 31431938 10.1063/1.5094735 PMC6697031

[21] L. F. Alonzo, M. L. Moya, V. S. Shirure, S. C. George, Microfluidic device to control interstitial flow-mediated homotypic and heterotypic cellular communication. Lab Chip 15, 3521–3529 (2015). 26190172 10.1039/c5lc00507h PMC4855298

[22] T. M. Tinken, D. H. J. Thijssen, N. Hopkins, M. A. Black, E. A. Dawson, C. T. Minson, S. C. Newcomer, M. H. Laughlin, N. T. Cable, D. J. Green, Impact of shear rate modulation on vascular function in humans. Hypertension 54, 278–285 (2009). 19546374 10.1161/HYPERTENSIONAHA.109.134361 PMC3012006

[23] H. Kadry, B. Noorani, L. Cucullo, A blood–brain barrier overview on structure, function, impairment, and biomarkers of integrity. Fluids Barriers CNS 17, 69 (2020). 33208141 10.1186/s12987-020-00230-3 PMC7672931

[24] Y. Shin, K. Yang, S. Han, H. J. Park, Y. S. Heo, S. W. Cho, S. Chung, Reconstituting vascular microenvironment of neural stem cell niche in three-dimensional extracellular matrix. Adv. Healthc. Mater. 3, 1457–1464 (2014). 24523050 10.1002/adhm.201300569

[25] S. Sances, R. Ho, G. Vatine, D. West, A. Laperle, A. Meyer, M. Godoy, P. S. Kay, B. Mandefro, S. Hatata, C. Hinojosa, N. Wen, D. Sareen, G. A. Hamilton, C. N. Svendsen, Human iPSC-derived endothelial cells and microengineered organ-chip enhance neuronal development. Stem Cell Rep. 10, 1222–1236 (2018). 10.1016/j.stemcr.2018.02.012 PMC5998748 29576540

[26] H. Uwamori, T. Higuchi, K. Arai, R. Sudo, Integration of neurogenesis and angiogenesis models for constructing a neurovascular tissue. Sci. Rep. 7, 17349 (2017). 29229920 10.1038/s41598-017-17411-0 PMC5725567

[27] G. Kaushik, K. Gupta, V. Harms, E. Torr, J. Evans, H. J. Johnson, C. Soref, S. Acevedo-Acevedo, J. Antosiewicz-Bourget, D. Mamott, P. Uhl, B. P. Johnson, S. P. Palecek, D. J. Beebe, J. A. Thomson, W. T. Daly, W. L. Murphy, Engineered perineural vascular plexus for modeling developmental toxicity. Adv. Healthc. Mater. 9, 2000825 (2020). 10.1002/adhm.202000825 PMC8016604 32613760

[28] T. Osaki, V. Sivathanu, R. D. Kamm, Engineered 3D vascular and neuronal networks in a microfluidic platform. Sci. Rep. 8, 5168 (2018). 29581463 10.1038/s41598-018-23512-1 PMC5979969

[29] N. Shin, Y. Kim, J. Ko, S. W. Choi, S. Hyung, S. E. Lee, S. Park, J. Song, N. L. Jeon, K. S. Kang, Vascularization of iNSC spheroid in a 3D spheroid-on-a-chip platform enhances neural maturation. Biotechnol. Bioeng. 119, 566–574 (2022). 34716703 10.1002/bit.27978 PMC9298365

[30] M. A. Winkelman, D. Y. Kim, S. Kakarla, A. Grath, N. Silvia, G. Dai, Interstitial flow enhances the formation, connectivity, and function of 3D brain microvascular networks generated within a microfluidic device. Lab Chip 22, 170–192 (2021). 34881385 10.1039/d1lc00605c PMC9257897

[31] N. E. Baker, Emerging mechanisms of cell competition. Nat. Rev. Genet. 21, 683–697 (2020). 32778819 10.1038/s41576-020-0262-8 PMC8205513

[32] P. Codega, V. Silva-Vargas, A. Paul, A. R. Maldonado-Soto, A. M. DeLeo, E. Pastrana, F. Doetsch, Prospective identification and purification of quiescent adult neural stem cells from their in vivo niche. Neuron 82, 545–559 (2014). 24811379 10.1016/j.neuron.2014.02.039 PMC4360885

[33] B. Menn, J. M. Garcia-Verdugo, C. Yaschine, O. Gonzalez-Perez, D. Rowitch, A. Alvarez-Buylla, Origin of oligodendrocytes in the subventricular zone of the adult brain. J. Neurosci. 26, 7907–7918 (2006). 16870736 10.1523/JNEUROSCI.1299-06.2006 PMC6674207

[34] M. A. Bonaguidi, M. A. Wheeler, J. S. Shapiro, R. P. Stadel, G. J. Sun, G. Ming, H. Song, In vivo clonal analysis reveals self-renewing and multipotent adult neural stem cell characteristics. Cell 145, 1142–1155 (2011). 21664664 10.1016/j.cell.2011.05.024 PMC3124562

[35] H. Mori, K. Ninomiya, M. Kino-oka, T. Shofuda, M. O. Islam, M. Yamasaki, H. Okano, M. Taya, Y. Kanemura, Effect of neurosphere size on the growth rate of human neural stem/progenitor cells. J. Neurosci. Res. 84, 1682–1691 (2006). 17044035 10.1002/jnr.21082

[36] D. Ge, K. Song, S. Guan, M. Dai, X. Ma, T. Liu, Effect of the neurosphere size on the viability and metabolism of neural stem/progenitor cells. Afr. J. Biotechnol. 11, 3976–3985 (2012).

[37] P. Lu, L. Graham, Y. Wang, D. Wu, M. Tuszynski, Promotion of survival and differentiation of neural stem cells with fibrin and growth factor cocktails after severe spinal cord injury. J. Vis. Exp., e50641 (2014). 25145787 10.3791/50641 PMC4435462

[38] A. R. Bento, P. Quelhas, M. J. Oliveira, A. P. Pêgo, I. F. Amaral, Three-dimensional culture of single embryonic stem-derived neural/stem progenitor cells in fibrin hydrogels: Neuronal network formation and matrix remodelling. J. Tissue Eng. Regen. Med. 11, 3494–3507 (2017). 28032468 10.1002/term.2262

[39] S. Tara, L. K. Krishnan, Differentiation of circulating neural progenitor cells in vitro on fibrin-based composite -matrix involves Wnt- β-catenin-like signaling. J. Cell Commun. Signal. 13, 27–38 (2018). 29856041 10.1007/s12079-018-0467-1 PMC6381381

[40] A. C. Delgado, S. R. Ferró, D. Vicente, E. Porlan, A. Perez-Villalba, C. M. Trujillo, I. Fariñas, Endothelial NT-3 delivered by vasculature and CSF promotes quiescence of subependymal neural stem cells through nitric oxide induction. Neuron 83, 572–585 (2014). 25043422 10.1016/j.neuron.2014.06.015

[41] S. Baratchi, J. Evans, W. P. Tate, W. C. Abraham, B. Connor, Secreted amyloid precursor proteins promote proliferation and glial differentiation of adult hippocampal neural progenitor cells. Hippocampus 22, 1517–1527 (2012). 22147523 10.1002/hipo.20988

[42] I. Kazanis, J. D. Lathia, T. J. Vadakkan, E. Raborn, R. Wan, M. R. Mughal, D. M. Eckley, T. Sasaki, B. Patton, M. P. Mattson, K. K. Hirschi, M. E. Dickinson, C. ffrench-Constant, Quiescence and activation of stem and precursor cell populations in the subependymal zone of the mammalian brain are associated with distinct cellular and extracellular matrix signals. J. Neurosci. 30, 9771–9781 (2010). 20660259 10.1523/JNEUROSCI.0700-10.2010 PMC3842479

[43] J. Sun, W. Zhou, D. Ma, Y. Yang, Endothelial cells promote neural stem cell proliferation and differentiation associated with VEGF activated Notch and Pten signaling. Dev. Dyn. 239, 2345–2353 (2010). 20730910 10.1002/dvdy.22377

[44] M. V. Gómez-Gaviro, C. E. Scott, A. K. Sesay, A. Matheu, S. Booth, C. Galichet, R. Lovell-Badge, Betacellulin promotes cell proliferation in the neural stem cell niche and stimulates neurogenesis. Proc. Natl. Acad. Sci. U.S.A. 109, 1317–1322 (2012). 22232668 10.1073/pnas.1016199109 PMC3268286

[45] M. Snapyan, M. Lemasson, M. S. Brill, M. Blais, M. Massouh, J. Ninkovic, C. Gravel, F. Berthod, M. Götz, P. A. Barker, A. Parent, A. Saghatelyan, Vasculature guides migrating neuronal precursors in the adult mammalian forebrain via brain-derived neurotrophic factor signaling. J. Neurosci. 29, 4172–4188 (2009). 19339612 10.1523/JNEUROSCI.4956-08.2009 PMC6665362

[46] E. Kokovay, S. Goderie, Y. Wang, S. Lotz, G. Lin, Y. Sun, B. Roysam, Q. Shen, S. Temple, Adult SVZ lineage cells home to and leave the vascular niche via differential responses to SDF1/CXCR4 signaling. Cell Stem Cell 7, 163–173 (2010). 20682445 10.1016/j.stem.2010.05.019 PMC2916873

[47] L. Wang, Z. G. Zhang, R. L. Zhang, S. R. Gregg, A. Hozeska-Solgot, Y. Letourneau, Y. Wang, M. Chopp, Matrix metalloproteinase 2 (MMP2) and MMP9 secreted by erythropoietin-activated endothelial cells promote neural progenitor cell migration. J. Neurosci. 26, 5996–6003 (2006). 16738242 10.1523/JNEUROSCI.5380-05.2006 PMC6675216

[48] L. S. Brown, C. G. Foster, J.-M. Courtney, N. E. King, D. W. Howells, B. A. Sutherland, Pericytes and neurovascular function in the healthy and diseased brain. Front. Cell. Neurosci. 13, 282 (2019). 31316352 10.3389/fncel.2019.00282 PMC6611154

[49] A. A. Galiakberova, E. B. Dashinimaev, Neural stem cells and methods for their generation from induced pluripotent stem cells in vitro. Front. Cell Dev. Biol. 8, 815 (2020). 33117792 10.3389/fcell.2020.00815 PMC7578226

[50] D. C. Darland, L. J. Massingham, S. R. Smith, E. Piek, M. Saint-Geniez, P. A. D’Amore, Pericyte production of cell-associated VEGF is differentiation-dependent and is associated with endothelial survival. Dev. Biol. 264, 275–288 (2003). 14623248 10.1016/j.ydbio.2003.08.015

[51] F.-W. Wang, H.-B. Hao, S.-D. Zhao, Y.-M. Zhang, Q. Liu, H.-j. Liu, S.-M. Liu, Q.-H. Yuan, L.-J. Bing, E.-A. Ling, A.-J. Hao, Roles of activated astrocyte in neural stem cell proliferation and differentiation. Stem Cell Res. 7, 41–53 (2011). 21530437 10.1016/j.scr.2011.03.004

[52] J. Jiao, D. F. Chen, Induction of neurogenesis in nonconventional neurogenic regions of the adult central nervous system by niche astrocyte-produced signals. Stem Cells 26, 1221–1230 (2008). 18323412 10.1634/stemcells.2007-0513 PMC2683670

[53] L. Ray, J. J. Iliff, J. J. Heys, Analysis of convective and diffusive transport in the brain interstitium. Fluids Barriers CNS 16, 1–18 (2019). 30836968 10.1186/s12987-019-0126-9 PMC6402182

[54] M. Campisi, Y. Shin, T. Osaki, C. Hajal, V. Chiono, R. D. Kamm, 3D self-organized microvascular model of the human blood-brain barrier with endothelial cells, pericytes and astrocytes. Biomaterials 180, 117–129 (2018). 30032046 10.1016/j.biomaterials.2018.07.014 PMC6201194

[55] A. Karakatsani, B. Shah, C. Ruiz de Almodovar, *Blood vessels as regulators of neural stem cell properties* (Frontiers Media S.A., 2019), vol. 12. 10.3389/fnmol.2019.00085 PMC6473036 31031591

[56] H. H. Tung, S. L. Lee, Physical binding of endothelial MCAM and neural transmembrane protease matriptase - novel cell adhesion in neural stem cell vascular niche. Sci. Rep. 7, 4946 (2017). 28694515 10.1038/s41598-017-05131-4 PMC5504030

[57] Q. Wei, V. Hariharan, H. Huang, Cell-cell contact preserves cell viability via plakoglobin. PLOS ONE 6, e27064 (2011). 22046445 10.1371/journal.pone.0027064 PMC3203941

[58] X. Li, X. Liu, Y. Tan, V. Tran, N. Zhang, X. Wen, Improve the viability of transplanted neural cells with appropriate sized neurospheres coated with mesenchymal stem cells. Med. Hypotheses 79, 274–277 (2012). 22657917 10.1016/j.mehy.2012.05.010

[59] B. W. Dulken, D. S. Leeman, S. C. Boutet, K. Hebestreit, A. Brunet, Single-cell transcriptomic analysis defines heterogeneity and transcriptional dynamics in the adult neural stem cell lineage. Cell Rep. 18, 777–790 (2017). 28099854 10.1016/j.celrep.2016.12.060 PMC5269583

[60] F. Ehret, S. Vogler, G. Kempermann, A co-culture model of the hippocampal neurogenic niche reveals differential effects of astrocytes, endothelial cells and pericytes on proliferation and differentiation of adult murine precursor cells. Stem Cell Res. 15, 514–521 (2015). 26448270 10.1016/j.scr.2015.09.010

[61] M. D. Sweeney, S. Ayyadurai, B. V. Zlokovic, Pericytes of the neurovascular unit: Key functions and signaling pathways. Nat. Neurosci. 19, 771–783 (2016). 27227366 10.1038/nn.4288 PMC5745011

[62] N. J. Abbott, L. Rönnbäck, E. Hansson, Astrocyte–Endothelial interactions at the blood–Brain barrier. Nat. Rev. Neurosci. 7, 41–53 (2006). 16371949 10.1038/nrn1824

[63] Y. Kim, J. Park, Y. K. Choi, The role of astrocytes in the central nervous system focused on BK channel and heme oxygenase metabolites: A review. Antioxidants 8, 121 (2019). 31060341 10.3390/antiox8050121 PMC6562853

[64] A. Louissaint, S. Rao, C. Leventhal, S. A. Goldman, Coordinated interaction of neurogenesis and angiogenesis in the adult songbird brain. Neuron 34, 945–960 (2002). 12086642 10.1016/s0896-6273(02)00722-5

[65] E. A. Winkler, R. D. Bell, B. V. Zlokovic, Central nervous system pericytes in health and disease. Nat. Neurosci. 14, 1398–1405 (2011). 22030551 10.1038/nn.2946 PMC4020628

[66] M. K. Rasmussen, H. Mestre, M. Nedergaard, The glymphatic pathway in neurological disorders. Lancet Neurol. 17, 1016–1024 (2018). 30353860 10.1016/S1474-4422(18)30318-1 PMC6261373

[67] M. A. Swartz, M. E. Fleury, Interstitial flow and its effects in soft tissues. Annu. Rev. Biomed. Eng. 9, 229–256 (2007). 17459001 10.1146/annurev.bioeng.9.060906.151850

[68] K. M. Kingsmore, A. Vaccari, D. Abler, S. X. Cui, F. H. Epstein, R. C. Rockne, S. T. Acton, J. M. Munson, MRI analysis to map interstitial flow in the brain tumor microenvironment. APL Bioeng. 2, 031905 (2018). 30456343 10.1063/1.5023503 PMC6238644

[69] C. M. Dumont, J. Piselli, S. Temple, G. Dai, D. M. Thompson, Endothelial cells exposed to fluid shear stress support diffusion based maturation of adult neural progenitor cells. Cell Mol. Bioeng. 11, 117–130 (2018). 31719881 10.1007/s12195-017-0516-5 PMC6816782

[70] J. Y. Park, J. B. White, N. Walker, C. H. Kuo, W. Cha, M. E. Meyerhoff, S. Takayama, Responses of endothelial cells to extremely slow flows. Biomicrofluidics 5, 22211 (2011). 21799717 10.1063/1.3576932 PMC3145236

[71] B. J. Ballermann, A. Dardik, E. Eng, A. Liu, "Shear stress and the endothelium" in *Kidney International, Supplement* (Nature Publishing Group, 1998), vol. 54, pp. S100–S108. 10.1046/j.1523-1755.1998.06720.x 9736263

[72] R. A. de Melo Reis, H. R. Freitas, F. G. de Mello, Cell calcium imaging as a reliable method to study neuron–Glial circuits. Front. Neurosci. 14, 569361 (2020). 33122991 10.3389/fnins.2020.569361 PMC7566175

[73] K.-W. Wu, J.-L. Mo, Z.-W. Kou, Q. Liu, L.-L. Lv, Y. Lei, F.-Y. Sun, Neurovascular interaction promotes the morphological and functional maturation of cortical neurons. Front. Cell. Neurosci. 11, 290 (2017). 28966577 10.3389/fncel.2017.00290 PMC5605567

[74] M. C. Whitman, W. Fan, L. Rela, D. J. Rodriguez-Gil, C. A. Greer, Blood vessels form a migratory scaffold in the rostral migratory stream. J. Comp. Neurol. 516, 94–104 (2009). 19575445 10.1002/cne.22093 PMC2746017

[75] D. R. Kornack, P. Rakic, The generation, migration, and differentiation of olfactory neurons in the adult primate brain. Proc. Natl. Acad. Sci. U.S.A. 98, 4752–4757 (2001). 11296302 10.1073/pnas.081074998 PMC31906

[76] J. M. Frade, M. C. Ovejero-Benito, Neuronal cell cycle: The neuron itself and its circumstances. Cell Cycle 14, 712–720 (2015). 25590687 10.1080/15384101.2015.1004937 PMC4418291

[77] D. Ribatti, B. Nico, E. Crivellato, The role of pericytes in angiogenesis. Int. J. Dev. Biol. 55, 261–268 (2011). 21710434 10.1387/ijdb.103167dr

[78] M. R. Williamson, C. J. A. Fuertes, A. K. Dunn, M. R. Drew, T. A. Jones, Reactive astrocytes facilitate vascular repair and remodeling after stroke. Cell Rep. 35, 109048 (2021). 33910014 10.1016/j.celrep.2021.109048 PMC8142687

[79] S. L. Fink, B. T. Cookson, Apoptosis, pyroptosis, and necrosis: Mechanistic description of dead and dying eukaryotic cells. Infect. Immun. 73, 1907–1916 (2005). 15784530 10.1128/IAI.73.4.1907-1916.2005 PMC1087413

[80] J. J. Ohab, S. Fleming, A. Blesch, S. T. Carmichael, A neurovascular niche for neurogenesis after stroke. J. Neurosci. 26, 13007–13016 (2006). 17167090 10.1523/JNEUROSCI.4323-06.2006 PMC6674957

