# Supplementary material for: Bioengineered perfused human brain microvascular networks enhance neural progenitor cell survival, neurogenesis, and maturation
Source: Sci Adv. 2023 May 10;9(19):eaaz9499. doi: 10.1126/sciadv.aaz9499 (PMC10171804; doi:10.1126/sciadv.aaz9499)
Supplement: Supplementary file 1 — Supplementary Text Figs. S1 to S9 Tables S1 to S3 Legend for movie S1 [file sciadv.aaz9499_sm.pdf]

Science Advances
AAAS

Supplementary Materials for

Bioengineered perfused human brain microvascular networks enhance neural progenitor cell survival, neurogenesis and maturation

Max A. Winkelman and Guohao Dai

Corresponding author: Guohao Dai, g.dai@northeastern.edu

Sci. Adv. **9**, eaaz9499 (2023)
DOI: 10.1126/sciadv.aaz9499

The PDF file includes:

Supplementary Text
Figs. S1 to S9
Tables S1 to S3
Legend for movie S1

Other Supplementary Material for this manuscript includes the following:

Movie S1

Supplementary Materials for "Bioengineered Perfused Human Brain Microvascular Networks Enhance Neural Progenitor Cell Survival, Neurogenesis and Maturation"

S1. Supplementary Methods

S1.1 Microsphere Perfusion Assay

After BMVNs were formed in MFDs using methods described in Sections 4.3 and 4.11, anastomosis was assessed using Dragon Green^{TM} polystyrene microspheres (1.9 μm diameter, Bang Laboratories, Cat: FSDG005). Briefly, microspheres were resuspended in EGM-2 at a ratio of 1:1000. For all samples, 50 μL of unadulterated EGM-2 were added to both reservoirs on one side of the hydrogel channel and 70 μL of microsphere solution were added to the remaining two reservoirs. Immediately afterwards, fluorescence timelapse images (15 or 30 seconds with 150 millisecond intervals) were acquired using an Eclipse Ti2 microscope with a 10X objective. Timelapse images were represented as MIPs.

S1.2 Dextran Perfusion Assay

After BMVNs were formed in MFDs using methods described in Section 4.3, microvessel permeability was assessed using fluorescent dextran. Briefly, Oregon Green^{TM} 488 dextran (70 kDa, Invitrogen, Cat: D7173) was dissolved in EGM-2 at a concentration of 5 μg/mL. For all samples, 50 μL of unadulterated EGM-2 were added to both reservoirs on one side of the hydrogel channel and 70 μL of dextran solution were added to the remaining two reservoirs. Immediately afterwards, fluorescence timelapse images (120 seconds with 15 second intervals) were acquired using an Eclipse Ti2 microscope with a 10X objective.

S1.3 BEC Angiogenesis Assay

To characterize the angiogenic effect of NPCs and NPreCs, we investigated their influence on BEC angiogenesis in MFDs. BECs were stimulated to produce angiogenic sprouts into fibrin matrices without cells (BEC condition), with PCs (BEC-PC condition), with ACs (BEC-AC condition), with NPCs (BEC-NPC conditions), and with NPreCs (BEC-NPreC condition). Briefly, using methods similar to those in Section 4.3, PCs, ACs, NPCs, and NPreCs were resuspended separately in fibrin gels within hydrogel channels at a density of 2×10^6 cells/mL. For BEC conditions, MFDs were injected with fibrin gels containing no supporting cells. Next, BECs-tdT or BECs-EGFP were resuspended in EGM-2 at a density of 5×10^6 cells/mL. Solutions of BECs-

tdT and BECs-EGFP were used in experiments to investigate the angiogenic effect of NPCs and NPreCs, respectively. For all samples, BEC solutions were added to one fluidic channel and MFDs were immediately incubated at a 90° angle for 15 minutes to promote BEC adherence to the fibrin gel. Afterwards, nonadherent BECs were washed away with PBS. Samples with BECs-tdT and BECs-EGFP were cultured in EGM-2:NPM-2 and EGM-2:NMM, respectively, under flow conditions for 7 days. On Day 1, Day 3, and Day 7, fluorescence z-stack (200 µm range with 10 µm intervals) images of angiogenic sprouts were acquired using an Eclipse Ti2 microscope with a 20X objective. In ImageJ, z-stacks were compressed to MIPs and ROIs were selected to include all angiogenic sprouts originating from between four adjacent microposts. BECs were identified by either tdTomato or EGFP signals. At each time point, the number of angiogenic sprouts in each ROI was counted. Sprout length was measured as the distance between the hydrogel-liquid interface and the tip of each sprout. This value was averaged between all sprouts in each ROI.

S1.4 BEC Vasculogenesis Assay

To characterize the vasculogenic effect of NPCs and NPreCs, we investigated their influence on BEC vasculogenesis in MFDs. BECs were stimulated to form vascular networks in fibrin gels with different supporting cells using methods similar to those in Section 4.3. Briefly, BECs-tdT or BECs-EGFP were resuspended in fibrin alone (BEC condition), with PCs (BEC-PC condition), with ACs (BEC-AC condition), with NPCs (BEC-NPC condition), and with NPreCs (BEC-NPreC condition) within hydrogel channels. Samples with BECs-tdT and BECs-EGFP were used in experiments to study NPCs and NPreCs, respectively. The final cell density of both BECs-tdT and BECs-EGFP was 6×10^6 cells/mL. The final cell density of PCs, ACs, NPCs, and NPreCs was 2×10^6 cells/mL. Samples with BECs-tdT and BECs-EGFP were cultured in EGM-2:NPM-2 and EGM-2:NMM, respectively, under flow conditions for 7 days. On Day 7, fluorescence z-stack (200 µm range with 10 µm intervals) images of vascular networks were acquired using an Eclipse Ti2 microscope with a 10X objective.

S1.5 Microvessel Analysis

To quantify and compare BMVN characteristics, microvessel analysis was performed. Briefly, fluorescence z-stack images were acquired from samples containing BMVNs using either an Eclipse Ti2 microscope or a LSM 800 confocal microscope. Z-stacks were then converted to

MIPs in ImageJ. EGFP or tdTomato signals were used to identify microvessels in images. Blood vessel area was measured as the area of microvessels within a given ROI and represented as a percentage of the total area. Using Skeletonize and Analyze Skeleton in ImageJ, average branch diameter was calculated using the following equation:

$$D_B = \frac{A_V}{n_B \times L_B} \qquad \textbf{(Eq. S1)}$$

where A_V is blood vessel area, n_B is the number of vessel branches, and L_B is the average vessel branch length. We also measured the number of vessel segments, which was defined as the number of continuous, interconnected microvessels in each ROI.

S2. Supplementary Figures

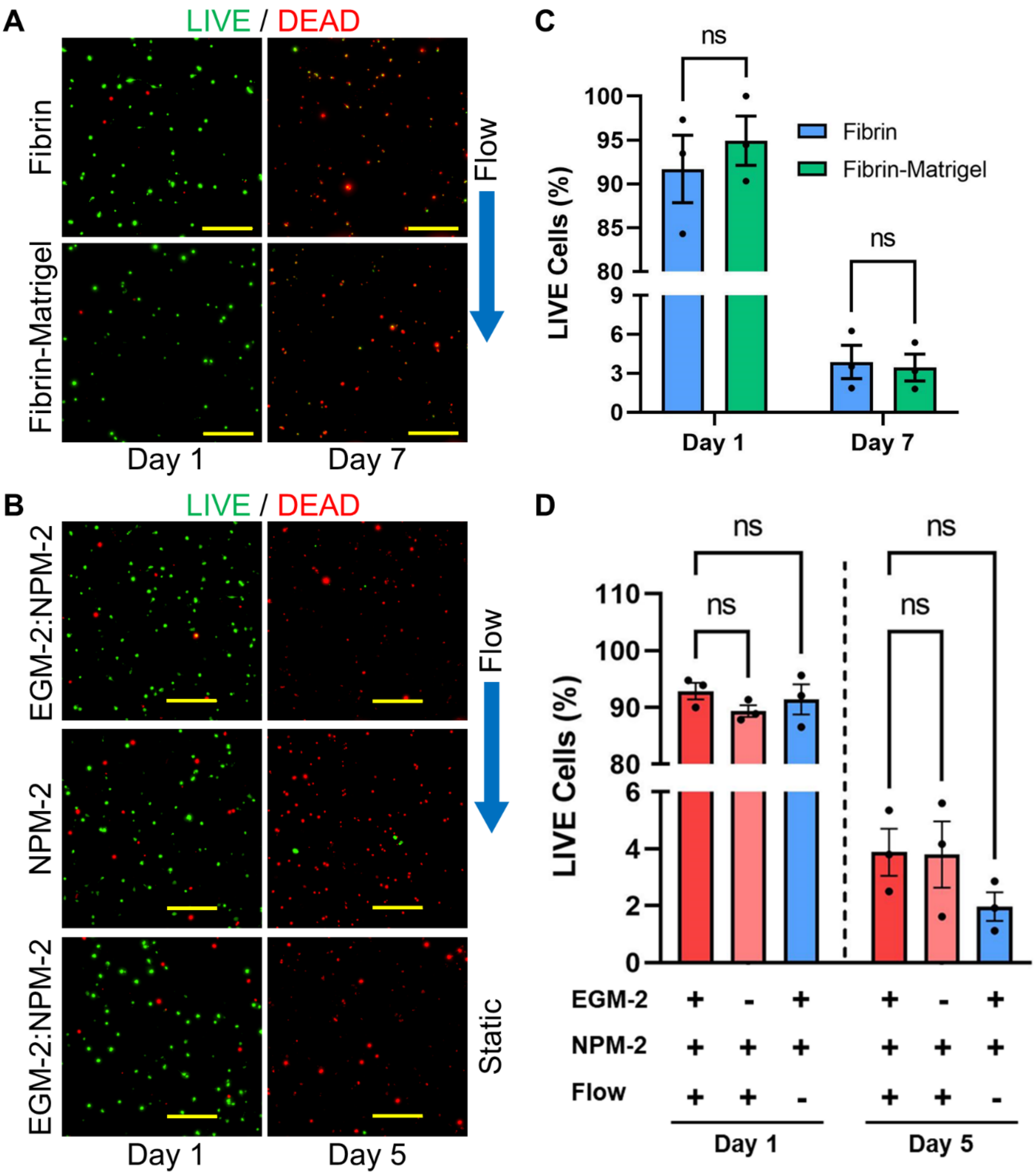

Figure S1. Viability of dispersed neural progenitor cells (NPCs) in MFD culture. A,B) LIVE (green) / DEAD (red) MIPs of NPCs cultured under various conditions in MFDs. **A)** To identify the influence of ECM on cell viability, NPCs (2×10^6 NPCs/mL) were resuspended in fibrin or fibrin-Matrigel hydrogels and imaged on Day 1 and Day 7. **B)** To identify the influence of interstitial flow and culture medium on cell viability, NPCs (2×10^6 NPCs/mL) were cultured in fibrin gels under flow or static conditions in either EGM-2:NPM-2 or NPM-2 and imaged on Day 1 and Day 5. Scale bars indicate 200 µm. Blue arrows indicate the direction of interstitial flow when applied. **C,D)** Graph of the corresponding percentage of LIVE NPCs cultured in different ECMs **(C)** and interstitial flow and cell medium conditions **(D)**. The data show mean value, error bars ± SEM, $n = 3$, two-way ANOVA with Šidák's **(C)** and Dunnett's **(D)** test, ns $p > 0.05$.

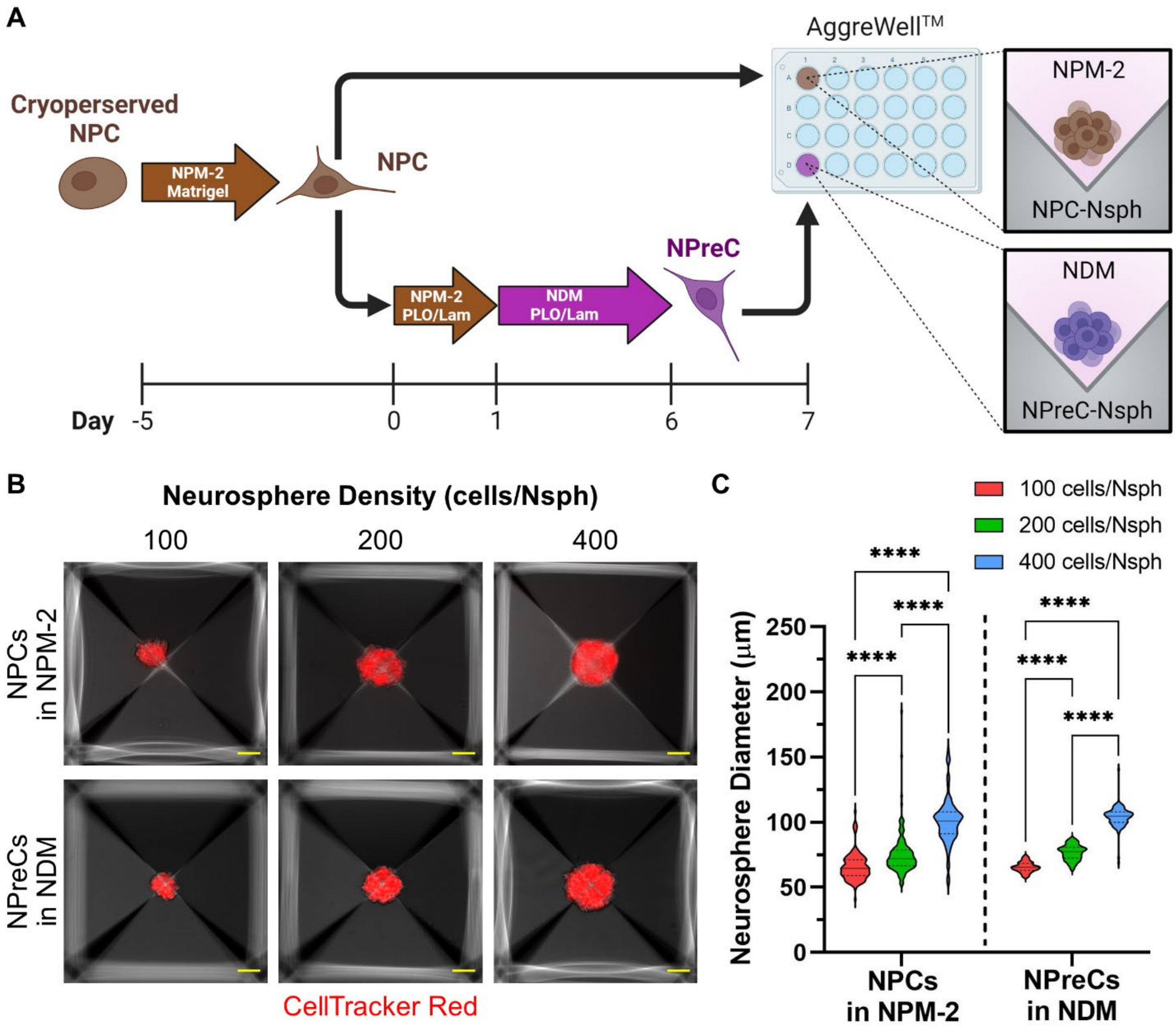

Figure S2. Neural progenitor cell (NPC) and neuronal precursor cell (NPreC) neurosphere generation. A) Diagram of NPC and NPreC culture and neurosphere generation. Culture dishes were coated with Matrigel or Poly-L-Ornithine and Laminin (PLO/Lam) where indicated. NPC neurospheres (NPC-Nsphs) and NPreC neurospheres (NPreC-Nsphs) were aggregated in AggreWell^{TM} Microwell Plates for 24 hours in NPM-2 and neuron differentiation medium (NDM), respectively, according to the manufacturer's instructions. **B)** Phase/fluorescence images of NPC-Nsphs and NPreC-Nsphs formed in microwells at densities of 100, 200, and 400 cells per neurosphere (cells/Nsph). Both cell types were labeled with CellTracker Red (red) to assist in diameter measurement. Scale bars indicate 50 μm. **C)** Violin plot of the diameters measured for NPC-Nsphs and NPreC-Nsphs with densities of 100, 200, and 400 cells/Nsph. The plot shows the empirical distribution of the data from the minimum to the maximum value. Solid lines indicate median value. N > 104 neurospheres were measured from same microwell in each condition, two-way ANOVA with Šidák's test, **** $p < 0.0001$.

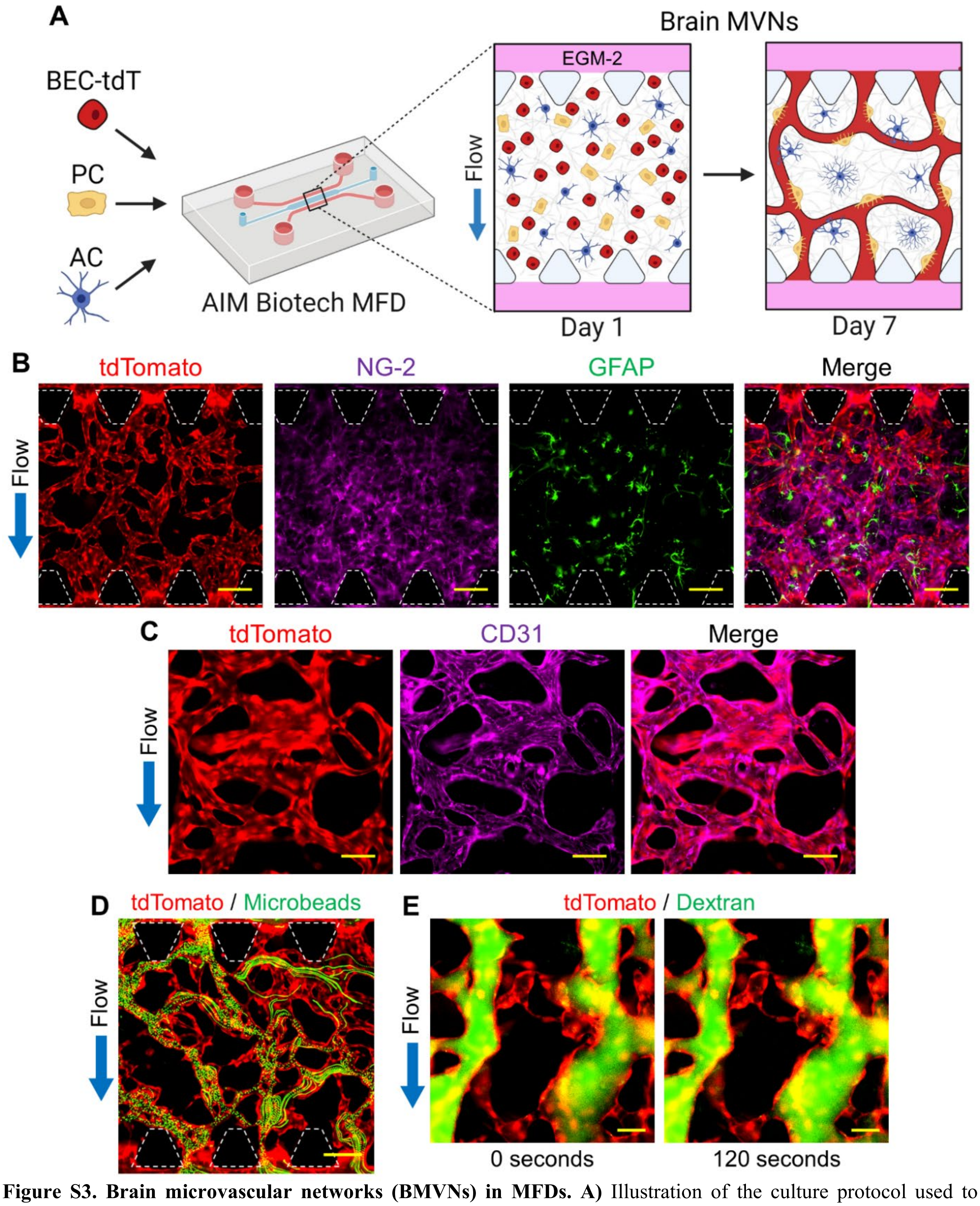

Figure S3. Brain microvascular networks (BMVNs) in MFDs. A) Illustration of the culture protocol used to develop BMVNs. Briefly, BECs-tdT, PCs, and ACs were resuspended in fibrin within MFDs and cultured in EGM-2 under flow conditions for one week. **B)** Fluorescence image of BMVN comprised of BECs (tdTomato, red), PCs (NG-2, purple), and ACs (GFAP, green) on Day 7. **C)** Fluorescence image of microvessels (tdTomato, red) expressing CD31 (purple). **D)** Fluorescence timelapse MIP of microspheres (green) flowing through the open lumen of microvessels (tdTomato, red). **E)** Fluorescence timelapse images of dextran solution (green) retained in microvessels

(tdTomato, red). **B-E)** White dotted lines outline microposts. Blue arrows indicate the direction of interstitial flow. Scale bars indicate **B)** 200, **C)** 100, **D)** 200, and **E)** 50 μm.

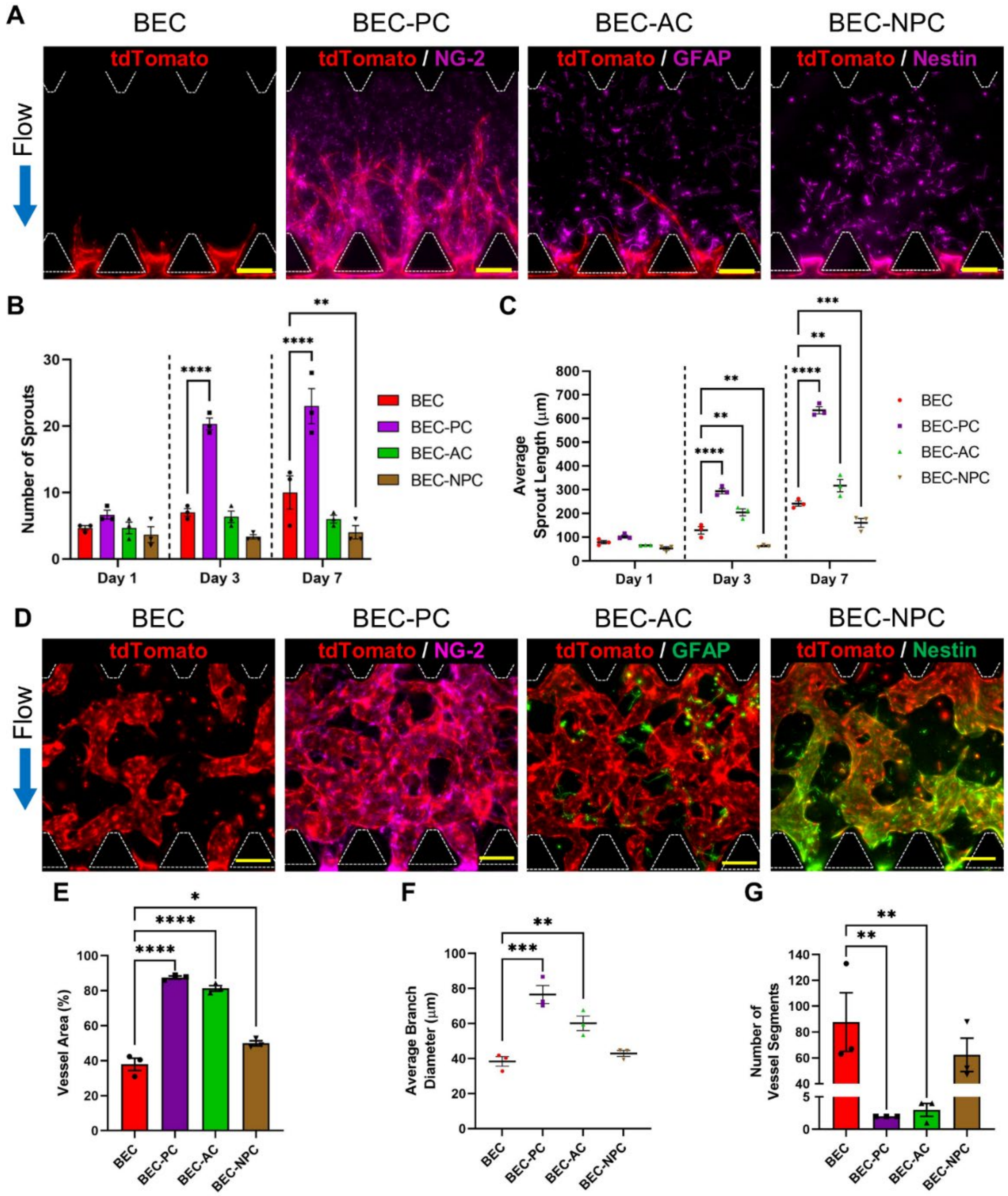

Figure S4. BEC angiogenesis and vasculogenesis assay with neural progenitor cells (NPCs) in MFDs. A) For the angiogenesis assay, BECs-tdT (tdTomato, red) were side-seeded in the fluidic channel of MFDs. BECs-tdT were promoted to grow sprouts into fibrin gels with no supporting cells (BEC condition), with PCs (BEC-PC condition, NG-2, purple), with ACs (BEC-AC condition, GFAP, purple), and with NPCs (BEC-NPC condition, nestin, purple). Fluorescence MIPs of angiogenic sprouts were acquired on Day 7. **B,C)** Graphs of the number of sprouts **(B)** and average sprout length **(C)** measured for BEC, BEC-PC, BEC-AC, and BEC-NPC conditions in the angiogenesis assay on Day 1, Day 3, and Day 7. Data from n = 3 MFDs. **D)** For the vasculogenesis assay, BECs-tdT (tdTomato, red) were

seeded in the hydrogel channel of MFDs. BECs-tdT were promoted to form vascular networks with no supporting cells (BEC condition), with PCs (BEC-PC condition, NG-2, purple), with ACs (BEC-AC condition, GFAP, green), and with NPCs (BEC-NPC condition, nestin, green). Fluorescence MIPs of vascular networks were acquired on Day 7. **A,D)** Scale bars indicate 200 µm. White dotted lines outline microposts. Blue arrows indicate the direction of interstitial flow. **E-G)** Graphs of the vessel area **(E)**, average branch diameter **(F)**, and number of vessel segments **(G)** measured for BEC, BEC-PC, BEC-AC, and BEC-NPC conditions in the vasculogenesis assay on Day 7. Data averaged from 3 ROIs from n = 3 MFDs. **B,C,E,F,G)** The data show mean value, error bars ± SEM, BEC condition (control) was compared to all other conditions, two-way **(B,C)** and one-way ANOVA **(E-G)** with Dunnett's test, absence of significance line indicates $p > 0.05$, * $p < 0.05$, ** $p < 0.01$, *** $p < 0.001$, **** $p < 0.0001$.

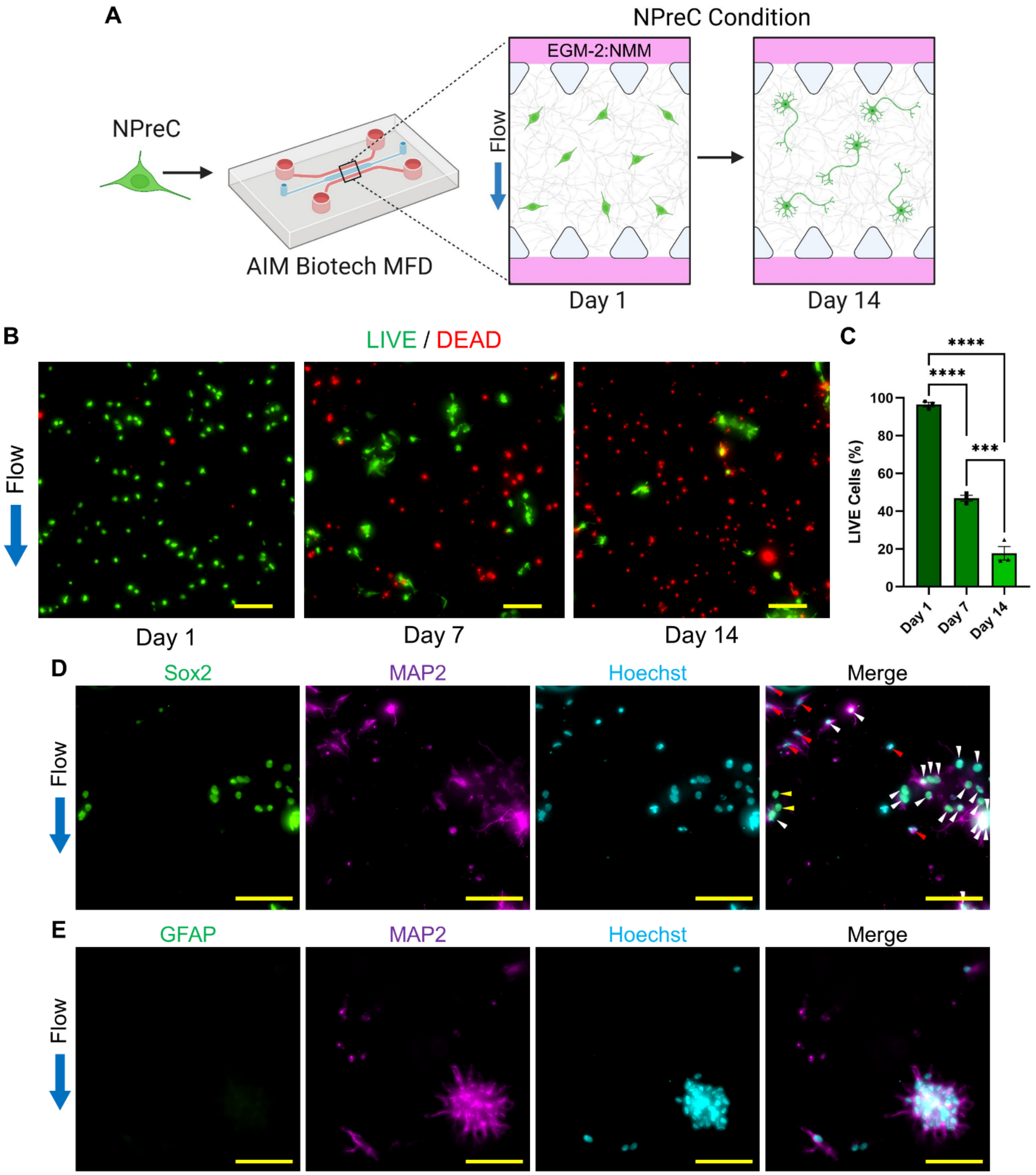

Figure S5. Viability of dispersed neuronal precursor cells (NPreCs) in MFD culture. A) Illustration of dispersed NPreC solo-culture in MFDs. NPreCs (2×10^6 cells/mL) were resuspended in fibrin gels (NPreC condition) within MFDs. Samples were cultured for two weeks in EGM-2:NMM under flow conditions. **B)** LIVE (green) / DEAD (red) MIPs of NPreCs on Day 1, Day 7, and Day 14. **C)** Graph of the corresponding percentage of LIVE NPreCs on Day 1, Day 7, and Day 14. The data show mean value, error bars ± SEM, n = 3, one-way ANOVA with Tukey's test, *** $p < 0.001$, **** $p < 0.0001$. **D,E)** Fluorescence images of dispersed NPreCs in MFDs on Day 14. Cells were labeled with Sox2 (**D**, green), MAP2 (**D,E**, purple), and GFAP (**E**, green). Nuclei were labeled with Hoechst (blue). Yellow, red, and white triangles indicate Sox2+/MAP2-, Sox2-/MAP2+, and Sox2+/MAP2+ nuclei, respectively. **B,D,E)** Scale bars indicate 100 μm. Blue arrows indicate the direction of interstitial flow.

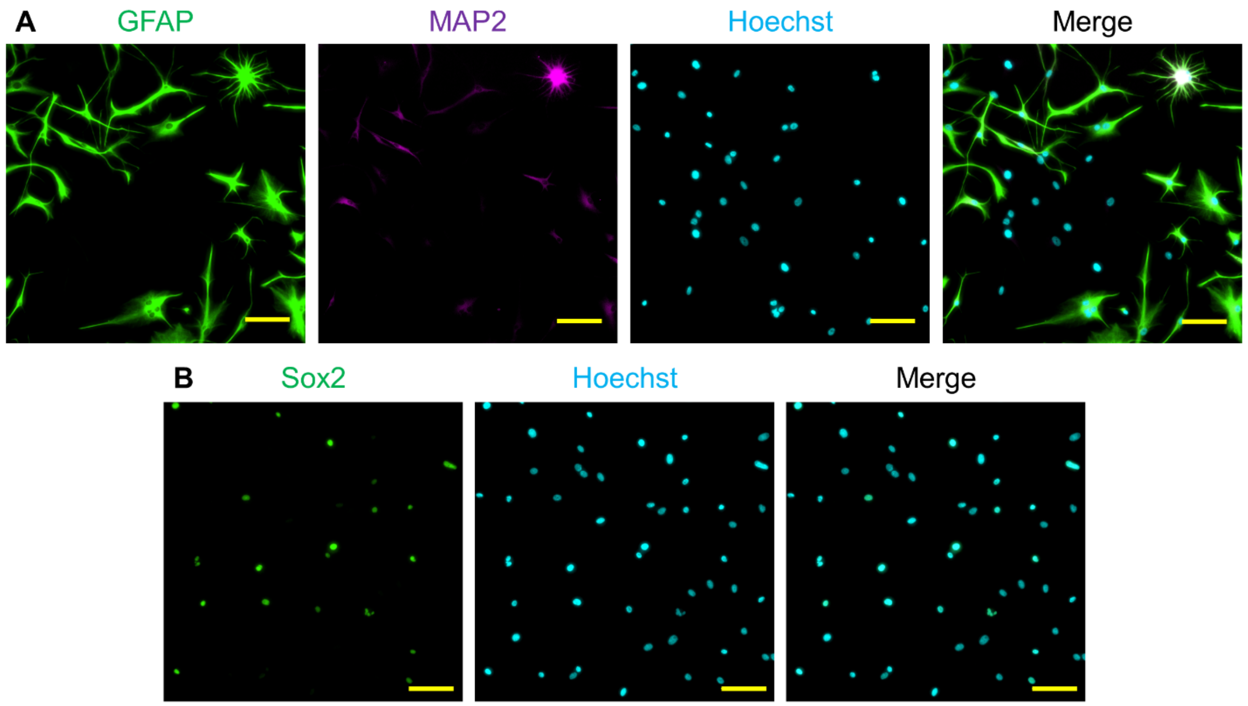

Figure S6. Immunocytochemistry of human astrocytes. A,B) Fluorescence images of astrocytes expressing GFAP (**A** green), MAP2 (**A**, purple), and Sox2 (**B**, green) on Day 3 of 2D cell culture. Nuclei are labeled with Hoechst (blue). Scale bars indicate 100 µm.

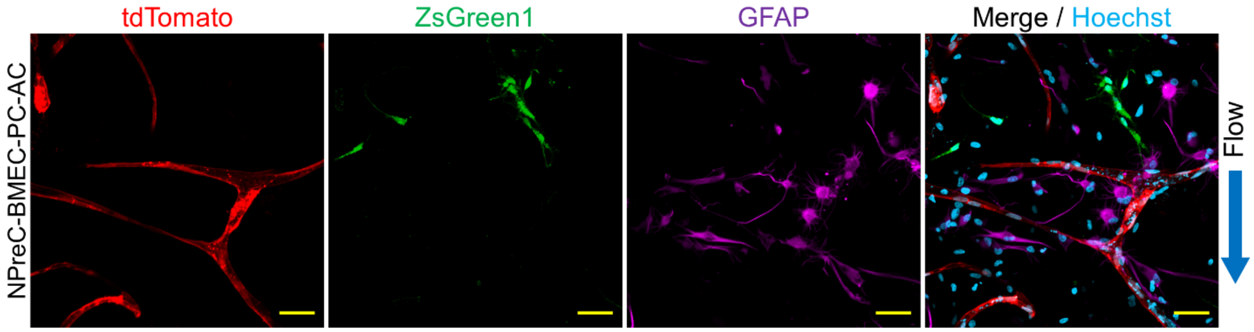

Figure S7. Immunocytochemistry of NPreCs with BMVNs in MFD culture. Fluorescence confocal MIPs of NPreCs-ZsG (ZsGreen1, green) cultured with BMECs-tdT (tdTomato, red), PCs, and ACs (NPreC-BMEC-PC-AC condition) on Day 14. ACs were stained for GFAP (purple). PCs were not labeled. Nuclei were labeled with Hoechst (blue). No examples of ZsGreen1+/GFAP+ cells were identified. Scale bars indicate 50 µm. Blue arrow indicates the direction of interstitial flow.

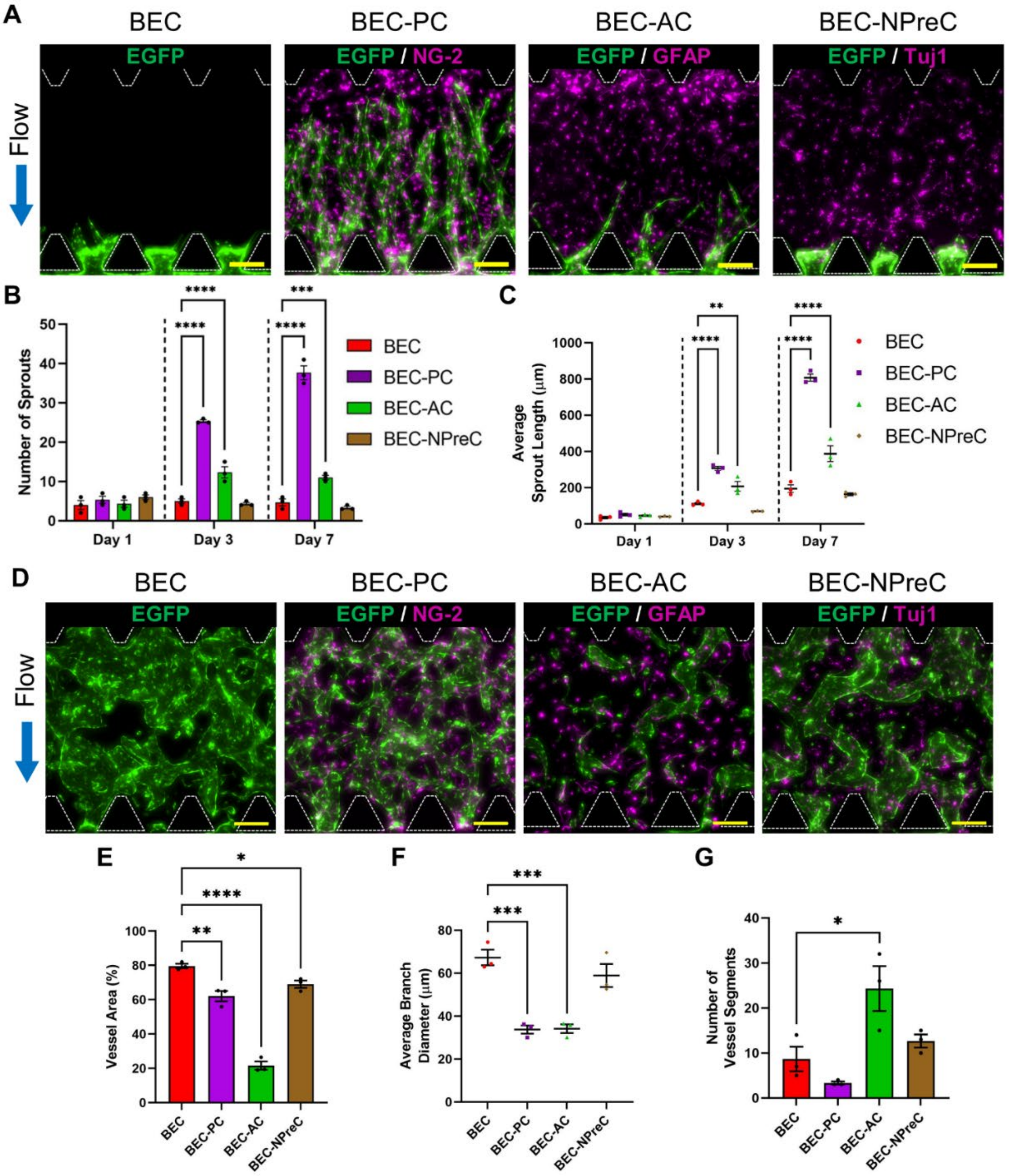

Figure S8. BEC angiogenesis and vasculogenesis assay with neuronal precursor cells (NPreCs) in MFDs. A) For the angiogenesis assay, BECs-EGFP (EGFP, green) were side-seeded in the fluidic channel of MFDs. BECs-EGFP were promoted to grow sprouts into fibrin gels with no supporting cells (BEC condition), with PCs (BEC-PC condition, NG-2, purple), with ACs (BEC-AC condition, GFAP, purple), and with NPreCs (BEC-NPreC condition, Tuj1, purple). Fluorescence MIPs of angiogenic sprouts were acquired on Day 7. **B,C)** Graphs of the number of sprouts **(B)** and average sprout length **(C)** measured for BEC, BEC-PC, BEC-AC, and BEC-NPreC conditions in the angiogenesis assay on Day 1, Day 3, and Day 7. Data from n = 3 MFDs. **D)** For the vasculogenesis assay, BECs-EGFP (EGFP, green) were seeded in the hydrogel channel of MFDs. BECs-EGFP were promoted to form vascular

networks with no supporting cells (BEC condition), with PCs (BEC-PC condition, NG-2, purple), with ACs (BEC-AC condition, GFAP, purple), and with NPreCs (BEC-NPreC condition, Tuj1, purple). Fluorescence MIPs of vascular networks were acquired on Day 7. **A,D)** Scale bars indicate 200 µm. White dotted lines outline microposts. Blue arrows indicate the direction of interstitial flow. **E-G)** Graphs of the vessel area **(E)**, average branch diameter **(F)**, and number of vessel segments **(G)** measured for BEC, BEC-PC, BEC-AC, and BEC-NPreC conditions in the vasculogenesis assay on Day 7. Data averaged from 3 ROIs from n = 3 MFDs. **B,C,E,F,G)** The data show mean value, error bars ± SEM, BEC condition (control) was compared to all other conditions, two-way **(B,C)** and one-way ANOVA **(E-G)** with Dunnett's test, absence of significance line indicates $p > 0.05$, * $p < 0.05$, ** $p < 0.01$, *** $p < 0.001$, **** $p < 0.0001$.

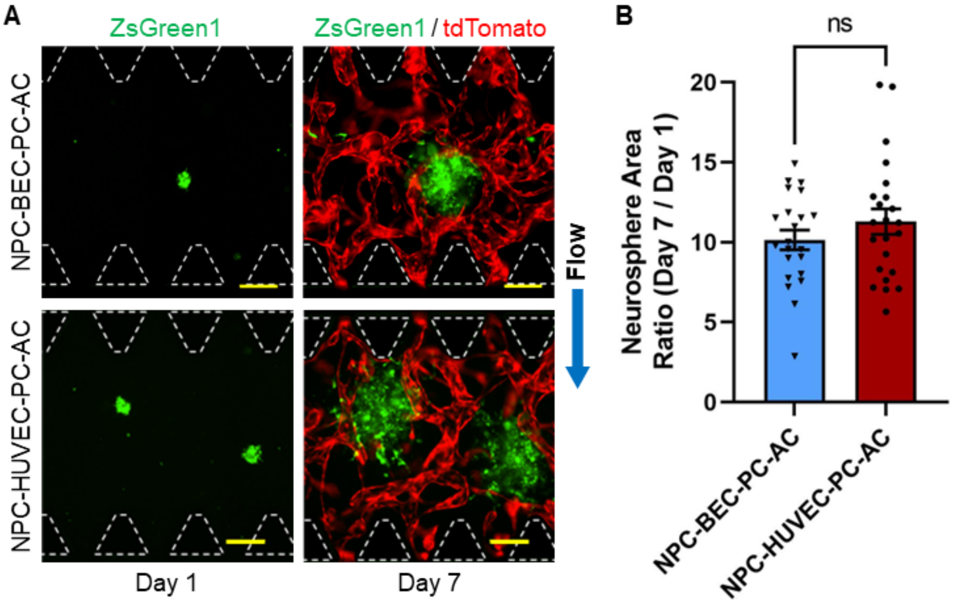

Figure S9. Effect of endothelial cell source on NPC neurosphere expansion in MFDs. This experiment was done in tandem to the experiment shown in Figure 3. Briefly, NPCs expressing ZsGreen1 were aggregated into neurospheres (NPC-ZsG-Nsphs) and cultured with either BECs-tdT, PCs, and ACs (NPC-BEC-PC-AC condition) or with HUVECs-tdT, PCs, and ACs (NPC-HUVEC-PC-AC condition) in fibrin gels within MFDs. Samples were cultured for one week in EGM-2:NPM-2 under flow conditions. **A)** Fluorescence MIPs of NPC-ZsG-Nsphs (ZsGreen1, green) cultured in NPC-BEC-PC-AC and NPC-HUVEC-PC-AC conditions on Day 1 and Day 7. BECs-tdT and HUVEC-tdT microvessels (tdTomato, red) were only imaged on Day 7. PCs and ACs are not shown. White dotted lines outline microposts. Blue arrow indicates the direction of interstitial flow for both conditions. Scale bars indicate 200 µm. **B)** Graph showing the Day7/Day1 neurosphere area ratio for both conditions. N ≥ 22 neurospheres were measured from n = 3 MFDs. The data show mean value, error bars ± SEM, Welch's t-test, ns $p > 0.05$.

S3. Supplementary Tables

Target Species	Target Protein	Host Species	Company	Catalog Number	Concentration
Human	Nestin	Rabbit	BioLegend	841901	1:200
Human	Sox2	Rabbit	Invitrogen	PA5-85144	1:100
Human	GFAP	Rabbit	Invitrogen	PA1-10019	1:1000
Human	Neuron-Specific Tuj1	Mouse	R&D Systems	MAB1195	1:100
Human	MAP2	Mouse	Invitrogen	13-1500	1:400
Human	NG-2	Mouse	eBioscience	14-6504-80	1:100
Human	CD31	Mouse	BD Biosciences	550389	1:50
Human	Ki67	Mouse	Invitrogen	14-5699-82	1:500

Table S1. Primary Antibodies for Immunocytochemistry.

Host Species	Target Species	Excitation Wavelength	Company	Catalog Number	Concentration
Goat	Rabbit	488	Invitrogen	A11034	1:500
Goat	Rabbit	647	Invitrogen	A32733	1:500
Goat	Mouse	647	Invitrogen	A21236	1:500

Table S2. Secondary Antibodies for Immunocytochemistry.

Abbreviation	Cell Types	Cell Arrangement	Interstitial Flow	Cell Culture Medium
NPC	NPCs cultured alone	Dispersed NPCs or NPC-Nsphs	Flow or Static	EGM-2:NPM-2 or NPM-2
NPC-PC	NPCs cultured with PCs	Dispersed NPCs or NPC-Nsphs	Flow	EGM-2:NPM-2
NPC-AC	NPCs cultured with ACs	Dispersed NPCs or NPC-Nsphs	Flow	EGM-2:NPM-2
NPC-BEC	NPCs cultured with BECs	Dispersed NPCs or NPC-Nsphs	Flow	EGM-2:NPM-2
NPC-BEC-PC-AC	NPCs cultured with BECs, PCs, and ACs	Dispersed NPCs or NPC-Nsphs	Flow or Static	EGM-2:NPM-2
NPC-HUVEC-PC-AC	NPCs cultured with HUVECs, PCs, and ACs	NPC-Nsphs	Flow	EGM-2:NPM-2
NPreC	NPreCs cultured alone	Dispersed NPreCs or NPreC-Nsphs	Flow	EGM-2:NMM
NPreC-PC	NPreCs cultured with PCs	Dispersed NPreCs or NPreC-Nsphs	Flow	EGM-2:NMM
NPreC-AC	NPreCs cultured with ACs	Dispersed NPreCs or NPreC-Nsphs	Flow	EGM-2:NMM
NPreC-BEC	NPreCs cultured with BECs	Dispersed NPreCs or NPreC-Nsphs	Flow	EGM-2:NMM
NPreC-BEC-PC-AC	NPreCs cultured with BECs, PCs, and ACs	Dispersed NPreCs or NPreC-Nsphs	Flow or Static	EGM-2:NMM
NPreC-BMEC-PC-AC	NPreCs cultured with BMECs, PCs, and ACs	Dispersed NPreCs	Flow or Static	EGM-2:NMM
BEC	BECs cultured alone	BECs in fluidic channel or fibrin gel	Flow	EGM-2:NPM-2 or EGM-2:NMM
BEC-PC	BECs cultured with PCs	BECs in fluidic channel or fibrin gel	Flow	EGM-2:NPM-2 or EGM-2:NMM
BEC-AC	BECs cultured with ACs	BECs in fluidic channel or fibrin gel	Flow	EGM-2:NPM-2 or EGM-2:NMM
BEC-NPC	BECs cultured with NPCs	BECs in fluidic channel or fibrin gel	Flow	EGM-2:NPM-2
BEC-NPreC	BECs cultured with NPreCs	BECs in fluidic channel or fibrin gel	Flow	EGM-2:NMM

Table S3. List of Experimental Conditions.

S4. Supplementary Movie Captions

Movie S1. Perfused brain microvascular networks with neuronal precursor cell neurospheres (NPreC-Nsphs). Associated with Fig. 7K in main text. Fluorescence timelapse image of microspheres (green) flowing through brain microvessels (tdTomato, red) supporting NPreC-Nsphs (Tuj1, purple) cultured under flow conditions on Day 7. PCs and ACs are not shown.
